# Primary Health Care Services and Continuity of Care Are Associated With Better Health Outcomes in the Older Population

**DOI:** 10.1111/jgs.70465

**Published:** 2026-04-28

**Authors:** Gillian E. Caughey, Johannes Schwabe, Brian W. Pulling, Maria Crotty, Helena Williams, Andrew Kellie, Gillian Harvey, Steve L. Wesselingh, David Roder, Krystal‐Lee Nixon, Janet K. Sluggett, Monica Cations, Tiffany K. Gill, Jyoti Khadka, Megan Corlis, Carolyn Dawkins, Marilyn von Thien, Maria C. Inacio

**Affiliations:** ^1^ Registry of Senior Australians Research Centre South Australian Health and Medical Research Institute Adelaide South Australia Australia; ^2^ Registry of Senior Australians Research Centre, Caring Futures Institute, College of Nursing and Health Sciences Flinders University Bedford Park South Australia Australia; ^3^ College of Medicine and Public Health Flinders University Adelaide South Australia Australia; ^4^ Southern Adelaide Local Health Network, SA Health Adelaide South Australia Australia; ^5^ Signal Health Newton Newton South Australia Australia; ^6^ Caring Futures Institute, College of Nursing and Health Sciences Flinders University Bedford Park South Australia Australia; ^7^ South Australian Health and Medical Research Institute Adelaide South Australia Australia; ^8^ UniSA Allied Health and Human Performance University of South Australia Adelaide Australia; ^9^ SA Health Dental Service Adelaide South Australia Australia; ^10^ College of Education, Psychology and Social Work Flinders University Bedford Park South Australia Australia; ^11^ Adelaide Medical School, Faculty of Health and Medical Sciences The University of Adelaide Adelaide South Australia Australia; ^12^ Australian Nursing and Midwifery Federation (SA Branch) Adelaide South Australia Australia; ^13^ ECH Inc Adelaide South Australia Australia

**Keywords:** continuity of patient care, general practice, hospitalization, long‐term care, primary health care

## Abstract

**Background:**

Older people's preference is to age in place. Optimizing the delivery of high‐quality home‐based primary care for this growing population is a priority for healthcare systems worldwide. Provision of primary care is essential to support the health and wellbeing of the older population, yet there is a lack of high‐quality evidence quantifying the effects of primary care services for the older population. This study aimed to examine the association of continuity of primary care and primary health care patterns on the risk of mortality and hospitalization in the older population.

**Methods:**

A retrospective cohort study was conducted between 1 July 2016 and 31 December 2019 of 120,522 older people (≥ 65 years) living in the community receiving long‐term care. Continuity of primary care and patterns of primary care service utilization on the risk of mortality and nine hospitalization‐related outcomes were examined. Propensity score methods for confounding adjustment and survival analyses were employed.

**Results:**

Compared with seeing a new primary care physician (*n* = 25,213, 30%), seeing a known primary care physician (*n* = 41,309, 49.1%) was associated with lower risks of hospitalizations ranging from 18% reduction for medication‐related hospitalization (sHR = 0.82, 95% CI 0.74–0.91) to 28% reduction for *fractures* (*s*HR *= 0.72, 95% CI 0.66–0.78*). The care pattern of high preventive primary care service use (*n* = 34,021, 62.4%) was associated with lower risks of hospitalization ranging from 15% for ED presentations (sHR = 0.85, 95% CI 0.80–0.92) to 36% for pressure injury‐related hospitalization (sHR = 0.64, 95% CI 0.52–0.80) compared to high overall primary care use (*n* = 5293, 9.7%).

**Conclusions:**

Care patterns focusing on prevention and disease management, and continuity of primary care were associated with more favorable health outcomes among older people aiming to age in place.

## Summary

- Key points
  ○ In a national cohort of 120,522 older Australians receiving long‐term home care, a preventive, multidisciplinary primary care utilization pattern (high use of long‐GP attendances, care management plans, and allied health, and low use of after‐hours and urgent GP attendances) was associated with a 15%–36% lower risk of hospitalization compared to high overall primary care service utilization.
  ○ Continuity of primary care with a known or usual GP was associated with an 18%–28% lower risk of ED presentations or hospitalizations compared to seeing a new GP.
  ○ Findings highlight the need for clear policy and practice change to support continuity of care, together with a multidisciplinary primary care team that focuses on individual care needs and priorities to enable our older populations to not only successfully age in place, but to age well.
- Why does this paper matter?
  ○ Evidence guiding which primary care services and delivery patterns improve outcomes for older people receiving long‐term home‐based care has been limited. Using linked national data, we identify continuity of primary care and prevention‐focused multidisciplinary care as actionable levers, associated with reduced risks of mortality and hospitalizations, informing models of care and policy.

## 1 Introduction

With aging populations worldwide, home‐based long‐term care aims to support older people to remain living at home and in the community for as long as possible [1, 2]. In accord with older people's preference to “age in place,” the demand for home care services to provide social and health care supports is increasing [1, 2]. Aging in place is defined as continued living in the community, adapting to changes and needs associated with aging, while maintaining social supports and some level of independence [3, 4]. Together with the global increase in older populations and increasing demand for home‐based care, optimizing the delivery of high‐quality health care to older people is a priority for healthcare systems worldwide [1, 2]. Over the past decade, the provision of long‐term home care to the older population has doubled in many countries [5, 6]. However, concerns have been raised regarding the quality of home care services received, which may not be meeting individuals social and health care needs [7, 8, 9]. A higher incidence of adverse events (e.g., hospitalizations for falls, dementia‐related events, pressure injuries), compared to individuals in long‐term care facilities has also been reported [9]. With the continued growing need for home care services, health and aged care systems internationally need to respond to the increasing demands, while ensuring that the care delivered is safe and effective in supporting older people's health and social care needs to successfully remain at home [2, 10].

Provision of primary health care is an integral component to successful aging in place, supporting the health and wellbeing of the older population [11]. However, there is a lack of high‐quality evidence regarding the benefits of primary care services for older people receiving long‐term home care. Further, there is underutilization of key primary care services concordant with the complex care needs of individuals in this setting [12]. The majority (80%) of the older population will have multimorbidity that requires ongoing care to reduce disease symptoms and burdens, delay functional and psychological consequences, alongside the biological aging trajectory [13, 14]. A recent international comparison of health systems highlighted that primary care‐oriented health systems that included primary care physicians as a first point of contact, better care continuity, and stronger financial incentives directed to primary care physicians to improve quality were associated with fewer potentially avoidable hospitalizations [15]. Current models of health care for the older population are based on episodic, disease‐focused, and siloed care delivery that does not meet the heterogeneity of the older population's care needs and individual priorities.

The aim of this study was to investigate the association of continuity of care, and patterns of primary health care utilization with health outcomes including mortality and hospitalization, in the older population receiving long‐term home care. Secondary aims included examination of the associations of individual primary care services with health outcomes.

## 2 Methods

### 2.1 Study Setting, Design, and Data Source

We conducted a population‐based retrospective cohort study of older people receiving long‐term home care supports using the National Historical Cohort of the Registry of Senior Australians (ROSA) [16]. The ROSA National Historical Cohort contains integrated national health, aged care and social welfare information for ~3.5 million Australians. Australia has universal health care and government subsidies for long‐term care services, with most individuals accessing health and long‐term care services through the programs captured within the employed datasets (Supporting Informations) [17, 18]. Australia's Home Care Packages fund bundled in‐home personal, social and clinical supports across four care levels (1–4) aligned to assessed need, ranging from entry‐level assistance for basic daily activities (Level 1) to comprehensive support for people with high and complex care needs (Level 4) [19, 20]. This model is comparable to US Personal Care Services [21] and the UK's Home Care Services [22].

### 2.2 Cohort

**FIGURE 1:**
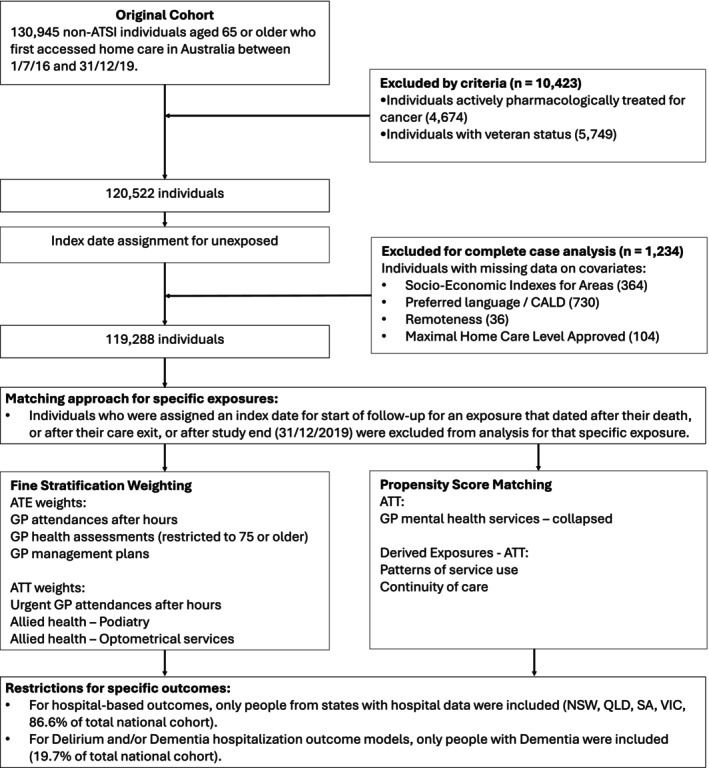
Study cohort(s) selection flowchart.

Older individuals aged ≥ 65 years old receiving long‐term home care in Australia between 01/07/2016 and 12/31/2019 who were not Aboriginal or Torres Strait Islander were included (*N* = 130,945). Individuals receiving pharmacological treatment for cancer (*N* = 4674) were excluded. Department of Veterans' Affairs card holders (*N* = 5749) were excluded because of different health and long‐term care entitlements [23]. The final cohort included *N* = 120,522 older individuals (Figure 1).

### 2.3 Exposures of Interest

Continuity of primary care with primary care physician (referred to general practitioner, GP) and patterns of primary health care service utilization (from 13 individual primary care services including allied health, mental health care services), were the primary exposures examined. Secondary aim exposures included the utilization of 13 individual primary care services (Supporting Informations, Table S1). All services were examined as binary variables.

The date of first service access (index date), for each exposure independently was the start of the study follow up. An established method was used to assign index dates for follow up for each control (Supporting Informations) [24].

Continuity of primary care was classified as usual GP, known GP, or new GP by comparing the most‐frequently seen GP in the first six months after home‐care entry with GPs seen in the preceding two years, per prior methods [25]. The categories were “new GP” (GP most frequently seen after home care entry was never seen prior to entry), “known GP” (GP most frequently seen after home care entry was seen at least once prior to entry), and “usual GP” (GP most frequently seen did not change from before to after home care entry). Analyses were restricted to 84,063 individuals alive and in care at six months.

Patterns of primary health care utilization in the year after entry were estimated using latent class analysis models (Supporting Informations). To identify classes of individuals with distinct patterns of primary care utilization in the first year after entry, the cohort for analysis included 54,489 individuals who were alive and in home care one year after entry. Shown in Table S2 and Figure S1 are the summaries of patterns of primary health care service utilization.

### 2.4 Outcomes of Interest

The outcomes of interest examined within one year after the exposure were: mortality, premature mortality, ED presentations, unplanned hospitalizations, potentially preventable hospitalizations, hospitalizations for falls, hospitalizations for fractures, hospitalizations for medication‐related adverse events, hospitalizations for delirium and/or dementia‐related issues, hospitalizations with a diagnosis of pressure injury, hospitalizations with a diagnosis of weight loss, and hospitalizations with a diagnosis of malnutrition (Table S3 for coding). Study follow‐up for each exposure started on the index date (or after the exposure periods for continuity of care and patterns of primary care utilization, which were 182 days and 365 days, respectively) and ended at the outcome of interest, death, home care exit, end of the study period (12/31/2019), or 365 days.

### 2.5 Covariates

We adjusted for demographics, clinical burden (Rx‐Risk‐V pharmaceutical based comorbidity index [26] and condition indicators), socio‐economic context, care‐need indicators, provider characteristics, and prior utilization during the one‐year look‐back (hospitalisations/ED presentation counts and length of stay), including history of the exposure service (Supporting Informations).

### 2.6 Statistical Analysis

We used propensity score‐based confounding adjustment, applying fine‐stratification weighting when overlap was adequate and nearest‐neighbor matching otherwise. Propensity scores (logistic models) included individual, provider, and prior‐utilization covariates. Further details are described in the Supporting Informations. Due to the small number of missing data (*n* = 1234/120522, 1.0%) a complete case analysis was conducted (Figure 1).

For each outcome, the incidence rate per 100 recipient‐years of follow up was estimated using the total people‐days at risk as the denominator and the number of outcome events multiplied by 36,500 as the numerator. Cumulative incidence within one year of service access was estimated based on the Kaplan–Meier estimator for outcomes without competing risk and the Aalen‐Johansen estimator for outcomes with competing risk. Cox regression models were employed to examine the risk of mortality. Hazard ratios (HR) and 95% confidence intervals (CIs) are reported. Fine and Gray models estimated the risk of hospitalization‐related outcomes while accounting for the competing risk of mortality. Health care utilization related covariates were included in the hospitalization‐specific outcomes analysis. Sub‐distribution hazards (sHR) and 95% CIs are reported. The Benjamini‐Hochberg method was employed to control the false discovery rate.

### 2.7 Sensitivity Analysis

Sensitivity analyses were conducted to examine the robustness of the results against additional covariates in the propensity score and stratifying by care levels, stratifying by the presence of dementia or diabetes, and delaying outcome ascertainment to 30 days post‐exposure period for individual health services (potentially addressing reverse causality bias, Supporting Informations, Figure S2).

STATA (latent class analysis), SAS (Fine and Gray models), and R (all other analyses) were used for the analyses.

## 3 Results

### 3.1 Study Cohorts

Of the 120,522 older people receiving long‐term home care studied, 60.9% (*n* = 73,515) were female, the mean age was 81.8 years (standard deviation = 7.3), and 19.7% (*n* = 23,686) were living with dementia.

The characteristics of the unmatched and matched cohorts used for each exposure analysis are shown in Table 1 for continuity of care and Tables S4–S11 for the other exposures. The crude event rate per 100 recipient years and cumulative incidences within one year of follow‐up for all outcomes by each matched cohort exposure group are shown in Table S12.

**TABLE 1:** Continuity of care: Baseline characteristics of study cohort before and after matching.

Exposure status	Overall cohort				Matched cohort 1: Known versus new GP^a^			Matched cohort 2: Usual versus new GP^a^		
	Overall cohort	New GP	Known GP	Usual GP	New GP	Known GP	SMD^b^	New GP	Usual GP	SMD^b^
Total, *N* (%)	84,063	25,213	41,309	17,541	17,126	13,069		17,112	7885	
Individual characteristics										
Male sex, *N* (%)	31,739 (37.8)	9389 (37.2)	15,633 (37.8)	6717 (38.3)	6231 (36.4)	4909 (37.6)	0.004	6231 (36.4)	2854 (36.2)	0.002
Age at entry, Mean (SD), years	81.5 (7.3)	81.5 (7.4)	81.5 (7.2)	81.3 (7.3)	81.3 (7.3)	81.1 (7.3)	−0.007	81.3 (7.3)	81 (7.3)	−0.009
Culturally and linguistical diverse, *N* (%)	11,653 (13.9)	3222 (12.8)	6006 (14.5)	2425 (13.8)	2123 (12.4)	1488 (11.4)	0.002	2123 (12.4)	1009 (12.8)	0.001

Partner status, *N* (%)										
Divorced	7597 (9.0)	2421 (9.6)	3604 (8.7)	1572 (9)	1618 (9.4)	1206 (9.2)	−0.002	1618 (9.4)	696 (8.8)	−0.002
Married	40,272 (47.9)	11,775 (46.7)	20,286 (49.1)	8211 (46.8)	8108 (47.3)	6068 (46.4)	0.005	8108 (47.3)	3593 (45.6)	0.001
Never married	4939 (5.9)	1546 (6.1)	2305 (5.6)	1088 (6.2)	1050 (6.1)	823 (6.3)	−0.002	1050 (6.1)	518 (6.6)	< 0.001
Separated	648 (0.8)	177 (0.7)	329 (0.8)	142 (0.8)	135 (0.8)	121 (0.9)	R^d^	135 (0.8)	74 (0.9)	R^d^
Unable to determine	932 (1.1)	292 (1.2)	427 (1)	213 (1.2)	204 (1.2)	144 (1.1)	R^d^	204 (1.2)	98 (1.2)	R^d^
Widowed	29,675 (35.3)	9002 (35.7)	14,358 (34.8)	6315 (36)	6011 (35.1)	4707 (36)	R^d^	6011 (35.1)	2906 (36.9)	R^d^

*Select health conditions, N* (*%*)										
Dementia	15,097 (18.0)	4849 (19.2)	7128 (17.3)	3120 (17.8)	2903 (17)	2399 (18.4)	< 0.001	2903 (17)	1397 (17.7)	< 0.001
History pressure injuries	3987 (4.7)	1222 (4.8)	1904 (4.6)	861 (4.9)	794 (4.6)	610 (4.7)	0.001	794 (4.6)	383 (4.9)	< 0.001
Incontinence	11,374 (13.5)	3416 (13.5)	5476 (13.3)	2482 (14.1)	2270 (13.3)	1804 (13.8)	< 0.001	2270 (13.3)	1085 (13.8)	−0.001
History of cancer	14,688 (17.5)	4357 (17.3)	7336 (17.8)	2995 (17.1)	2931 (17.1)	2163 (16.6)	0.001	2931 (17.1)	1294 (16.4)	< 0.001
History of pain	38,741 (46.1)	10,997 (43.6)	19,460 (47.1)	8284 (47.2)	7602 (44.4)	5892 (45.1)	0.009	7602 (44.4)	3592 (45.6)	0.004
Chronic respiratory disease	24,850 (29.6)	6946 (27.5)	12,775 (30.9)	5129 (29.2)	4665 (27.2)	3687 (28.2)	0.006	4665 (27.2)	2159 (27.4)	0.001
Heart failure	17,409 (20.7)	4712 (18.7)	8909 (21.6)	3788 (21.6)	3100 (18.1)	2497 (19.1)	0.008	3100 (18.1)	1586 (20.1)	0.005
Depression	34,396 (40.9)	9887 (39.2)	17,336 (42)	7173 (40.9)	6621 (38.7)	5283 (40.4)	0.007	6621 (38.7)	3194 (40.5)	0.004
Diabetes	22,781 (27.1)	6354 (25.2)	11,636 (28.2)	4791 (27.3)	4228 (24.7)	3358 (25.7)	0.006	4228 (24.7)	2056 (26.1)	0.001
Ischemic heart disease/hypertension	59,673 (71.0)	17,385 (69)	29,782 (72.1)	12,506 (71.3)	11,859 (69.2)	9035 (69.1)	0.004	11,859 (69.2)	5495 (69.7)	0.003
Osteoporosis	23,889 (28.4)	6784 (26.9)	12,154 (29.4)	4951 (28.2)	4598 (26.8)	3426 (26.2)	0.002	4598 (26.8)	2124 (26.9)	0.003
Mean number additional health conditions (SD)^c^	2.5 (1.6)	2.3 (1.6)	2.6 (1.6)	2.6 (1.6)	2.3 (1.5)	2.4 (1.5)	0.040	2.3 (1.5)	2.5 (1.5)	0.023

SEIFA index of relative socioeconomic disadvantage quintile, *N* (%)							−0.003			0.009
1 – disadvantaged	17,022 (20.2)	5083 (20.2)	8408 (20.4)	3531 (20.1)	3549 (20.7)	2672 (20.4)		3549 (20.7)	1609 (20.4)	
2	17,538 (20.9)	5439 (21.6)	8643 (20.9)	3456 (19.7)	3800 (22.2)	2934 (22.5)		3800 (22.2)	1599 (20.3)	
3	17,399 (20.7)	5199 (20.6)	8567 (20.7)	3633 (20.7)	3495 (20.4)	2721 (20.8)		3495 (20.4)	1612 (20.4)	
4	15,306 (18.2)	4440 (17.6)	7592 (18.4)	3274 (18.7)	2950 (17.2)	2332 (17.8)		2950 (17.2)	1480 (18.8)	
5 – advantaged	16,798 (20.0)	5052 (20)	8099 (19.6)	3647 (20.8)	3332 (19.5)	2410 (18.4)		3332 (19.5)	1585 (20.1)	

SEIFA index of education and occupation quintiles, *N* (%)							−0.005			0.009
1 – Low	17,899 (21.3)	5391 (21.4)	8834 (21.4)	3674 (20.9)	3742 (21.8)	2896 (22.2)		3742 (21.8)	1693 (21.5)	
2	15,936 (19.0)	4897 (19.4)	7772 (18.8)	3267 (18.6)	3401 (19.9)	2555 (19.6)		3401 (19.9)	1498 (19)	
3	16,125 (19.2)	4838 (19.2)	8032 (19.4)	3255 (18.6)	3309 (19.3)	2671 (20.4)		3309 (19.3)	1440 (18.3)	
4	14,238 (16.9)	4180 (16.6)	7033 (17)	3025 (17.2)	2790 (16.3)	2140 (16.4)		2790 (16.3)	1371 (17.4)	
5—High	19,865 (23.6)	5907 (23.4)	9638 (23.3)	4320 (24.6)	3884 (22.7)	2807 (21.5)		3884 (22.7)	1883 (23.9)	

Care level at entry, *N* (%)							0.026			0.022
Level 1	11,784 (14.0)	4567 (18.1)	5291 (12.8)	1926 (11)	3287 (19.2)	1010 (7.7)		3287 (19.2)	382 (4.8)	
Level 2	53,837 (64.0)	15,607 (61.9)	26,592 (64.4)	11,638 (66.3)	10,785 (63)	9400 (71.9)		10,785 (63)	5993 (76)	
Level 3	10,798 (12.8)	2859 (11.3)	5829 (14.1)	2110 (12)	1657 (9.7)	1662 (12.7)		1657 (9.7)	767 (9.7)	
Level 4	7644 (9.1)	2180 (8.6)	3597 (8.7)	1867 (10.6)	1397 (8.2)	997 (7.6)		1397 (8.2)	743 (9.4)	

Maximally approved care level, *N* (%)							−0.005			−0.006
No approved level	8149 (9.7)	2600 (10.3)	4003 (9.7)	1546 (8.8)	1760 (10.3)	1186 (9.1)		1760 (10.3)	829 (10.5)	
Level 1	1379 (1.6)	315 (1.2)	796 (1.9)	268 (1.5)	197 (1.2)	116 (0.9)		197 (1.2)	16 (0.2)	
Level 2	34,409 (40.9)	10,374 (41.1)	16,324 (39.5)	7711 (44)	7599 (44.4)	5410 (41.4)		7599 (44.4)	3583 (45.4)	
Level 3	13,462 (16.0)	4219 (16.7)	7888 (19.1)	1355 (7.7)	2643 (15.4)	2128 (16.3)		2643 (15.4)	246 (3.1)	
Level 4	26,664 (31.7)	7705 (30.6)	12,298 (29.8)	6661 (38)	4927 (28.8)	4229 (32.4)		4927 (28.8)	3211 (40.7)	
Approval for long‐term care facility, *N* (%)	58,920 (70.1)	17,895 (71)	28,998 (70.2)	12,027 (68.6)	11,826 (69.1)	9269 (70.9)	−0.003	11,826 (69.1)	5527 (70.1)	−0.005
Approval for respite care, *N* (%)	75,218 (89.5)	22,631 (89.8)	36,937 (89.4)	15,650 (89.2)	15,240 (89)	11,770 (90.1)	−0.004	15,240 (89)	7137 (90.5)	< 0.001
Wait time for care days, logarithmized median [IQR]	5.9 [5.1, 6.4]	5.8 [5.1, 6.6]	6 [5.4, 6.4]	5.3 [3.6, 6.2]	5.8 [5, 6.6]	5.9 [5.2, 6.4]	0.027	5.8 [5, 6.6]	5.1 [3.9, 6.2]	−0.045

Provider characteristics										
State of service, *N* (%)										
Australian Capital Territory	1134 (1.3)	375 (1.5)	501 (1.2)	258 (1.5)	264 (1.5)	196 (1.5)	< 0.001	264 (1.5)	142 (1.8)	< 0.001
New South Wales	28,955 (34.4)	8726 (34.6)	14,304 (34.6)	5925 (33.8)	5993 (35)	4511 (34.5)	R	5993 (35)	2531 (32.1)	R
Northern Territory	157 (0.2)	54 (0.2)	56 (0.1)	47 (0.3)	34 (0.2)	24 (0.2)	< 0.001	34 (0.2)	18 (0.2)	< 0.001
Queensland	17,863 (21.2)	5572 (22.1)	8863 (21.5)	3428 (19.5)	3750 (21.9)	2906 (22.2)	−0.003	3750 (21.9)	1655 (21)	−0.005
South Australia	6504 (7.7)	1883 (7.5)	3104 (7.5)	1517 (8.6)	1222 (7.1)	974 (7.5)	0.000	1222 (7.1)	669 (8.5)	0.003
Tasmania	1941 (2.3)	571 (2.3)	955 (2.3)	415 (2.4)	419 (2.4)	313 (2.4)	0.000	419 (2.4)	200 (2.5)	0.001
Victoria	19,908 (23.7)	5683 (22.5)	9903 (24)	4322 (24.6)	3854 (22.5)	2851 (21.8)	0.003	3854 (22.5)	1860 (23.6)	0.007
Western Australia	7601 (9.0)	2349 (9.3)	3623 (8.8)	1629 (9.3)	1590 (9.3)	1294 (9.9)	−0.001	1590 (9.3)	810 (10.3)	< 0.001

Remoteness, *N* (%)										
Major Cities	57,034 (67.8)	16,315 (64.7)	28,282 (68.5)	12,437 (70.9)	10,761 (62.8)	8130 (62.2)	R	10,761 (62.8)	5269 (66.8)	R
Inner Regional	21,264 (25.3)	6972 (27.7)	10,487 (25.4)	3805 (21.7)	5050 (29.5)	3916 (30)	−0.004	5050 (29.5)	1965 (24.9)	−0.011
Outer Regional	5307 (6.3)	1770 (7)	2361 (5.7)	1176 (6.7)	1218 (7.1)	952 (7.3)	−0.001	1218 (7.1)	605 (7.7)	< 0.001
Remote	364 (0.4)	126 (0.5)	142 (0.3)	96 (0.5)	77 (0.4)	53 (0.4)	0.000	77 (0.4)	36 (0.5)	< 0.001
Very Remote	94 (0.1)	30 (0.1)	37 (0.1)	27 (0.2)	20 (0.1)	18 (0.1)	−^e^	20 (0.1)	10 (0.1)	−^e^
Missing	0 (0.0)	0 (0)	0 (0)	0 (0)	0 (0)	0 (0)		0 (0)	0 (0)	

Ownership, *N* (%)										
Government	5658 (6.7)	1757 (7)	2794 (6.8)	1107 (6.3)	1226 (7.2)	919 (7)	< 0.001	1226 (7.2)	517 (6.6)	< 0.001
Not‐for‐profit	60,385 (71.8)	17,869 (70.9)	28,850 (69.8)	13,666 (77.9)	12,183 (71.1)	9404 (72)	R	12,183 (71.1)	6354 (80.6)	R
Private	18,020 (21.4)	5587 (22.2)	9665 (23.4)	2768 (15.8)	3717 (21.7)	2746 (21)	0.002	3717 (21.7)	1014 (12.9)	−0.014

Abbreviations: *R*, Reference; SD, Standard deviation; SEIFA, Socio‐Economic Indexes for Areas.

^a^ Fine stratification weighted with 50 strata and weights targeting the average treatment effect.

^b^ Standardized mean differences < 0.10 are considered good balance.

^c^ Excludes health conditions listed in the table.

^d^ Combined reference category.

^e^ Very remote and remote categories were collapsed for the purpose of weighting due to small cell numbers in very remote categories.

### 3.2 Continuity of Care

In the six months following study entry (*n* = 84,063), 30.0% (*n* = 25,213) saw a new GP, 49.1% (*n* = 41,309) saw a known GP, and 20.9% (*n* = 17,541) saw their usual GP (Table 1).

**FIGURE 2:**
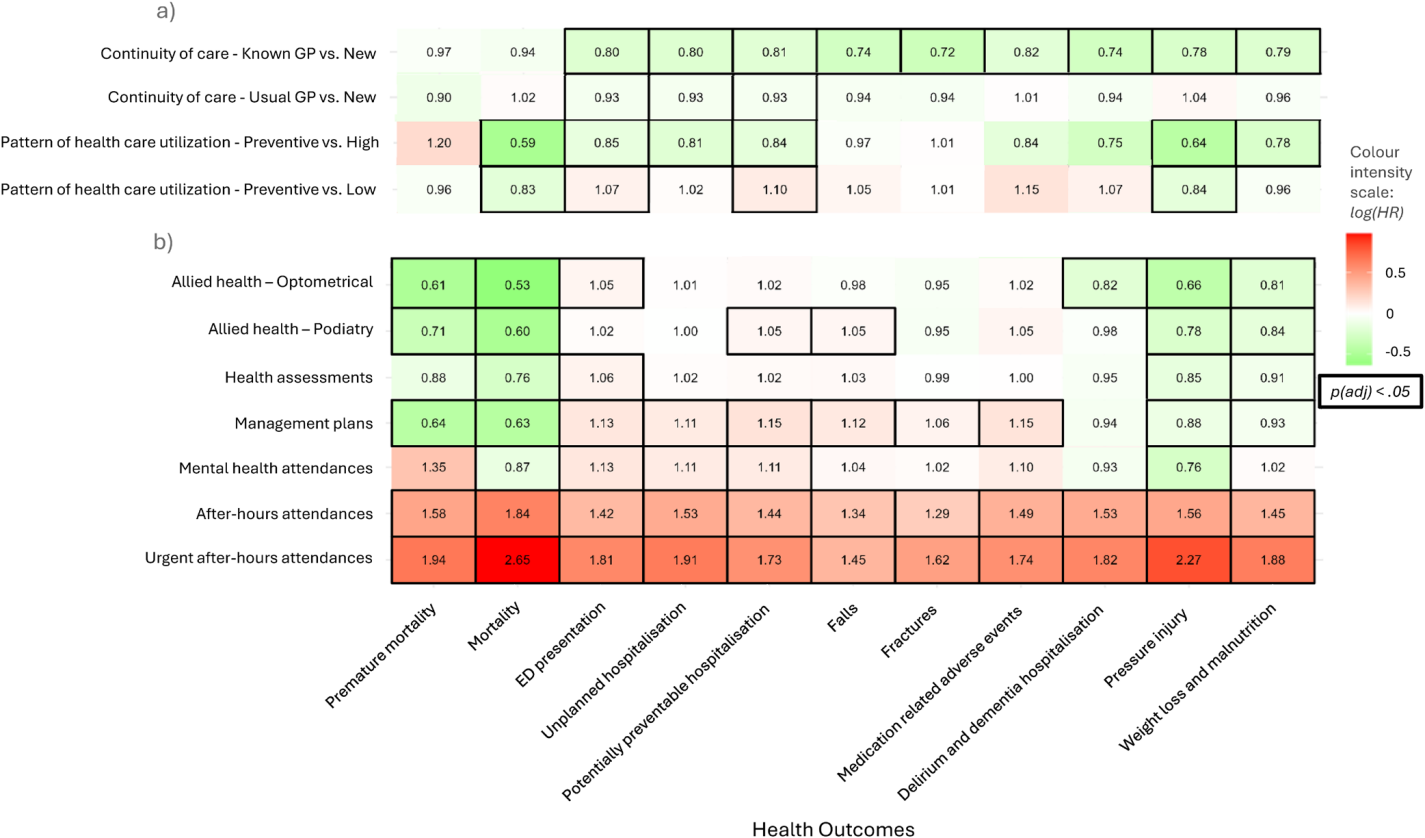
Associations between (a) derived primary care exposures (continuity of care and care patterns) and (b) individual primary care services with health outcomes. Hazard ratios (mortality outcomes) and sub‐distribution hazard ratios (other outcomes). Estimate < 1 (green): Lower risk of experiencing the outcome within 1 year. Estimate > = 1 (red): Higher risk of experiencing the outcome within 1 year. Cells with black outlines: Statistically significant estimates (after correction for multiple hypothesis testing). Abbreviations: ED = Emergency Department, GP = General Practitioner.

Compared to those who saw new GPs, those seeing a known GP had lower risks of ED presentations (sHR = 0.80, 95% CI 0.77–0.82), unplanned hospitalizations (sHR = 0.80, 95% CI 0.77–0.82), potentially preventable hospitalizations (sHR = 0.81, 95% CI 0.78–0.82), fall‐related hospitalizations (sHR = 0.74, 95% CI 0.70–0.79), fractures (sHR = 0.72, 95% CI 0.66–0.78), medication‐related hospitalization (sHR = 0.82, 95% CI 0.74–0.91), delirium/dementia‐related hospitalizations (sHR = 0.74, 95% CI 0.65–0.85), pressure injury‐related hospitalization (sHR = 0.78, 95% CI 0.71–0.85), and weight loss/malnutrition‐related hospitalizations (sHR = 0.79, 95% CI 0.73–0.86). Similarly, those who continued to see their usual GP had lower risks of ED presentations (sHR = 0.93, 95% CI 0.90–0.96), unplanned hospitalizations (sHR = 0.93, 95% CI 0.90–0.96), and potentially preventable hospitalizations (sHR = 0.93, 95% CI 0.89–0.98) by comparison to those who saw new GPs (Figure 2, Table S13).

### 3.3 Patterns of Primary Health Care

Three patterns (classes) of primary health care utilization for 54,489 older individuals were identified in the 12 months following study entry. These included high overall primary care utilization (7.3%, *n* = 4016 of study cohort) (Class 1: high long‐GP attendances, care management plans, allied health and after‐hours and urgent after‐hours GP attendances); low primary care utilization (30.2%, *n* = 16,452) (Class 2: low management plans, allied health, and after‐hours and urgent GP attendances); and high preventive primary care service pattern (62.4%, *n* = 34,021) (Class 3: high long‐GP attendances, care management plans and allied health, and low after‐hours and urgent GP attendances) (Table S2 and Figure S1).

High preventive primary care services was associated with lower risks of mortality (HR = 0.59, 95% CI 0.50–0.70), ED presentations (sHR = 0.85, 95% CI 0.80–0.92), unplanned hospitalizations (sHR = 0.81, 95% CI 0.75–0.87), potentially preventable hospitalizations (sHR = 0.84, 95% CI 0.76–0.93), pressure injury‐related hospitalization (sHR = 0.64, 95% CI 0.52–0.80), and weight loss and malnutrition‐related hospitalizations (sHR = 0.78, 95% CI 0.64–0.94) compared to high overall primary care service utilization. High preventive primary care services was associated with lower risks of mortality (HR = 0.83, 95% CI 0.76–0.91) and pressure injury‐related hospitalization (sHR = 0.84, 95% CI 0.74–0.94) but a higher risk of ED presentation (sHR = 1.07, 95% CI 1.03–1.11) and potentially preventable hospitalizations (sHR = 1.10, 95% CI 1.04–1.17) compared to low primary care service utilization (Figure 2, Table S13).

### 3.4 Individual Primary Health Care

Across individual services, preventive and monitoring‐oriented primary care services, such as optometric services, podiatry attendances, health assessments, and management plans, were associated with lower risks of mortality (ranging from 13 [HR = 0.87, 95% CI 0.77–0.98] to 47% [HR = 0.53, 95% CI 0.50–0.56]) and hospitalisations for pressure injuries and weight loss and malnutrition (ranging from 7% [sHR = 0.93, 95% CI 0.89–0.97] to 34% [sHR = 0.66, 95% CI 0.61–0.72]) (Figure 2, Table S13). The same services were associated with a 5%–13% higher risk of ED presentations (sHR = 1.05, 95% CI 1.03–1.09, to 1.13, 95% CI 1.08–1.18), except for podiatry attendances (Figure 2, Table S13). Management plans and podiatry attendances were additionally associated with 6%–15% higher risks of selected hospitalisations (sHR = 1.06, 95% CI 1.02–1.11, to 1.15, 95% CI 1.13–1.18), including potentially preventable hospitalisations, falls, fractures, and medication‐related adverse events for management plans (Figure 2, Table S13).

After‐hours and urgent after‐hours attendances were consistently associated with 29%–165% higher risks across all outcomes examined (sHR = 1.29, 95% CI 1.24–1.34 to 2.27, 95% CI 2.02–2.56, for hospitalization outcomes, and HR = 1.58, 95% CI 1.18–2.11, to 2.65, 95% CI 2.49–2.83, for mortality outcomes) (Figure 2, Table S13).

Detailed service‐specific estimates are presented in Figure 2 and Table S13.

### 3.5 Sensitivity Analysis

Findings were robust between the primary analysis and sensitivity analyses that examined additional covariates and level of home care supports (Figures S3–S5) and presence of dementia or diabetes (Figure S6). Delaying outcome ascertainment to 30 days post‐exposure period for individual health services resulted in modest attenuation of mortality outcomes for after‐hours and urgent after‐hours GP attendances (Figure S7).

## 4 Discussion

Accessible, effective and comprehensive primary care is central to support healthy aging of our older population. In this national population‐based cohort study of older people receiving home care, we have determined that the provision of primary care services focusing on prevention and multidisciplinary care management, and high continuity of care with their usual primary care physician were associated with lower mortality and improved health outcomes. This study provides the much‐needed evidence base to support policy and practice reforms for the primary care sector globally through quantification of the benefits across key health outcomes for the older population to support successful aging in place.

Continuity of care, a key component of high‐quality primary care, was the exposure examined associated with the most benefit in the current study. Prior work has determined that continuity of care is associated with lower risks of hospitalizations, mortality and increased use of guideline concordant care in the general population [27, 28, 29]. However, in the older population there is limited evidence, despite their complex care needs and high healthcare service utilization [9]. Only two studies were identified in a recent systematic review of home care recipients both from Canada focusing on primary care family physician continuity [30], with only one reporting a significant 4%–10% reduction in ED presentations and 4%–6% reduction in hospitalizations [31]. Further, the loss of a primary care physician among 350,000 older Medicare beneficiaries was associated with 21.5% fewer primary care visits, 9% more specialist visits, 18% increase in urgent care visits, 4.4% more ED visits and $189 increased Medicare spending per recipient in the following year [32]. In our study, 20% of the study population continued to see their usual GP during the exposure period and almost 50% saw a known GP, that were associated with a 11%–21% and 18%–28% reductions in hospitalizations (including ED presentations), respectively. The findings highlight the importance of ongoing primary care relationships to support older people with complex care needs, including the those living with dementia, to reduce hospitalizations and likely harms to improve overall patient wellbeing. While it is recognized that modern health care delivery has increased in complexity in the past few decades, our findings highlight the importance of having at least a known GP, which may be reflective of care being provided within a general practice potentially with transfer of information and management across providers [33]. While direct comparisons were not made, the apparent favorability of seeing a known GP compared to a usual GP should be interpreted in light of the fact that the two categories reflect different continuity constructs rather than an ordinal continuum. Given the magnitude of reduction in hospitalization observed in the current study, significant cost savings to the health sector are likely. Importantly, strategies investing in relational continuity of primary care, need to overcome previously identified barriers including workforce, equity of access in regional and remote areas and appropriate renumeration [30].

We also identified certain patterns of primary care utilization that offered the most benefit to the outcomes experienced by older people living in the community. Preventative, multidisciplinary primary care that includes high use of long GP attendances, team‐based care management plans and allied health services, moderate mental health services and low urgent‐ and after‐hours GP attendances, was associated with lower risk of hospitalizations (12%–32%) and mortality (18%–39%), compared to high overall primary care use that includes high urgent‐ and after‐hours GP attendances or those with low overall use of primary care. These findings align with prior evidence that preventative multidisciplinary primary care is associated with positive health outcomes in the settings of acute care, specific chronic conditions and palliative care [34, 35]. This is also supported by our recent review of models of care to support aging in place, identified person‐centred, multifactorial care models that address both the health and social care needs of older people in the community, which includes comprehensive assessment and care planning and delivered by a multidisciplinary clinical team had the most consistent evidence [36]. Further, these findings are critical to support expansion of the primary care workforce to their full scope of practice, which has been identified as a key barrier to provision of primary care by healthcare systems worldwide [37, 38]. A recent national primary care review in Australia, identified examples of multidisciplinary primary teams where care providers are working to their full scope of practice to deliver best practice primary care [39]. This also includes nurse practitioners which were used by < 5% of our older study population but have been shown to support continuity of care for the older population with chronic disease in both the primary and secondary healthcare settings that are associated with reduced hospitalizations or readmissions and patient satisfaction [40].

Associations observed for individual services were less uniform and should be interpreted in the context of potential alternative explanations, including healthy‐user effects, differential engagement with health services and residual confounding by illness severity, despite robust propensity score matching methods. Of the individual services, access to health assessments and care management plans were found to offer benefits for mortality and some hospitalizations (e.g., pressure injury and weight loss malnutrition), with negative effects observed for other hospitalization outcomes (e.g., ED presentations, unplanned hospitalizations). This discordant effect on hospitalization outcomes has previously been reported for older people waiting for long‐term care [41] and stroke management in community‐dwelling older individuals [42]. Having a health assessment or care plan likely includes discussion or documentation to facilitate patients recognition of signs and symptoms associated with acute exacerbations and disease worsening requiring immediate attention and likely ED or hospital presentation. While higher rates of ED presentations and unplanned hospitalisations may thus reflect appropriate escalation of care following earlier recognition of deterioration, this explanation is less applicable to event‐based outcomes such as falls, fractures, and medication‐related adverse events. For these outcomes, the observed associations may partly reflect residual confounding by indication, whereby service use is triggered by health deterioration. The associations of after‐hours and urgent after‐hours attendances with a 29%–165% increase in all outcomes observed, provides a potential signal to identify individuals at home who are likely to have poor health outcomes and require additional primary care and supports to mitigate risk. Further, a recent population‐based Danish study of over 4.5 million people reported a 21% increased likelihood of after‐hours attendances with poor general practice clinic continuity, again highlighting the importance of continuity of care to optimize health service use and improve health outcomes [43]. While optometry and podiatry were positively associated with reduced mortality and premature mortality, the associations lack plausible biological or clinical mechanisms, and are difficult to reconcile with simultaneous associations with higher risks of specific hospitalisations and emergency department presentations. These specific observations are likely reflective of differential health service engagement or unobserved confounding, rather than true protective effects.

## 5 Strengths and Limitations

This was a national population‐based examination of older people receiving long‐term care at home, with the findings likely generalisable to countries with similar primary care systems and social supports for older people, for example Canada, New Zealand or the United Kingdom. Our robust methodological and analytical approaches including our study exposures and outcomes that have high internal validity (derived from national cross‐sectoral data platform) [16], examination of primary care patterns of use and continuity reflective of real‐world health care utilization and sophisticated confounding adjustments, ensured a comprehensive evaluation of these services to generate robust real‐world evidence.

We examined government‐subsidized (MBS) primary care; some services, especially allied health, may be accessed privately or via hospital/state programs and are not fully captured, likely underestimating use and biasing associations toward the null [44]. Despite comprehensive propensity‐score weighting/matching, residual confounding remains possible. For continuity of care, patterns of care, and urgent‐ and after‐hours GP attendances, estimands reflect the average effect of the treatment on the treated (ATT); other contrasts estimate average treatment effects (ATE). Selection bias (i.e., access to services is related to reasons for outcome) was plausibly present in the case of urgent and after‐hours GP attendances but after‐hours services are simultaneously potentially reflective of suboptimal regular primary care. A healthy‐user effect may partly explain favorable associations for optometry and podiatry. Continuity and pattern analyses were limited to those who survived six and twelve months, but with 10% dying within a year, generalisability is only limited slightly. Finally, Aboriginal or Torres Strait Islander individuals, who represent ~3.4% of new users of long‐term home care in Australia each year [45], were not included due to required governance and leadership by these individuals to undertake analysis of their data, limiting generalisability [45].

## 6 Conclusions

This national study provides observational evidence to support the use of primary care that focuses on prevention and multidisciplinary disease management and care planning, and continuity of care to improve older people's health and outcomes. With increased pressure on health care systems worldwide to meet the growing demands of the aging population, our findings highlight the need for clear policy and practice change from models of care that are episodic, disease‐focused and reactive. Continuity of care with a primary care physician, together with multidisciplinary primary care team that focuses on individual care needs and priorities is needed to support our older populations to not only successfully age in place, but to age well.

## Author Contributions

Conceptualization: G.E.C., M.I., M.C., S.L.W., and G.H. Data curation: G.E.C., M.I., and J.S. Formal analysis: J.S. and G.E.C. Funding acquisition: All. Investigation: All. Methodology: G.E.C., M.I., and J.S. Project administration: G.E.C. and M.I. Resources: All. Writing original draft: G.E.C. and J.S. Writing review and editing: All. M.v.T. is a consumer representative who provided lived experience throughout all parts of the study.

## Funding

This work is supported by funding from an Australian Government Medical Research Future Fund Primary Health Care Research Grant (MRFF1200056). GEC is supported by a National Health and Medical Research Council (NHMRC) Investigator Grant (GNT2026400). JKS is supported by an NHMRC Investigator Grant (GNT2016277). MCa is supported by a MRFF/NHMRC Investigator Grant (GNT1194084). MCI is supported by a NHMRC Investigator Grant (GNT119378).

## Ethics Statement

This study was approved by the University of South Australia (Ref: 200489), Australian Institute of Health and Welfare (EO2022/4/1376), South Australian Department for Health & Wellbeing (HREC/18/SAH/90), New South Wales Population & Health Services (2019/ETH12028), and Department of Defense and Veterans’ Affairs (EO2022/4/1376) Human Research Ethics committees. The study followed the Strengthening the Reporting of Observational Studies in Epidemiology (STROBE) reporting guideline.

## Conflicts of Interest

M.I. has received grants paid to her institution from the Australian Government Medical Research Institute, National Health and Medical Research Council (NHMRC), and Hospital Research Foundation. M.I. has also received sitting fees for membership in Australian Government Expert Advisory Committees and support for traveling expenses to meetings and conferences by the Australian Government and Australian Healthcare and Hospitals Association. G.E.C. has received grants (paid to the institution) from the Australian Government Medical Research Institute and NHMRC. G.E.C. has also received sitting fees for membership in Australian Government Expert Advisory Committees. J.K.S. has received grants (paid to the institution) from the Australian Government Medical Research Institute, NHMRC, Australian Association of Consultant Pharmacy and University of South Australia. J.K.S. has also received support for traveling expenses to meetings and conferences by the University of Sydney, Informa Australia and Australian Association of Consultation Pharmacy and payments for leadership position at Australian Medic Alert Foundation and Southern Cross Care. M.v.T. has received sitting fees for membership of the Registry of Senior Australians Consumer and Community Advisory Committee. H.W., who is a registered specialist G.P., has a salaried role with the Silverchain Group (aged care provider) and Australian Commission on Safety and Quality in Healthcare.

## Supporting information


**Table S1:** Health Care Exposures of Interest: Descriptions and Codes.
**Table S2:** Summary of Patterns of Health Care Service Utilization Identified from Latent Class Analysis.
**Table S3:** Outcomes of Interest: Data Source, Coding, and Definitions.
**Table S4:** Baseline Characteristics of Study Cohort by GP After‐Hours Attendances Before and After Weighting.
**Table S5:** Baseline Characteristics of Study Cohort by Urgent After‐Hours GP Attendances Before and After Weighting.
**Table S6:** Baseline Characteristics of Study Cohort by Health Assessment Utilization Before and After Weighting.
**Table S7:** Baseline Characteristics of Study Cohort by Management Plan Utilization Before and After Weighting.
**Table S8:** Baseline Characteristics of Study Cohort by Podiatry Utilization Before and After Weighting.
**Table S9:** Baseline Characteristics of Study Cohort by Optometrical Service Utilization Before and After Weighting.
**Table S10:** Baseline Characteristics of Study Cohort by GP Mental Health Services Utilization Before and After Matching.
**Table S11:** Baseline Characteristics of Study Cohort by Pattern of Service Utilization Exposures Before and After Matching.
**Table S12:** Crude Health Outcome Event Rate and Cumulative Incidence within One Year by Patterns of Health Care Service Utilization and Specific Primary Health Care Services Exposures.
**Table S13:** Associations Between Primary Health Care Services and Health Outcomes, Hazard Ratios and Sub‐distribution Hazard Ratios and 95% Confidence Intervals.
**Figure S1:** Patterns of Health Care Service Utilization by Latent Class Analysis—Identified Classes.
**Figure S2:** Flow chart of the sensitivity analysis.
**Figure S3:** Associations Between Specific Primary Care Services, Continuity of Care and Health Outcomes in Sensitivity Analysis by Additional Covariates and All Care Levels. Hazard ratios (mortality outcomes) and sub‐distribution hazard ratios (other outcomes).
**Figure S4:** Associations Between Specific Primary Care Services, Continuity of Care and Health Outcomes in the Sensitivity Analysis by Home Care Levels 1 and 2. Hazard ratios (mortality outcomes) and sub‐distribution hazard ratios (other outcomes).
**Figure S5:** Associations Between Specific Primary Care Services, Continuity of Care and Health Outcomes in the Sensitivity Analysis by Home Care Levels 3 and 4. Hazard ratios (mortality outcomes) and sub‐distribution hazard ratios (other outcomes).
**Figure S6:** Associations Between GP Management Plans and Health Outcomes in the Sensitivity Analysis Stratifying by Dementia and Diabetes. Hazard ratios (mortality outcomes) and sub‐distribution hazard ratios (other outcomes).
**Figure S7:** Associations Between Specific Primary Care Services and Health Outcomes in the Sensitivity Analysis Delaying Outcome Ascertainment to 30 days Post‐exposure Period. Hazard ratios (mortality outcomes) and sub‐distribution hazard ratios (other outcomes).

## Data Availability

The data from this study is not available for sharing by the researchers due to ethical restrictions and approvals required to access and link the study data. The datasets can be recreated by seeking approval for access from original data custodians for all datasets included in the existing data source and data integrating authorities to link it.
