# Supplementary material for: Primary Health Care Services and Continuity of Care Are Associated With Better Health Outcomes in the Older Population
Source: J Am Geriatr Soc. 2026 Apr 28;74(7):1950–63. doi: 10.1111/jgs.70465 (PMC13418550; doi:10.1111/jgs.70465)
Supplement: Supplementary file 1 — Table S1: Health Care Exposures of Interest: Descriptions and Codes. Table S2: Summary of Patterns of Health Care Service Utilization Identified from Latent Class Analysis. Table S3: Outcomes of Interest: Data Source, Coding, and Definitions. Table S4: Baseline Characteristics of Study Cohort by GP After‐Hours Attendances Before and After Weighting. Table S5: Baseline Characteristics of Study Cohort by Urgent After‐Hours GP Attendances Before and After Weighting. Table S6: Baseline Characteristics of Study Cohort by Health Assessment Utilization Before and After Weighting. Table S7: Baseline Characteristics of Study Cohort by Management Plan Utilization Before and After Weighting. Table S8: Baseline Characteristics of Study Cohort by Podiatry Utilization Before and After Weighting. Table S9: Baseline Characteristics of Study Cohort by Optometrical Service Utilization Before and After Weighting. Table S10: Baseline Characteristics of Study Cohort by GP Mental Health Services Utilization Before and After Matching. Table S11: Baseline Characteristics of Study Cohort by Pattern of Service Utilization Exposures Before and After Matching. Table S12: Crude Health Outcome Event Rate and Cumulative Incidence within One Year by Patterns of Health Care Service Utilization and Specific Primary Health Care Services Exposures. Table S13: Associations Between Primary Health Care Services and Health Outcomes, Hazard Ratios and Sub‐distribution Hazard Ratios and 95% Confidence Intervals. Figure S1: Patterns of Health Care Service Utilization by Latent Class Analysis—Identified Classes. Figure S2: Flow chart of the sensitivity analysis. Figure S3: Associations Between Specific Primary Care Services, Continuity of Care and Health Outcomes in Sensitivity Analysis by Additional Covariates and All Care Levels. Hazard ratios (mortality outcomes) and sub‐distribution hazard ratios (other outcomes). Figure S4: Associations Between Specific Primary Care Services, Continuity of Care and Healt [file JGS-74-1950-s001.pdf]

Primary health care services and continuity of care are associated with better health outcomes in the older population

Supplementary Materials

Supplementary Methods 3

Supplementary Table 1. Health Care Exposures of Interest: Descriptions and Codes 7

Supplementary Table 2. Summary of Patterns of Health Care Service Utilization Identified from Latent Class Analysis 10

Supplementary Table 3. Outcomes of Interest: Data Source, Coding, and Definitions 11

Supplementary Table 4. Baseline Characteristics of Study Cohort by GP After-Hours Attendances Before and After Weighting 14

Supplementary Table 5. Baseline Characteristics of Study Cohort by Urgent After-Hours GP Attendances Before and After Weighting 16

Supplementary Table 6. Baseline Characteristics of Study Cohort by Health Assessment Utilization Before and After Weighting 18

Supplementary Table 7. Baseline Characteristics of Study Cohort by Management Plan Utilization Before and After Weighting 20

Supplementary Table 8. Baseline Characteristics of Study Cohort by Podiatry Utilization Before and After Weighting 22

Supplementary Table 9. Baseline Characteristics of Study Cohort by Optometrical Service Utilization Before and After Weighting 24

Supplementary Table 10. Baseline Characteristics of Study Cohort by GP Mental Health Services Utilization Before and After Matching 26

Supplementary Table 11. Baseline Characteristics of Study Cohort by Pattern of Service Utilization Exposures Before and After Matching 28

Supplementary Table 12. Crude Health Outcome Event Rate and Cumulative Incidence within One Year by Patterns of Health Care Service Utilization and Specific Primary Health Care Services Exposures 31

Supplementary Table 13. Associations Between Primary Health Care Services and Health Outcomes, Hazard Ratios and Sub-distribution Hazard Ratios and 95% Confidence Intervals 40

Supplementary Figure 1. Patterns of Health Care Service Utilization by Latent Class Analysis - Identified Classes 41

Supplementary Figure 2. Flow chart of the sensitivity analysis. 42

Supplementary Figure 3. Associations Between Specific Primary Care Services, Continuity of Care and Health Outcomes in Sensitivity Analysis by Additional Covariates and All Care Levels. Hazard ratios (mortality outcomes) and sub-distribution hazard ratios (other outcomes). 43

Supplementary Figure 4. Associations Between Specific Primary Care Services, Continuity of Care and Health Outcomes in the Sensitivity Analysis by Home Care Levels 1 and 2. Hazard ratios (mortality outcomes) and sub-distribution hazard ratios (other outcomes). 44

Supplementary Figure 5. Associations Between Specific Primary Care Services, Continuity of Care and Health Outcomes in the Sensitivity Analysis by Home Care Levels 3 and 4. Hazard ratios (mortality outcomes) and sub-distribution hazard ratios (other outcomes). 45

Supplementary Figure 6. Associations Between GP Management Plans and Health Outcomes in the Sensitivity Analysis Stratifying by Dementia and Diabetes. Hazard ratios (mortality outcomes) and sub-distribution hazard ratios (other outcomes). 46

Supplementary Figure 7. Associations Between Specific Primary Care Services and Health Outcomes in the Sensitivity Analysis Delaying Outcome Ascertainment to 30 days Post-exposure Period. Hazard ratios (mortality outcomes) and sub-distribution hazard ratios (other outcomes). 47

Supplementary Methods

Study setting, design, and data source

The ROSA datasets used in this study included: (1) national long-term care eligibility assessments for services; (2) national care records; (3) national pharmaceutical dispensing records (Pharmaceutical Benefits Scheme [PBS]); (4) national subsidised health service records (Medicare Benefits Schedule [MBS]); state-based (5) hospitalization and (6) emergency department (ED) presentations for four out of seven states and major territories (87% of the national cohort); and (7) death records (National Death Index [NDI]).

Exposures of interest

Primary care services included: general attendances by general practitioners (GPs) (or medical practitioners) in Australia (analogous to primary care physicians), after-hours GP attendances, or urgent after-hours GP attendances; nurse practitioner attendances; GP health assessments, GP management plans, and attendances associated with practice incentive programs. Allied health services examined included: optometrical services, comprehensive medication reviews provided by pharmacists in collaboration with GPs, and allied health services delivered as part of a management plan. Podiatry services constituted 86% of the allied health services accessed as part of a management plan, so only podiatry services were examined as part of this exposure. Primary care mental health care services were examined as one category and included psychological therapy, focussed psychological strategies, and GP mental health attendances. Services infrequently accessed (<5%) were not considered further.

Index date assignment

Controls were matched into one of 48 unique subgroups of exposed individuals based on four criteria: age group (65-74, 75-84, 85+), sex, location remoteness (major cities vs other), and number of unique health conditions (0-1, 2-3, 4+). Within each subgroup and for each exposure, the median time from first home care service entry date to first exposure among the exposed was assigned as the index date for unexposed individuals. Unexposed individuals were only included in further analyses if they were alive and in home care at the assigned index date and the index date was before study end (retention of 72-93% of the total cohort depending on exposure).

Patterns of primary health care utilization

The latent class analysis included all primary health care services accessed by at least 5% of all individuals in the year after entry. For further discrimination, GP attendances and after-hours attendances were split into long and short duration. The latent class analysis did not include GP health assessments since access to this item is restricted to those aged 75 or older and would not be inclusive of the entire study cohort. Shown in Supplementary Table 2 and Supplementary Figure 1 are the summary of patterns of primary health care service utilization. The decision between the final solutions was based on entropy, elbow plots of fit statistics (Akaike information criterion, AIC, and Bayesian information criterion, BIC), and the interpretability of classes.

Covariates

Individual factors examined as confounders included: age, sex, culturally and linguistically diverse status, partner status, specific health conditions (dementia, pressure injuries, incontinence, cancer, pain, chronic respiratory disease, congestive heart failure, depression, diabetes, ischemic heart disease hypertension, osteoporosis, falls and fractures), total number of additional health conditions using a comorbidity index (excluding the specific conditions examined above), Socio-Economic Indexes for Areas (SEIFA) Indices of Relative Socioeconomic Disadvantage, and Education and Occupation (based on individuals postcode), home care level at commencement of care supports, maximal approved home care level from assessment, approvals for long-term care facility entry or

respite care, and wait time from approval to care (logarithmized). Health conditions were ascertained from aged care eligibility assessment data and Rx-Risk-V pharmaceutical based comorbidity index.26 Long-term home care provider characteristics examined included location (state, geographical remoteness27) and ownership (not for profit, for profit, or government).

Health care utilization characteristics one year prior to home care entry examined included: number of hospitalizations (total, unplanned, and potentially preventable, capped at 10), number of ED presentations (overall and potentially preventable, capped at 10), cumulative length of hospital stays (total, unplanned, and potentially preventable, logarithmized), 30-day history of any hospitalizations and ED presentations (capped at 10), and history of the service examined as exposure.

Propensity score methods

Fine stratification (FS) weighting or propensity score matching was employed to create comparable samples of people for each exposure. Propensity scores were obtained by regressing the binary exposure variables on individual, provider, and health care utilization covariates using logistic regression models. Due to the small number of missing data (n=1234/120522, 1.0%) a complete case analysis was conducted (Figure 1). The analysis cohort for GP health assessments was restricted to individuals aged 75 or older to reflect service eligibility restrictions (82% of total cohort). For six of the seven exposures examined, accessed by >5% of people, the overlaps in propensity score kernel density distributions of exposed and unexposed were sufficient to estimate FS weights. For three of the six FS exposures, the discrimination of the propensity score models was sufficient to estimate Average Treatment Effects (ATE). Average Treatment Effect on the Treated (ATT) weights were calculated for the other three. For mental health attendances, FS could not be estimated, and people were matched based on the logit of the propensity score with a 3:1 ratio using a nearest-neighbour approach without replacement and using callipers of width

equal to 0.2 of the standard deviation of the propensity score logit, targeting the ATT. The same method was employed to match people in the continuity of care and care patterns exposures. The reference groups for matching and analysis were people in the continuity of care category reflecting no continuity ("New GP") and the care pattern class reflecting preventive service use (class 3). Baseline characteristics of each exposure group before and after matching and covariance balance (standardised mean differences) are in **Table 1** using continuity of care as an example and **Supplementary Tables 4-11** for other exposures.

Sensitivity analysis

Shown in **Supplementary Figure 2** is the flow chart for the 109819 individuals included in the sensitivity analysis that included additional covariates from the eligibility assessment including: eleven covariates assessing functional impairment (related to getting places, shopping, meal preparation, housework, taking medicines, handling money, walking, bathing, dressing, eating, and transferring), one complexity (memory confusion); and two psychological impairment (short-term memory problems, insomnia) covariates. Prior to the matching or weighting and the outcome models, the cohort was additionally stratified by home care level into three groups (all levels, home care levels 1 and 2, and home care levels 3 and 4). All other parts of the analysis remained unchanged.

Supplementary Table 1. Health Care Exposures of Interest: Descriptions and Codes

Health Care Exposures	MBS Groups	MBS Group Description	MBS items
**General Attendances**			
**GP/Medical practitioner attendances**	A01	GP attendances	3, 4, 23, 24, 36, 37, 44, 47
	A02	Non-referred attendance to medical practitioner	52, 53, 54 ,57, 58, 59, 60, 65
	A35[a]	Medical services at long term care facility	20, 35, 43, 51, 92, 93, 95, 96, 183, 188, 202, 212, 90020, 90035, 90043, 90051, 90092, 90093, 90095, 90096, 90183, 90188, 90202, 90212
**After-hours attendances**	A22	GP after-hours attendance	5000, 5003, 5010, 5020, 5023, 5028, 5040, 5043, 5049, 5060, 5063, 5067
	A23	Non-referred after-hours attendance with medical practitioners	5200, 5203, 5207, 5208, 5220, 5223, 5227, 5228, 5260, 5263, 5265, 5267
**Urgent attendance after-hours**	A11	Urgent GP/Medical practitioner attendance after-hours	585, 588, 591, 594, 597, 598, 599, 600
**Nurse practitioner attendances**	M14	Nurse practitioners	82200, 82205, 82210, 82215, 82220, 82221, 82222, 82223, 82224, 82225
**Health Assessments / Management Plans**			
**Health assessments**	A14	GP/Medical practitioner health assessments	224, 225, 226, 227, 701, 703, 705, 707
**Management plans**	A15	GP/Medical practitioner management plan attendances/team care arrangements and multidisciplinary care plans	229, 230, 231, 232, 233, 235, 236, 237, 238, 239, 240, 243, 244, 721, 723, 729, 731, 732, 735, 739, 743, 747, 750, 758, 871, 872
**GP/ Medical practitioner attendance associated with practice incentives program payment**	A18/19	GP/Medical practitioner attendance associated with practice incentives program payment	251, 252, 253, 254, 255, 256, 257, 259, 260, 261, 262, 263, 265, 266, 268, 269, 270, 271, 2497, 2501, 2503, 2504, 2506, 2507, 2509, 2517, 2518, 2521, 2522, 2525, 2526, 2546, 2547, 2552, 2553, 2558, 2559, 2598, 2600, 2603, 2606, 2610, 2613, 2616, 2620, 2622, 2624, 2631, 2633, 2635, 2664, 2666, 2668, 2673, 2675, 2677
**Allied Health Services**			
**Allied health service part of CDMP**	M03	Allied health component of chronic disease management plan (GP management plan)	10951, 10952, 10953, 10954, 10956, 10958, 10960, 10962, 10964, 10966, 10968, 10970
		Podiatry services	10962
**Optometrical services**	A10	Optometrical services	10905, 10907, 10911, 10912, 10913, 10915, 10916, 10918, 10922, 10923, 10924, 10925, 10926, 10927, 10928, 10929, 10930, 10931, 10932, 10933, 10940, 10941, 10942, 10943, 10944, 10945, 10946, 10947, 10948
**Comprehensive medication review**	A17	Domiciliary and residential[a] medication management reviews	245, 249, 900, 903

Health Care Exposures	MBS Groups	MBS Group Description	MBS items
**Primary Care Mental Health Services**			
**Psychological therapy**	M06	Psychological therapy services	80000, 80001, 80005, 80010, 80011, 80015, 80020, 80021
**Focussed psychological strategies**	M07	Focussed psychological strategies	941, 942, 2721, 2723, 2725, 2727, 80100, 80101, 80105, 80110, 80111, 80115, 80120, 80121, 80125, 80126, 80130, 80135, 80136, 80140, 80145, 80146, 80150, 80151, 80155, 80160, 80161, 80165, 80170, 80171
**GP mental health**	A20	GP mental health treatment	272, 276, 277, 279, 281, 282, 2700, 2701, 2702, 2712, 2713, 2715, 2717, 2719
**Derived Exposures**			
**Continuity of Care - Primary Care Services Included**			
**GP/Medical practitioner attendances**	A01	GP attendance	3, 4, 23, 24, 36, 37, 44, 47
	A02	Non-referred attendance to medical practitioner	52, 53, 54 ,57, 58, 59, 60, 65
	A35[a]	Medical services at long-term care facility	20, 35, 43, 51, 92, 93, 95, 96, 183, 188, 202, 212, 90020, 90035, 90043, 90051, 90092, 90093, 90095, 90096, 90183, 90188, 90202, 90212
**GP/Medical practitioner after-hours attendances**	A22<br>A23	GP after-hours attendance<br>Non-referred after-hours attendance with medical practitioners	5000, 5003, 5010, 5020, 5023, 5028, 5040, 5043, 5049, 5060, 5063, 5067<br>5200, 5203, 5207, 5208, 5220, 5223, 5227, 5228, 5260, 5263, 5265, 5267
**Urgent GP/Medical practitioner attendance after-hours**	A11	Urgent GP after-hours attendance	585, 588, 591, 594, 597, 598, 599, 600
**Health assessments**	A14	GP/Medical practitioner health assessments	224, 225, 226, 227, 701, 703, 705, 707
**Management plans**	A15	GP management plan attendances/team care arrangements and multidisciplinary care plans	229, 230, 231, 232, 233, 235, 236, 237, 238, 239, 240, 243, 244, 721, 723, 729, 731, 732, 735, 739, 743, 747, 750, 758, 871, 872
**Patterns of Health Care Service Utilization Primary Care Services Included (≥5% utilization)**			
**GP/Medical practitioner attendances – short**	A01	GP attendance	3, 4, 90020, 23, 24, 90035
	A02	Non-referred attendance to medical practitioner	52, 53, 58, 59
	A35[a]	Medical services at long-term care facility	20, 35, 92, 93, 90092, 90093, 183, 188, 90183, 90188

Health Care Exposures	MBS Groups	MBS Group Description	MBS items
**GP/Medical practitioner attendances – long**	A01	GP attendance	36, 37, 43, 90043, 44, 47
	A02	Non-referred attendance to medical practitioner	54, 57, 60, 65
	A35[a]	Medical services at long-term care facility	43, 90043, 51, 90051, 95, 96, 90095, 90096, 202, 212, 90202, 90212
**GP/Medical practitioner after-hours attendances – short**	A22	GP after-hours attendance	5000, 5003, 5010, 5020, 5023, 5028
	A23	Non-referred after-hours attendance with medical practitioners	5200, 5203, 5220, 5223, 5260, 5263
**GP/Medical practitioner after-hours attendances – long**	A22	GP after-hours attendance	5040, 5043, 5049, 5060, 5063, 5067
	A23	Non-referred after-hours attendance with medical practitioners	5207, 5208, 5227, 5228, 5265, 5267
**Urgent GP/Medical practitioner attendance after-hours**	A11	Urgent GP attendance after-hours	585, 588, 591, 594, 597, 598, 599, 600
**Management plans**	A15	GP/Medical practitioner management plan attendances/team care arrangements and multidisciplinary care plans	229, 230, 231, 232, 233, 235, 236, 237, 238, 239, 240, 243, 244, 721, 723, 729, 731, 732, 735, 739, 743, 747, 750, 758, 871, 872
**Allied health service part of CDMP**	M03	Allied health (podiatry only) component of chronic disease management plan (GP management plan)	10962
**Optometrical services**	A10	Optometrical services	10905, 10907, 10911, 10912, 10913, 10915, 10916, 10918, 10922, 10923, 10924, 10925, 10926, 10927, 10928, 10929, 10930, 10931, 10932, 10933, 10940, 10941, 10942, 10943, 10944, 10945, 10946, 10947, 10948
**Primary care mental health services**	M06	Psychological therapy services	80000, 80001, 80005, 80010, 80011, 80015, 80020, 80021
	M07	Focussed psychological strategies	941, 942, 2721, 2723, 2725, 2727, 80100, 80101, 80105, 80110, 80111, 80115, 80120, 80121, 80125, 80126, 80130, 80135, 80136, 80140, 80145, 80146, 80150, 80151, 80155, 80160, 80161, 80165, 80170, 80171
	A20	GP mental health treatment	272, 276, 277, 279, 281, 282, 2700, 2701, 2702, 2712, 2713, 2715, 2717, 2719

Abbreviations: MBS=Medicare Benefits Schedule. GP=General Practitioner, CDMP=Chronic Disease Management Plan.
[a]Item codes only available to n=629 individuals who received short-term respite care during the study period.

Supplementary Table 2. Summary of Patterns of Health Care Service Utilization Identified from Latent Class Analysis

Class	Total cohort	Management plans, allied health services	Primary care mental health services	After-hours and urgent attendances after-hours
1 – High overall primary care use	4016	High	High	High
2 – Low overall primary care use	16452	Low	Low	Low
3 – High preventive primary care service use	34021	High	Medium	Low

Supplementary Table 3. Outcomes of Interest: Data Source, Coding, and Definitions

Outcome	Data Source(s) within ROSA	Codes	Notes
Premature mortality	NDI	ICD-10-AM: External and potentially avoidable causes of death: V01-V99, W00-W99, X00-X84, X85-Y09, Y10-Y34, Y40-Y84, Y85-Y98	
Mortality	NDI	ICD-10-AM: Any	
Emergency department presentation	NSW EDDC, VEMD, QLD EDC, SA NAEC	ICD-10-AM: Any	Non-scheduled presentations only.
Unplanned hospitalizations	NSW APDC, VAED, QHAPDC, SA APC	ICD-10-AM: Any	Non-elective hospitalizations only.
Potentially preventable hospitalizations	NSW APDC, VAED, QHAPDC, SA APC	ICD-10-AM:<br>*Acute*: J15.3, J15.4, J15.7, J16.0, L02*, L03*, L04*, L08*, L88*, L98.0, L98.3, G40*, G41*, R56*, K028, K03*, K04*, K05*, K06*, K08*, K12*, K13*, K09.8, K09.9, K14.0, H66*, J02*, J03*, J06*, J31.2, I70.24, E09.52, R02*, K25.0, K25.1, K25.2, K25.4, K25.5, K25.6, K26.0, K26.1, K26.2, K26.4, K26.5, K26.6, K27.0, K27.1, K27.2, K27.4, K27.5, K27.6, K28.0, K28.1, K28.2, K28.4, K28.5, K28.6, N70*, N73*, N74*, N10-12*, N13.6, N15.1, N15.9, N28.9, N39.0, N39.9.<br>*Chronic:* J45*, J46*, J47*, I50*, I11.0, J81*, J20*, J41*, J42*, J43*, J44*, J20*, J47*, E10-E11*, E13-E14*, I20*, I24.0, I24.8, I24.9, D50.1, D50.8, D50.9, I10*, I11.9, E40*, E41*, E42*, E43*, E55.0, I00-I02*, I05-I09*<br>*Vaccine-related*: J10*, J11*, J13*, J14*, A08.0 A35-A37*, A80*, B01*, B05*, B06*, B16.1, B16.9, B18.0, B18.1, B26*, G00.0	Any.
Falls	NDI, NSW EDDC, SA NAEC, VEMD, QLD EDC, NSW APDC, VAED, QHAPDC, SA APC	ICD-10-AM: W00*, W01*, W03*-W19*, R29.6	Included only cases with 'external causes of injury' for inpatient admissions, and diagnosis for emergency department presentations (or 'injury cause' for VEMD only).
Fractures	NSW EDDC, SA NAEC, VEMD, QLD EDC, NSW APDC, VAED, QHAPDC, SA APC, MBS	*ICD-10-AM:* S01.81, S02*, S12*, S21.81, S22*, S31.81, S32*, S41.81, S42*, S51.81, S52*, S61.81, S62*, S71.81, S72*, S81.81, S82*, S91.81, S92*, T02*, T08*, T10*, T12*, T14.2*<br>*MBS items:* 39606, 39609, 39612, 39615, 45975, 45978, 45981, 45984, 45987, 45990, 45993, 45996, 46442, 47024, 47027, 47300, 47301, 47303, 47306, 47307, 47309, 47310, 47312, 47313, 47315, 47316, 47318, 47321, 47324, 47327, 47330, 47333, 47336, 47339, 47342, 47345, 47348, 47351, 47354, 47357, 47360, 47361, 47362, 47363, 47364, 47366, 47367,	Primary diagnosis or MBS procedure.

		47369, 47370, 47372, 47373, 47375, 47378, 47381, 47384, 47385, 47386, 47387, 47390, 47393, 47396, 47399, 47402, 47405, 47408, 47411, 47414, 47417, 47420, 47423, 47426, 47429, 47432, 47435, 47438, 47441, 47444, 47447, 47450, 47451, 47453, 47456, 47459, 47462, 47465, 47466, 47467, 47468, 47471, 47474, 47477, 47480, 47483, 47486, 47489, 47492, 47495, 47498, 47501, 47504, 47507, 47510, 47516, 47519, 47522, 47525, 47528, 47531, 47534, 47537, 47543, 47546, 47549, 47552, 47555, 47558, 47561, 47564, 47565, 47566, 47567, 47570, 47573, 47576, 47579, 47582, 47585, 47588, 47591, 47594, 47597, 47600, 47603, 47606, 47609, 47612, 47615, 47618, 47621, 47624, 47627, 47630, 47633, 47636, 47639, 47642, 47645, 47648, 47651, 47654, 47657, 47663, 47666, 47672, 47678, 47681, 47684, 47687, 47690, 47693, 47696, 47699, 47702, 47703, 47735, 47738, 47741, 47753, 47756, 47762, 47765, 47768, 47771, 47774, 47777, 47780, 47783, 47786, 47789, 49336, 53400, 53403, 53406, 53409, 53410, 53411, 53412, 53413, 53414, 53415, 53416, 53418, 53419, 53422, 53423, 53424, 53425, 53427, 53429, 53439, 53458, 53459, 53460	
Medication-related adverse events	NSW EDDC, SA NAEC, VEMD, QLD EDC, NSW APDC, VAED, QHAPDC, SA APC	*ICD-10-AM Primary Diagnosis:* A04.7*, A80.0, D52.1, D59.0, D59.2, D61.1, D64.2, D68.3, D69.0, D69.5, E03.2, E06.4, E15, E16.0, E23.1, E24.2, E27.3, E66.10, F11*, F13*, F15*, F19*, F55*, G21.0, G21.1, G21.2, G24.0, G25.1, G25.4, G25.6, G44.4, G62.0, G72.0, H26.3, H40.6, H91.0, I42.7, I95.2, J70.2, J70.3, J70.4, K52.1, K71.0, K71.1, K71.2, K71.6, K71.9, K85.3, L10.5, L23.3, L24.4, L25.1, L27.0, L27.1, L27.8, L27.9, L43.2, L51.2, L56.0, L56.1, L64.0, M10.2*, M32.0, M34.2, M80.4*, M81.4*, M83.5, M87.1, N14.0, N14.1, N14.2, N14.3, N14.4, R50.2, T36*, T37*- T50*, T78.2, T78.3, T78.4, T78.8, T78.9, T80.1, T80.2, T80.3, T80.4, T80.5, T80.6, T80.8, T80.9, T88.3, T88.6, T88.7, T96<br><br>*ICD-10-AM External Causes*: X40*, X41*, X42*, X43*, X44*, Y10,Y11*, Y12*,Y13*, Y14*, Y40-Y59, Y63.6, Y88.0	Primary diagnosis or external cause diagnosis.
Delirium and/or dementia hospitalizations	NSW EDDC, SA NAEC, VEMD, QLD EDC, NSW APDC, VAED, QHAPDC, SA APC	ICD-10-AM: R41, R41.0, R41.8, G30*, G31.3, F00*, F01*, F02*, F03*, F05*	Primary diagnosis.
Pressure injury	NSW EDDC, SA NAEC, VEMD, QLD EDC, NSW APDC, VAED, QHAPDC, SA APC	ICD-10-AM: L89.0*, L89.1*, L89.2*, L89.3*, L89.4*, L89.5*, L89.9*	Any.
Weight loss and malnutrition	NSW EDDC, SA NAEC, VEMD, QLD EDC, NSW APDC, VAED, QHAPDC, SA APC	ICD-10-AM: E43, E44.0, E44.1, E46, E63.9, R63.4, R63.6, R64, Z68.1	Any.

Abbreviations: ICD-10-AM = International Statistical Classification of Diseases and Related Health Problems, Tenth Revision, Australian Modification. NDI = National Death Index. NSW EDDC = New South Wales Emergency Department Data Collection. VEMD = Victorian Emergency Minimum Dataset. QLD EDC = Queensland Emergency Department

Collection (inclusive of Emergency Department Information System and FirstNet). SA NAEC = South Australia Non-Admitted Emergency Care. NSW APDC = New South Wales Admitted Patient Data Collection. VAED = Victorian Admitted Episodes Dataset. QHAPDC = Queensland Hospital Admitted Patient Data Collection. SA APC = South Australia Admitted Patient Care. MBS = Medicare Benefits Schedule.

Supplementary Table 4. Baseline Characteristics of Study Cohort by GP After-Hours Attendances Before and After Weighting

	Overall Cohort			*Weighted Cohort*[1]		
**Exposure Status**	**Overall Cohort**	**No GP after-hours attendance**	**GP after-hours attendance**	**No GP after-hours attendance**	**GP after-hours attendance**	**Standardised Mean Differences**[2]
**Total, N / Sum of Weights**	120522	96818	23704	81279	81279	
**Individual characteristics**						
**Sex male, N (%)**	47007 (39)	37699 (38.9)	9308 (39.3)	31432 (38.7)	31290 (38.5)	-0.001
**Age at entry, Mean (SD), years**	81.8 (7.3)	81.8 (7.3)	81.8 (7.4)	81.5 (7.2)	81.6 (7.4)	0.009
**Culturally and linguistical diverse, N (%)**	15953 (13.2)	11010 (11.4)	4943 (20.9)	11092 (13.6)	10977 (13.5)	<0.001
**Partner status, N (%)**						
**Divorced**	10750 (8.9)	8697 (9)	2053 (8.7)	7367 (9.1)	7543 (9.3)	<0.001
**Married**	58049 (48.2)	46632 (48.2)	11417 (48.2)	39247 (48.3)	39272 (48.3)	-0.001
**Never married**	6994 (5.8)	5720 (5.9)	1274 (5.4)	4623 (5.7)	4495 (5.5)	-0.001
**Separated**	880 (0.7)	746 (0.8)	134 (0.6)	592 (0.7)	376 (0.5)	R[4]
**Unable to determine**	1466 (1.2)	1201 (1.2)	265 (1.1)	945 (1.2)	861 (1.1)	R[4]
**Widowed**	42383 (35.2)	33822 (34.9)	8561 (36.1)	28505 (35.1)	28733 (35.4)	R[4]
**Health conditions, N (%)**						
**Dementia**	23686 (19.7)	18527 (19.1)	5159 (21.8)	15052 (18.5)	15278 (18.8)	0.003
**History of pressure injuries**	5921 (4.9)	4669 (4.8)	1252 (5.3)	3506 (4.3)	3444 (4.2)	-0.001
**Incontinence**	16119 (13.4)	12516 (12.9)	3603 (15.2)	9993 (12.3)	9851 (12.1)	-0.001
**History of cancer**	22187 (18.4)	17850 (18.4)	4337 (18.3)	13831 (17)	13513 (16.6)	-0.003
**History of pain**	54780 (45.5)	43472 (44.9)	11308 (47.7)	36573 (45)	36611 (45)	0.001
**Chronic respiratory disease**	35738 (29.7)	28274 (29.2)	7464 (31.5)	23889 (29.4)	23917 (29.4)	-0.001
**Heart failure**	25598 (21.2)	20215 (20.9)	5383 (22.7)	16971 (20.9)	17210 (21.2)	0.002
**Depression**	49088 (40.7)	39030 (40.3)	10058 (42.4)	32883 (40.5)	33147 (40.8)	0.002
**Diabetes**	32340 (26.8)	25354 (26.2)	6986 (29.5)	21668 (26.7)	21368 (26.3)	-0.003
**Ischemic heart disease / hypertension**	85856 (71.2)	68662 (70.9)	17194 (72.5)	57164 (70.3)	56959 (70.1)	-0.003
**Osteoporosis**	34079 (28.3)	27105 (28)	6974 (29.4)	22558 (27.8)	22594 (27.8)	0.001
**Mean number of additional health conditions (SD)**[3]	2.5 (1.6)	2.5 (1.6)	2.6 (1.6)	2.5 (1.6)	2.5 (1.6)	-0.007
**SEIFA index of relative socioeconomic disadvantage quintile, N (%)**						0.008
**1 – disadvantaged**	24300 (20.2)	19611 (20.3)	4689 (19.8)	16303 (20.1)	16585 (20.4)	
**2**	25079 (20.9)	20828 (21.6)	4251 (18)	16946 (20.8)	16687 (20.5)	
**3**	24877 (20.7)	20249 (21)	4628 (19.6)	16873 (20.8)	16246 (20)	
**4**	21706 (18.1)	16991 (17.6)	4715 (19.9)	14834 (18.3)	14786 (18.2)	
**5 – advantaged**	24196 (20.1)	18811 (19.5)	5385 (22.8)	16323 (20.1)	16976 (20.9)	
**SEIFA index of education and occupation quintiles, N (%)**						0.007
**1 – Low**	25578 (21.3)	20952 (21.7)	4626 (19.5)	17012 (20.9)	16785 (20.7)	
**2**	23026 (19.2)	19013 (19.7)	4013 (17)	15523 (19.1)	15754 (19.4)	
**3**	22916 (19.1)	18694 (19.4)	4222 (17.8)	15702 (19.3)	15294 (18.8)	
**4**	20308 (16.9)	15948 (16.5)	4360 (18.4)	13869 (17.1)	13933 (17.1)	
**5 – High**	28330 (23.6)	21883 (22.7)	6447 (27.2)	19173 (23.6)	19512 (24)	

**Care level at entry, N (%)**						0.011
**Level1**	18455 (15.3)	15618 (16.1)	2837 (12)	14696 (18.1)	13434 (16.5)	
**Level 2**	74253 (61.6)	59700 (61.7)	14553 (61.4)	49083 (60.4)	50826 (62.5)	
**Level 3**	16644 (13.8)	12962 (13.4)	3682 (15.5)	11692 (14.4)	11558 (14.2)	
**Level 4**	11170 (9.3)	8538 (8.8)	2632 (11.1)	5808 (7.1)	5462 (6.7)	
**Maximally approved care level, N (%)**						0.002
**No approved level**	11992 (10)	9458 (9.8)	2534 (10.7)	5942 (7.3)	6403 (7.9)	
**Level 1**	2460 (2)	2154 (2.2)	306 (1.3)	1964 (2.4)	1797 (2.2)	
**Level 2**	46302 (38.5)	38254 (39.5)	8048 (34)	31585 (38.9)	31229 (38.4)	
**Level 3**	21391 (17.8)	17470 (18.1)	3921 (16.6)	16770 (20.6)	15757 (19.4)	
**Level 4**	38273 (31.8)	29399 (30.4)	8874 (37.5)	25018 (30.8)	26093 (32.1)	
**Approval for long-term care facility, N (%)**	86023 (71.4)	68392 (70.6)	17631 (74.4)	58296 (71.7)	58478 (71.9)	0.003
**Approval for respite care, N (%)**	108156 (89.7)	86509 (89.4)	21647 (91.3)	73090 (89.9)	73329 (90.2)	0.003
**Wait time, logarithmized median [IQR]**	5.9 [5.2, 6.4]	5.9 [5.2, 6.4]	5.9 [5, 6.4]	5.7 [4.9, 6.2]	5.7 [4.8, 6.2]	0.002
**Facility Characteristics**						
**State of service, N (%)**						
**Australian Capital Territory**	1691 (1.4)	1457 (1.5)	234 (1)	1034 (1.3)	1082 (1.3)	<0.001
**New South Wales**	41972 (34.8)	34211 (35.3)	7761 (32.7)	27773 (34.2)	27367 (33.7)	R
**Northern Territory**	278 (0.2)	227 (0.2)	51 (0.2)	157 (0.2)	152 (0.2)	<0.001
**Queensland**	25578 (21.2)	20593 (21.3)	4985 (21)	16667 (20.5)	16895 (20.8)	0.001
**South Australia**	9366 (7.8)	7167 (7.4)	2199 (9.3)	6345 (7.8)	6312 (7.8)	0.001
**Tasmania**	2791 (2.3)	2485 (2.6)	306 (1.3)	1695 (2.1)	1847 (2.3)	0.002
**Victoria**	27637 (22.9)	21221 (21.9)	6416 (27.1)	20570 (25.3)	20536 (25.3)	0.001
**Western Australia**	11209 (9.3)	9457 (9.8)	1752 (7.4)	7038 (8.7)	7087 (8.7)	0.001
**Remoteness, N (%)**						
**Major Cities**	82701 (68.6)	63842 (65.9)	18859 (79.6)	56759 (69.8)	56134 (69.1)	R
**Inner Regional**	29365 (24.4)	25657 (26.5)	3708 (15.6)	19236 (23.7)	19650 (24.2)	0.005
**Outer Regional**	7756 (6.4)	6715 (6.9)	1041 (4.4)	4877 (6)	5077 (6.2)	0.001
**Remote**	531 (0.4)	468 (0.5)	63 (0.3)	343 (0.4)	302 (0.4)	0.000
**Very Remote**	133 (0.1)	109 (0.1)	24 (0.1)	65 (0.1)	116 (0.1)	- [5]
**Missing**	36 (0)	27 (0)	9 (0)	0 (0)	0 (0)	
**Ownership, N (%)**						
**Government**	7881 (6.5)	6545 (6.8)	1336 (5.6)	5422 (6.7)	5417 (6.7)	0.001
**Not-for-profit**	85721 (71.1)	68740 (71)	16981 (71.6)	57604 (70.9)	57879 (71.2)	R
**Private**	26920 (22.3)	21533 (22.2)	5387 (22.7)	18253 (22.5)	17984 (22.1)	-0.001
**History of GP after-hours attendance one year prior to entry, N (%)**		13980 (14.4)	10707 (45.2)	17557 (21.6)	17760 (21.9)	0.001

R=Reference. SD=Standard deviation. SEIFA=Socio-Economic Indexes for Areas.

1. Fine stratification weighted with 50 strata and weights targeting the average treatment effect.
2. Standardised mean differences <0.10 are considered good balance.
3. Excludes health conditions listed in the table.
4. Combined reference category.
5. Very remote and remote categories were collapsed for the purpose of weighting due to small cell numbers in very remote categories.

Supplementary Table 5. Baseline Characteristics of Study Cohort by Urgent After-Hours GP Attendances Before and After Weighting

		Overall Cohort		*Weighted Cohort*[1]		
**Exposure Status**	**Overall Cohort**	**No Urgent after-hours attendance**	**Urgent after-hours attendance**	**No Urgent after-hours attendance**	**Urgent after-hours attendance**	**Standardised Mean Differences**[2]
**Total, N / Sum of Weights**	120522	109918	10604	7892	7892	
**Individual characteristics**						
**Sex male, N (%)**	47007 (39)	42925 (39.1)	4082 (38.5)	3073 (38.9)	3077 (39)	-0.001
**Age at entry, Mean (SD), years**	81.8 (7.3)	81.8 (7.3)	82.2 (7.6)	82.2 (7.3)	82.1 (7.6)	-0.007
**Culturally and linguistical diverse, N (%)**	15953 (13.2)	14074 (12.8)	1879 (17.7)	1339 (17)	1372 (17.4)	0.003
**Partner status, N (%)**						
**Divorced**	10750 (8.9)	9761 (8.9)	989 (9.3)	725 (9.2)	740 (9.4)	<0.001
**Married**	58049 (48.2)	53224 (48.4)	4825 (45.5)	3606 (45.7)	3598 (45.6)	-0.001
**Never married**	6994 (5.8)	6369 (5.8)	625 (5.9)	447 (5.7)	452 (5.7)	<0.001
**Separated**	880 (0.7)	811 (0.7)	69 (0.7)	60 (0.8)	46 (0.6)	R[4]
**Unable to determine**	1466 (1.2)	1354 (1.2)	112 (1.1)	92 (1.2)	78 (1)	R[4]
**Widowed**	42383 (35.2)	38399 (34.9)	3984 (37.6)	2962 (37.5)	2978 (37.7)	R[4]
**Select health conditions, N (%)**						
**Dementia**	23686 (19.7)	21265 (19.3)	2421 (22.8)	1814 (23)	1791 (22.7)	-0.003
**History of pressure injuries**	5921 (4.9)	5250 (4.8)	671 (6.3)	450 (5.7)	455 (5.8)	<0.001
**Incontinence**	16119 (13.4)	14364 (13.1)	1755 (16.6)	1147 (14.5)	1160 (14.7)	0.001
**History of cancer**	22187 (18.4)	20178 (18.4)	2009 (18.9)	15702 (17.6)	1997 (19)	-0.001
**History of pain**	54780 (45.5)	49562 (45.1)	5218 (49.2)	3785 (48)	3798 (48.1)	0.001
**Chronic respiratory disease**	35738 (29.7)	32174 (29.3)	3564 (33.6)	26079 (29.2)	3544 (33.6)	0.001
**Heart failure**	25598 (21.2)	22912 (20.8)	2686 (25.3)	18187 (20.4)	2672 (25.4)	-0.001
**Depression**	49088 (40.7)	44360 (40.4)	4728 (44.6)	3452 (43.7)	3455 (43.8)	0.001
**Diabetes,**	32340 (26.8)	29246 (26.6)	3094 (29.2)	2221 (28.1)	2232 (28.3)	<0.001
**Ischemic heart disease / hypertension**	85856 (71.2)	78237 (71.2)	7619 (71.9)	5593 (70.9)	5602 (71)	0.001
**Osteoporosis**	34079 (28.3)	31019 (28.2)	3060 (28.9)	2247 (28.5)	2236 (28.3)	<0.001
**Mean number of additional health conditions (SD)**[3]	2.5 (1.6)	2.5 (1.6)	2.7 (1.7)	2.7 (1.6)	2.7 (1.7)	-0.001
**SEIFA index of relative socioeconomic disadvantage quintile, N (%)**						-0.009
**1 – disadvantaged**	24300 (20.2)	22314 (20.4)	1986 (18.8)	1423 (18)	1512 (19.2)	
**2**	25079 (20.9)	23240 (21.2)	1839 (17.4)	1414 (17.9)	1364 (17.3)	
**3**	24877 (20.7)	22844 (20.8)	2033 (19.2)	1583 (20.1)	1514 (19.2)	
**4**	21706 (18.1)	19405 (17.7)	2301 (21.7)	1654 (21)	1693 (21.5)	
**5 – advantaged**	24196 (20.1)	21767 (19.9)	2429 (22.9)	1818 (23)	1809 (22.9)	
**SEIFA index of education and occupation quintiles, N (%)**						-0.010
**1 – Low**	25578 (21.3)	23693 (21.6)	1885 (17.8)	1409 (17.9)	1434 (18.2)	
**2**	23026 (19.2)	21420 (19.5)	1606 (15.2)	1263 (16)	1208 (15.3)	
**3**	22916 (19.1)	20887 (19.1)	2029 (19.2)	1472 (18.7)	1499 (19)	
**4**	20308 (16.9)	18134 (16.6)	2174 (20.5)	1511 (19.2)	1644 (20.8)	
**5 – High**	28330 (23.6)	25436 (23.2)	2894 (27.3)	2236 (28.3)	2107 (26.7)	

**Care level at entry, N (%)**						-0.007
**Level 1**	18455 (15.3)	17637 (16)	818 (7.7)	855 (10.8)	756 (9.6)	
**Level 2**	74253 (61.6)	67835 (61.7)	6418 (60.5)	4515 (57.2)	4747 (60.1)	
**Level 3**	16644 (13.8)	14866 (13.5)	1778 (16.8)	1531 (19.4)	1407 (17.8)	
**Level 4**	11170 (9.3)	9580 (8.7)	1590 (15)	992 (12.6)	982 (12.4)	
**Maximally approved care level, N (%)**						-0.004
**No approved level**	11992 (10)	10683 (9.7)	1309 (12.4)	622 (7.9)	720 (9.1)	
**Level 1**	2460 (2)	2401 (2.2)	59 (0.6)	76 (1)	54 (0.7)	
**Level 2**	46302 (38.5)	43309 (39.4)	2993 (28.3)	2167 (27.5)	2147 (27.2)	
**Level 3**	21391 (17.8)	19967 (18.2)	1424 (13.4)	1542 (19.5)	1320 (16.7)	
**Level 4**	38273 (31.8)	33464 (30.5)	4809 (45.4)	3485 (44.2)	3651 (46.3)	
**Approval for long-term care facility, N (%)**	86023 (71.4)	77753 (70.7)	8270 (78)	6310 (80)	6280 (79.6)	-0.004
**Approval for respite care, N (%)**	108156 (89.7)	98328 (89.5)	9828 (92.7)	7382 (93.5)	7366 (93.3)	-0.001
**Wait time, logarithmized median [IQR]**	5.9 [5.2, 6.4]	5.9 [5.2, 6.4]	5.9 [4.7, 6.5]	5.6 [4.4, 6.2]	5.6 [4.3, 6.2]	0.004
**Facility Characteristics**						
**State of service, N (%)**						
**Australian Capital Territory**	1691 (1.4)	1613 (1.5)	78 (0.7)	51 (0.6)	52 (0.7)	<0.001
**New South Wales**	41972 (34.8)	39045 (35.5)	2927 (27.6)	2109 (26.7)	2141 (27.1)	R
**Northern Territory**	278 (0.2)	268 (0.2)	10 (0.1)	7 (0.1)	7 (0.1)	<0.001
**Queensland**	25578 (21.2)	23062 (21)	2516 (23.7)	1838 (23.3)	1811 (22.9)	-0.004
**South Australia**	9366 (7.8)	8228 (7.5)	1138 (10.7)	875 (11.1)	868 (11)	-0.001
**Tasmania**	2791 (2.3)	2638 (2.4)	153 (1.4)	105 (1.3)	109 (1.4)	<0.001
**Victoria**	27637 (22.9)	24852 (22.6)	2785 (26.3)	2186 (27.7)	2186 (27.7)	0.001
**Western Australia**	11209 (9.3)	10212 (9.3)	997 (9.4)	723 (9.2)	718 (9.1)	<0.001
**Remoteness, N (%)**						
**Major Cities**	82701 (68.6)	74162 (67.5)	8539 (80.5)	6393 (81)	6359 (80.6)	R
**Inner Regional**	29365 (24.4)	27727 (25.2)	1638 (15.4)	1190 (15.1)	1216 (15.4)	0.003
**Outer Regional**	7756 (6.4)	7363 (6.7)	393 (3.7)	282 (3.6)	291 (3.7)	0.001
**Remote**	531 (0.4)	*	*	*	*	<0.001
**Very Remote**	133 (0.1)	*	*	*	*	- [5]
**Missing**	36 (0)	*	*	0 (0)	0 (0)	
**Ownership, N (%)**						
**Government**	7881 (6.5)	7256 (6.6)	625 (5.9)	454 (5.8)	453 (5.7)	<0.001
**Not-for-profit**	85721 (71.1)	77936 (70.9)	7785 (73.4)	5778 (73.2)	5765 (73)	R
**Private**	26920 (22.3)	24726 (22.5)	2194 (20.7)	1660 (21)	1674 (21.2)	0.002
**History of Urgent After-Hours GP attendance one year prior to entry, N (%)**		8966 (8.16)	3351 (31.6)	2398 (30.4)	2468 (31.3)	0.007

R=Reference. SD=Standard deviation. SEIFA=Socio-Economic Indexes for Areas.

1. Fine stratification weighted with 50 strata and weights targeting the average treatment effect.
2. Standardised mean differences <0.10 are considered good balance.
3. Excludes health conditions included in the table.
4. Combined reference category.
5. Very remote and remote categories were collapsed for the purpose of weighting due to small cell numbers in very remote categories.

Supplementary Table 6. Baseline Characteristics of Study Cohort by Health Assessment Utilization Before and After Weighting

		Overall Cohort		*Weighted Cohort[1]*		
**Exposure Status**	**Overall Cohort**	**No Health Assessment**	**Health Assessment**	**No Health Assessment**	**Health Assessment**	**Standardised Mean Differences[2]**
**Total, N / Sum of Weights**	120522	93237	27285	60911	60911	
**Individual characteristics**						
**Sex male, N (%)**	47007 (39)	36742 (39.4)	10265 (37.6)	23443 (38.5)	23589 (38.7)	0.001
**Age at entry, Mean (SD), years**	81.8 (7.3)	81.2 (7.7)	83.7 (5.5)	84 (5.3)	84 (5.2)	-0.003
**Culturally and linguistical diverse, N (%)**	15953 (13.2)	12881 (13.8)	3072 (11.3)	8774 (14.4)	8784 (14.4)	-0.001
**Partner status, N (%)**						
**Divorced**	10750 (8.9)	8857 (9.5)	1893 (6.9)	4176 (6.9)	4251 (7)	0.001
**Married**	58049 (48.2)	44789 (48)	13260 (48.6)	29410 (48.3)	29475 (48.4)	<0.001
**Never married**	6994 (5.8)	5754 (6.2)	1240 (4.5)	2537 (4.2)	2529 (4.2)	<0.001
**Separated**	880 (0.7)	732 (0.8)	148 (0.5)	323 (0.5)	308 (0.5)	R[4]
**Unable to determine**	1466 (1.2)	1195 (1.3)	271 (1)	652 (1.1)	581 (1)	R[4]
**Widowed**	42383 (35.2)	31910 (34.2)	10473 (38.4)	23813 (39.1)	23768 (39)	R[4]
**Select health conditions N (%)**						
**Dementia,**	23686 (19.7)	18705 (20.1)	4981 (18.3)	11495 (18.9)	11663 (19.1)	0.003
**History of pressure injuries**	5921 (4.9)	4684 (5)	1237 (4.5)	2569 (4.2)	2585 (4.2)	<0.001
**Incontinence**	16119 (13.4)	12398 (13.3)	3721 (13.6)	7376 (12.1)	7352 (12.1)	<0.001
**History of cancer**	22187 (18.4)	17140 (18.4)	5047 (18.5)	10639 (17.5)	10663 (17.5)	<0.001
**History of pain**	54780 (45.5)	42466 (45.5)	12314 (45.1)	26487 (43.5)	26537 (43.6)	<0.001
**Chronic respiratory disease**	35738 (29.7)	27597 (29.6)	8141 (29.8)	17138 (28.1)	17056 (28)	-0.001
**Heart failure**	25598 (21.2)	19697 (21.1)	5901 (21.6)	13179 (21.6)	13149 (21.6)	<0.001
**Depression**	49088 (40.7)	38367 (41.1)	10721 (39.3)	22582 (37.1)	22637 (37.2)	0.001
**Diabetes**	32340 (26.8)	25332 (27.2)	7008 (25.7)	15342 (25.2)	15383 (25.3)	<0.001
**Ischemic heart disease / hypertension**	85856 (71.2)	65792 (70.6)	20064 (73.5)	43889 (72.1)	43960 (72.2)	0.002
**Osteoporosis**	34079 (28.3)	25270 (27.1)	8809 (32.3)	18190 (29.9)	18120 (29.7)	<0.001
**Mean number of additional health conditions (SD)[3]**	2.5 (1.6)	2.5 (1.6)	2.5 (1.5)	2.5 (1.5)	2.5 (1.5)	-0.003
**SEIFA index of relative socioeconomic disadvantage quintile, N (%)**						-0.001
**1 – disadvantaged**	24300 (20.2)	19056 (20.5)	5244 (19.2)	12324 (20.2)	11467 (18.8)	
**2**	25079 (20.9)	18962 (20.4)	6117 (22.4)	12454 (20.4)	13231 (21.7)	
**3**	24877 (20.7)	18878 (20.3)	5999 (22)	12360 (20.3)	12994 (21.3)	
**4**	21706 (18.1)	16873 (18.2)	4833 (17.7)	11169 (18.3)	10998 (18.1)	
**5 – advantaged**	24196 (20.1)	19134 (20.6)	5062 (18.6)	12603 (20.7)	12221 (20.1)	
**SEIFA index of education and occupation quintiles, N (%)**						-0.001
**1 – Low**	25578 (21.3)	19929 (21.5)	5649 (20.7)	12719 (20.9)	11966 (19.6)	
**2**	23026 (19.2)	17532 (18.9)	5494 (20.2)	11432 (18.8)	12093 (19.9)	
**3**	22916 (19.1)	17366 (18.7)	5550 (20.4)	11525 (18.9)	12023 (19.7)	
**4**	20308 (16.9)	15662 (16.9)	4646 (17)	10416 (17.1)	10466 (17.2)	
**5 – High**	28330 (23.6)	22414 (24.1)	5916 (21.7)	14819 (24.3)	14363 (23.6)	

**Care level at entry, N (%)**						<0.001
**Level 1**	18455 (15.3)	14340 (15.4)	4115 (15.1)	11387 (18.7)	10300 (16.9)	
**Level 2**	74253 (61.6)	56220 (60.3)	18033 (66.1)	37009 (60.8)	38751 (63.6)	
**Level 3**	16644 (13.8)	13485 (14.5)	3159 (11.6)	8441 (13.9)	8180 (13.4)	
**Level 4**	11170 (9.3)	9192 (9.9)	1978 (7.2)	4075 (6.7)	3679 (6)	
**Maximally approved care level, N (%)**						0.001
**No approved level**	11992 (10)	9309 (10)	2683 (9.8)	4531 (7.4)	4540 (7.5)	
**Level 1**	2460 (2)	1938 (2.1)	522 (1.9)	1516 (2.5)	1266 (2.1)	
**Level 2**	46302 (38.5)	34248 (36.8)	12054 (44.2)	24393 (40)	24844 (40.8)	
**Level 3**	21391 (17.8)	16870 (18.1)	4521 (16.6)	12399 (20.4)	12199 (20)	
**Level 4**	38273 (31.8)	30793 (33.1)	7480 (27.4)	18072 (29.7)	18063 (29.7)	
**Approval for long-term care facility, N (%)**	86023 (71.4)	66493 (71.3)	19530 (71.6)	44856 (73.6)	44854 (73.6)	<0.001
**Approval for respite care, N (%)**	108156 (89.7)	83391 (89.4)	24765 (90.8)	55655 (91.4)	55598 (91.3)	-0.001
**Wait time, logarithmized median [IQR]**	5.9 [5.2, 6.4]	5.9 [5.2, 6.4]	5.9 [5.2, 6.4]	5.7 [5, 6.2]	5.8 [4.9, 6.2]	0.003
**Facility Characteristics**						
**State of service, N (%)**						
**Australian Capital Territory**	1691 (1.4)	1408 (1.5)	283 (1)	763 (1.3)	806 (1.3)	<0.001
**New South Wales**	41972 (34.8)	31953 (34.3)	10019 (36.7)	21250 (34.9)	21257 (34.9)	R
**Northern Territory**	278 (0.2)	263 (0.3)	15 (0.1)	101 (0.2)	87 (0.1)	<0.001
**Queensland**	25578 (21.2)	19026 (20.4)	6552 (24)	12287 (20.2)	12261 (20.1)	<0.001
**South Australia**	9366 (7.8)	7214 (7.7)	2152 (7.9)	4817 (7.9)	4831 (7.9)	<0.001
**Tasmania**	2791 (2.3)	2164 (2.3)	627 (2.3)	1297 (2.1)	1308 (2.1)	<0.001
**Victoria**	27637 (22.9)	22291 (23.9)	5346 (19.6)	15005 (24.6)	14988 (24.6)	0.001
**Western Australia**	11209 (9.3)	8918 (9.6)	2291 (8.4)	5391 (8.9)	5372 (8.8)	0.001
**Remoteness, N (%)**						
**Major Cities**	82701 (68.6)	64210 (68.9)	18491 (67.8)	42220 (69.3)	42193 (69.3)	R
**Inner Regional**	29365 (24.4)	22404 (24)	6961 (25.5)	14811 (24.3)	14825 (24.3)	0.001
**Outer Regional**	7756 (6.4)	6041 (6.5)	1715 (6.3)	3580 (5.9)	3574 (5.9)	<0.001
**Remote**	531 (0.4)	443 (0.5)	88 (0.3)	248 (0.4)	265 (0.4)	<0.001
**Very Remote**	133 (0.1)	111 (0.1)	22 (0.1)	52 (0.1)	55 (0.1)	- [5]
**Missing**	36 (0)	28 (0)	8 (0)	0 (0)	0 (0)	
**Ownership, N (%)**						
**Government**	7881 (6.5)	6409 (6.9)	1472 (5.4)	3942 (6.5)	3945 (6.5)	0.001
**Not-for-profit**	85721 (71.1)	65963 (70.7)	19758 (72.4)	43254 (71)	43242 (71)	R
**Private**	26920 (22.3)	20865 (22.4)	6055 (22.2)	13715 (22.5)	13724 (22.5)	<0.001
**History of Health Assessment one year prior to entry, N (%)**		27292 (29.3)	14963 (54.8)	22929 (37.6)	23054 (37.8)	0.001

R=Reference. SD=Standard deviation. SEIFA=Socio-Economic Indexes for Areas.

1. Fine stratification weighted with 50 strata and weights targeting the average treatment effect.
2. Standardised mean differences <0.10 are considered good balance.
3. Excludes health conditions included in the table.
4. Combined reference category.
5. Very remote and remote categories were collapsed for the purpose of weighting due to small cell numbers in very remote categories.

Supplementary Table 7. Baseline Characteristics of Study Cohort by Management Plan Utilization Before and After Weighting

		Overall Cohort		*Weighted Cohort[1]*		
**Exposure Status**	**Overall Cohort**	**No Management plans**	**Management plans**	**No Management plans**	**Management plans**	**Standardised Mean Differences[2]**
**Total, N / Sum of Weights**	120522	61108	59414	82886	82886	
**Recipient characteristics**						
**Sex male, N (%)**	47007 (39)	24420 (40)	22587 (38)	32266 (38.9)	32216 (38.9)	<0.001
**Age at entry, Mean (SD), years**	81.8 (7.3)	82.2 (7.4)	81.4 (7.1)	81.5 (7.3)	81.5 (7.2)	0.001
**Culturally and linguistical diverse, N (%)**	15953 (13.2)	8421 (13.8)	7532 (12.7)	11451 (13.8)	11465 (13.8)	<0.001
**Partner status, N (%)**						
**Divorced**	10750 (8.9)	5430 (8.9)	5320 (9)	7576 (9.1)	7563 (9.1)	<0.001
**Married**	58049 (48.2)	29255 (47.9)	28794 (48.5)	40015 (48.3)	40035 (48.3)	<0.001
**Never married**	6994 (5.8)	3615 (5.9)	3379 (5.7)	4753 (5.7)	4765 (5.7)	<0.001
**Separated**	880 (0.7)	436 (0.7)	444 (0.7)	583 (0.7)	582 (0.7)	R[4]
**Unable to determine**	1466 (1.2)	811 (1.3)	655 (1.1)	986 (1.2)	956 (1.2)	R[4]
**Widowed**	42383 (35.2)	21561 (35.3)	20822 (35)	28972 (35)	28986 (35)	R[4]
**Select health conditions, N (%)**						
**Dementia**	23686 (19.7)	13501 (22.1)	10185 (17.1)	15380 (18.6)	15329 (18.5)	<0.001
**History of pressure injuries**	5921 (4.9)	3057 (5)	2864 (4.8)	3687 (4.4)	3660 (4.4)	<0.001
**Incontinence**	16119 (13.4)	7840 (12.8)	8279 (13.9)	10254 (12.4)	10249 (12.4)	<0.001
**History of cancer**	22187 (18.4)	11396 (18.6)	10791 (18.2)	14268 (17.2)	14291 (17.2)	<0.001
**History of pain**	54780 (45.5)	26435 (43.3)	28345 (47.7)	37440 (45.2)	37420 (45.1)	-0.001
**Chronic respiratory disease**	35738 (29.7)	17202 (28.2)	18536 (31.2)	24428 (29.5)	24378 (29.4)	<0.001
**Heart failure**	25598 (21.2)	12533 (20.5)	13065 (22)	17483 (21.1)	17489 (21.1)	<0.001
**Depression**	49088 (40.7)	24095 (39.4)	24993 (42.1)	33573 (40.5)	33553 (40.5)	<0.001
**Diabetes**	32340 (26.8)	14391 (23.6)	17949 (30.2)	22417 (27)	22314 (26.9)	<0.001
**Ischemic heart disease / hypertension**	85856 (71.2)	42635 (69.8)	43221 (72.7)	58501 (70.6)	58525 (70.6)	<0.001
**Osteoporosis**	34079 (28.3)	16529 (27)	17550 (29.5)	23067 (27.8)	23088 (27.9)	<0.001
**Mean number of additional health conditions (SD)[3]**	2.5 (1.6)	2.4 (1.6)	2.6 (1.6)	2.5 (1.6)	2.5 (1.6)	-0.001
**SEIFA index of relative socioeconomic disadvantage quintile, N (%)**						<0.001
**1 – disadvantaged**	24300 (20.2)	12023 (19.8)	12277 (20.7)	17417 (21)	16446 (19.8)	
**2**	25079 (20.9)	12052 (19.8)	13027 (22)	16917 (20.4)	17507 (21.1)	
**3**	24877 (20.7)	12012 (19.8)	12865 (21.7)	16669 (20.1)	17486 (21.1)	
**4**	21706 (18.1)	11082 (18.2)	10624 (17.9)	14689 (17.7)	15173 (18.3)	
**5 – advantaged**	24196 (20.1)	13646 (22.4)	10550 (17.8)	17194 (20.7)	16274 (19.6)	
**SEIFA index of education and occupation quintiles, N (%)**						0.001
**1 – Low**	25578 (21.3)	12555 (20.6)	13023 (21.9)	18033 (21.8)	17129 (20.7)	
**2**	23026 (19.2)	11161 (18.4)	11865 (20)	15800 (19.1)	15883 (19.2)	
**3**	22916 (19.1)	10768 (17.7)	12148 (20.5)	15054 (18.2)	16624 (20.1)	
**4**	20308 (16.9)	10420 (17.1)	9888 (16.7)	13951 (16.8)	14173 (17.1)	
**5 – High**	28330 (23.6)	15911 (26.2)	12419 (20.9)	20047 (24.2)	19077 (23)	

**Care level at entry, N (%)**						0.002
**Level 1**	18455 (15.3)	9288 (15.2)	9167 (15.4)	16231 (19.6)	14298 (17.2)	
**Level 2**	74253 (61.6)	36133 (59.1)	38120 (64.2)	48075 (58)	51687 (62.4)	
**Level 3**	16644 (13.8)	9373 (15.3)	7271 (12.2)	12550 (15.1)	11151 (13.5)	
**Level 4**	11170 (9.3)	6314 (10.3)	4856 (8.2)	6031 (7.3)	5751 (6.9)	
**Maximally approved care level, N (%)**						<0.001
**No approved level**	11992 (10)	6182 (10.1)	5810 (9.8)	6215 (7.5)	6264 (7.6)	
**Level 1**	2460 (2)	1284 (2.1)	1176 (2)	2365 (2.9)	1765 (2.1)	
**Level 2**	46302 (38.5)	21558 (35.3)	24744 (41.7)	31323 (37.8)	32654 (39.4)	
**Level 3**	21391 (17.8)	11356 (18.6)	10035 (16.9)	17601 (21.2)	16743 (20.2)	
**Level 4**	38273 (31.8)	20689 (33.9)	17584 (29.6)	25383 (30.6)	25460 (30.7)	
**Approval for long-term care facility, N (%)**	86023 (71.4)	45058 (73.7)	40965 (68.9)	59302 (71.5)	59334 (71.6)	<0.001
**Approval for respite care, N (%)**	108156 (89.7)	55254 (90.4)	52902 (89)	74463 (89.8)	74475 (89.9)	<0.001
**Wait time, logarithmized median [IQR]**	5.9 [5.2, 6.4]	5.9 [5.2, 6.4]	5.9 [5.2, 6.4]	5.7 [4.9, 6.2]	5.7 [4.9, 6.2]	-0.001
**Facility Characteristics**						
**State of service, N (%)**						
**Australian Capital Territory**	1691 (1.4)	1166 (1.9)	525 (0.9)	1052 (1.3)	966 (1.2)	-0.001
**New South Wales**	41972 (34.8)	20618 (33.7)	21354 (35.9)	28660 (34.6)	28693 (34.6)	R
**Northern Territory**	278 (0.2)	202 (0.3)	76 (0.1)	173 (0.2)	166 (0.2)	<0.001
**Queensland**	25578 (21.2)	12901 (21.1)	12677 (21.3)	16745 (20.2)	16777 (20.2)	<0.001
**South Australia**	9366 (7.8)	4725 (7.7)	4641 (7.8)	6333 (7.6)	6322 (7.6)	<0.001
**Tasmania**	2791 (2.3)	1431 (2.3)	1360 (2.3)	1807 (2.2)	1808 (2.2)	<0.001
**Victoria**	27637 (22.9)	14187 (23.2)	13450 (22.6)	20856 (25.2)	20932 (25.3)	<0.001
**Western Australia**	11209 (9.3)	5878 (9.6)	5331 (9)	7260 (8.8)	7223 (8.7)	<0.001
**Remoteness, N (%)**						
**Major Cities**	82701 (68.6)	42658 (69.8)	40043 (67.4)	57653 (69.6)	57617 (69.5)	R
**Inner Regional**	29365 (24.4)	13903 (22.8)	15462 (26)	19820 (23.9)	19882 (24)	0.001
**Outer Regional**	7756 (6.4)	4082 (6.7)	3674 (6.2)	5000 (6)	4996 (6)	<0.001
**Remote**	531 (0.4)	357 (0.6)	174 (0.3)	336 (0.4)	322 (0.4)	<0.001
**Very Remote**	133 (0.1)	85 (0.1)	48 (0.1)	77 (0.1)	69 (0.1)	-[5]
**Missing**	36 (0)	23 (0)	13 (0)	0 (0)	0 (0)	
**Ownership, N (%)**						
**Government**	7881 (6.5)	4187 (6.9)	3694 (6.2)	5467 (6.6)	5468 (6.6)	<0.001
**Not-for-profit**	85721 (71.1)	43480 (71.2)	42241 (71.1)	58665 (70.8)	58620 (70.7)	R
**Private**	26920 (22.3)	13441 (22)	13479 (22.7)	18753 (22.6)	18798 (22.7)	<0.001
**History of Management Plans in one year prior to entry, N (%)**		28363 (46.4)	46786 (78.7)	53691 (64.8)	53686 (64.8)	<0.001

R=Reference. SD=Standard deviation. SEIFA=Socio-Economic Indexes for Areas.

1. Fine stratification weighted with 50 strata and weights targeting the average treatment effect.
2. Standardised mean differences <0.10 are considered good balance.
3. Excludes health conditions included in the table.
4. Combined reference category.
5. Very remote and remote categories were collapsed for the purpose of weighting due to small cell numbers in very remote categories.

Supplementary Table 8. Baseline Characteristics of Study Cohort by Podiatry Utilization Before and After Weighting

		Overall Cohort		*Weighted Cohort*[1]		
**Exposure Status**	**Overall Cohort**	**No Podiatry**	**Podiatry**	**No Podiatry**	**Podiatry**	**Standardised Mean Differences**[2]
**Total, N / Sum of Weights**	120522	74789	45733	35498	35498	
**Individual characteristics**						
**Sex male, N (%)**	130547 (39.4)	91805 (40.3)	38742 (37.4)	13780 (38.8)	13744 (38.7)	-0.002
**Age at entry, Mean (SD), years**	83.8 (7.2)	83.8 (7.3)	83.8 (7.1)	82.1 (7.1)	82.2 (6.9)	0.023
**Culturally and linguistical diverse, N (%)**	15953 (13.2)	10204 (13.6)	5749 (12.6)	4486 (12.6)	4455 (12.6)	-0.001
**Partner status, N (%)**						
**Divorced**	10750 (8.9)	7210 (9.6)	3540 (7.7)	2883 (8.1)	2802 (7.9)	-0.001
**Married**	58049 (48.2)	35440 (47.4)	22609 (49.4)	17674 (49.8)	17675 (49.8)	-0.003
**Never married**	6994 (5.8)	4691 (6.3)	2303 (5)	1756 (4.9)	1716 (4.8)	-0.001
**Separated**	880 (0.7)	583 (0.8)	297 (0.6)	205 (0.6)	210 (0.6)	R[4]
**Unable to determine**	1466 (1.2)	1015 (1.4)	451 (1)	385 (1.1)	342 (1)	R[4]
**Widowed**	42383 (35.2)	25850 (34.6)	16533 (36.2)	12594 (35.5)	12753 (35.9)	R[4]
**SEIFA index of relative socioeconomic disadvantage quintile, N (%)**						0.010
**1 – disadvantaged**	24300 (20.2)	14684 (19.7)	9616 (21)	8046 (22.7)	7474 (21.1)	
**2**	25079 (20.9)	14981 (20.1)	10098 (22.1)	7596 (21.4)	7842 (22.1)	
**3**	24877 (20.7)	14981 (20.1)	9896 (21.7)	7326 (20.6)	7664 (21.6)	
**4**	21706 (18.1)	13260 (17.8)	8446 (18.5)	6086 (17.1)	6532 (18.4)	
**5 – advantaged**	24196 (20.1)	16563 (22.2)	7633 (16.7)	6443 (18.2)	5986 (16.9)	
**SEIFA index of education and occupation quintiles, N (%)**						0.013
**1 – Low**	25578 (21.3)	15299 (20.5)	10279 (22.5)	8338 (23.5)	7900 (22.3)	
**2**	23026 (19.2)	13848 (18.6)	9178 (20.1)	7189 (20.3)	7179 (20.2)	
**3**	22916 (19.1)	13624 (18.3)	9292 (20.3)	6844 (19.3)	7237 (20.4)	
**4**	20308 (16.9)	12439 (16.7)	7869 (17.2)	5780 (16.3)	6160 (17.4)	
**5 – High**	28330 (23.6)	19259 (25.9)	9071 (19.9)	7347 (20.7)	7022 (19.8)	
**Select health conditions, N (%)**						
**Dementia**	23686 (19.7)	15667 (20.9)	8019 (17.5)	6057 (17.1)	6070 (17.1)	-0.001
**History of pressure injuries**	5921 (4.9)	3608 (4.8)	2313 (5.1)	1646 (4.6)	1613 (4.5)	-0.001
**Incontinence**	16119 (13.4)	9545 (12.8)	6574 (14.4)	4788 (13.5)	4669 (13.2)	-0.002
**History of cancer**	22187 (18.4)	13812 (18.5)	8375 (18.3)	6297 (17.7)	6185 (17.4)	-0.002
**History of pain**	54780 (45.5)	33188 (44.4)	21592 (47.2)	16783 (47.3)	16479 (46.4)	-0.007
**Chronic respiratory disease**	35738 (29.7)	21544 (28.8)	14194 (31)	11054 (31.1)	10981 (30.9)	-0.001
**Heart failure**	25598 (21.2)	14869 (19.9)	10729 (23.5)	8381 (23.6)	8281 (23.3)	-0.003
**Depression**	49088 (40.7)	30624 (40.9)	18464 (40.4)	14475 (40.8)	14192 (40)	-0.006
**Diabetes**	32340 (26.8)	17309 (23.1)	15031 (32.9)	11804 (33.3)	11522 (32.5)	-0.008
**Ischemic heart disease / hypertension**	85856 (71.2)	51759 (69.2)	34097 (74.6)	26303 (74.1)	26172 (73.7)	-0.002
**Osteoporosis**	34079 (28.3)	20278 (27.1)	13801 (30.2)	10493 (29.6)	10422 (29.4)	-0.002
**Mean number of additional health conditions (SD)**[3]	2.5 (1.6)	2.4 (1.6)	2.6 (1.5)	2.7 (1.6)	2.6 (1.5)	-0.020
						0.006

**Care level at entry, N (%)**						
**Level 1**	18455 (15.3)	11081 (14.8)	7374 (16.1)	7337 (20.7)	6753 (19)	
**Level 2**	74253 (61.6)	44911 (60.1)	29342 (64.2)	21075 (59.4)	22113 (62.3)	
**Level 3**	16644 (13.8)	11108 (14.9)	5536 (12.1)	4875 (13.7)	4534 (12.8)	
**Level 4**	11170 (9.3)	7689 (10.3)	3481 (7.6)	2212 (6.2)	2098 (5.9)	
**Maximally approved care level, N (%)**						0.004
**No approved level**	11992 (10)	7412 (9.9)	4580 (10)	2842 (8)	2627 (7.4)	
**Level 1**	2460 (2)	1498 (2)	962 (2.1)	975 (2.7)	897 (2.5)	
**Level 2**	46302 (38.5)	27174 (36.4)	19128 (41.9)	13940 (39.3)	14580 (41.1)	
**Level 3**	21391 (17.8)	13469 (18)	7922 (17.3)	7497 (21.1)	7284 (20.5)	
**Level 4**	38273 (31.8)	25174 (33.7)	13099 (28.7)	10245 (28.9)	10110 (28.5)	
**Approval for long-term care facility, N (%)**	86023 (71.4)	54051 (72.3)	31972 (69.9)	25124 (70.8)	25203 (71)	
**Approval for respite care, N (%)**	108156 (89.7)	67021 (89.6)	41135 (89.9)	32062 (90.3)	32060 (90.3)	
**Wait time, logarithmized median [IQR]**	5.9 [5.2, 6.4]	5.9 [5.1, 6.4]	5.9 [5.3, 6.4]	5.7 [5, 6.2]	5.7 [5, 6.2]	
**Facility Characteristics**						
**State of service, N (%)**						
**Australian Capital Territory**	1691 (1.4)	1474 (2)	217 (0.5)	173 (0.5)	164 (0.5)	<0.001
**New South Wales**	41972 (34.8)	26317 (35.2)	15655 (34.2)	12113 (34.1)	12027 (33.9)	R
**Northern Territory**	278 (0.2)	232 (0.3)	46 (0.1)	48 (0.1)	43 (0.1)	<0.001
**Queensland**	25578 (21.2)	16510 (22.1)	9068 (19.8)	6678 (18.8)	6765 (19.1)	0.001
**South Australia**	9366 (7.8)	5131 (6.9)	4235 (9.3)	3122 (8.8)	3264 (9.2)	0.004
**Tasmania**	2791 (2.3)	1670 (2.2)	1121 (2.5)	794 (2.2)	832 (2.3)	0.001
**Victoria**	27637 (22.9)	16266 (21.7)	11371 (24.9)	9587 (27)	9459 (26.6)	-0.003
**Western Australia**	11209 (9.3)	7189 (9.6)	4020 (8.8)	2982 (8.4)	2944 (8.3)	<0.001
**Remoteness, N (%)**						
**Major Cities**	82701 (68.6)	51373 (68.7)	31328 (68.5)	24571 (69.2)	24539 (69.1)	R
**Inner Regional**	29365 (24.4)	17438 (23.3)	11927 (26.1)	9078 (25.6)	9116 (25.7)	0.001
**Outer Regional**	7756 (6.4)	5343 (7.1)	2413 (5.3)	1791 (5)	1791 (5)	<0.001
**Remote**	531 (0.4)	478 (0.6)	53 (0.1)	51 (0.1)	44 (0.1)	<-0.001
**Very Remote**	133 (0.1)	*	*	8 (0)	8 (0)	-[5]
**Missing**	36 (0)	*	*	0 (0)	0 (0)	
**Ownership, N (%)**						
**Government**	7881 (6.5)	4818 (6.4)	3063 (6.7)	2386 (6.7)	2391 (6.7)	<0.001
**Not-for-profit**	85721 (71.1)	53456 (71.5)	32265 (70.6)	24882 (70.1)	24994 (70.4)	R
**Private**	26920 (22.3)	16515 (22.1)	10405 (22.8)	8230 (23.2)	8113 (22.9)	-0.005
**History of Podiatry one year prior to entry, N (%)**		22291 (19.8)	39391 (86.1)	30541 (86)	30569 (86.1)	0.001

R=Reference. SD=Standard deviation. SEIFA=Socio-Economic Indexes for Areas.

1. Fine stratification weighted with 50 strata and weights targeting the average treatment effect.
2. Standardised mean differences <0.10 are considered good balance.
3. Excludes health conditions included in the table.
4. Combined reference category.
5. Very remote and remote categories were collapsed for the purpose of weighting due to small cell numbers in very remote categories.

Supplementary Table 9. Baseline Characteristics of Study Cohort by Optometrical Service Utilization Before and After Weighting

		Overall Cohort		*Weighted Cohort[1]*		
**Exposure Status**	**Overall Cohort**	**No Optometrical Services**	**Optometrical Services**	**No Optometrical Services**	**Optometrical Services**	**Standardised Mean Differences[2]**
**Total, N / Sum of Weights**	120522	83113	37409	29030	29030	
**Recipient characteristics**						
**Male sex, N (%)**	47007 (39)	32851 (39.5)	14156 (37.8)	11101 (38.2)	11064 (38.1)	-0.002
**Age at entry, Mean (SD), years**	81.8 (7.3)	82.1 (7.4)	81.2 (7)	81 (7.3)	81.1 (7)	0.005
**Care level at entry, N (%)**						-0.001
**LEVEL 1**	18455 (15.3)	12124 (14.6)	6331 (16.9)	6205 (21.4)	5786 (19.9)	
**LEVEL 2**	74253 (61.6)	49926 (60.1)	24327 (65)	17578 (60.5)	18323 (63.1)	
**LEVEL 3**	16644 (13.8)	12551 (15.1)	4093 (10.9)	3612 (12.4)	3359 (11.6)	
**LEVEL 4**	11170 (9.3)	8512 (10.2)	2658 (7.1)	1636 (5.6)	1562 (5.4)	
**Maximally approved care level, N (%)**						-0.001
**No approved level**	11992 (10)	11521 (9.9)	471 (10.8)	2069 (7.1)	1947 (6.7)	
**LEVEL 1**	2460 (2)	2407 (2.1)	53 (1.2)	984 (3.4)	808 (2.8)	
**LEVEL 2**	46302 (38.5)	44492 (38.3)	1810 (41.4)	12621 (43.5)	13129 (45.2)	
**LEVEL 3**	21391 (17.8)	20716 (17.9)	675 (15.5)	5741 (19.8)	5664 (19.5)	
**LEVEL 4**	38273 (31.8)	36915 (31.8)	1358 (31.1)	7615 (26.2)	7482 (25.8)	
**Approval for long-term care facility, N (%)**	86023 (71.4)	60773 (73.1)	25250 (67.5)	19880 (68.5)	19877 (68.5)	-0.001
**Approval for respite care, N (%)**	108156 (89.7)	75136 (90.4)	33020 (88.3)	25700 (88.5)	25713 (88.6)	0.000
**Wait time, logarithmized median [IQR]**	5.9 [5.2, 6.4]	5.9 [5.2, 6.4]	5.8 [5.1, 6.4]	5.7 [4.8, 6.2]	5.7 [4.7, 6.2]	-0.005
**Culturally and linguistical diverse, N (%)**	15953 (13.2)	12048 (14.5)	3905 (10.4)	3074 (10.6)	3093 (10.7)	0.001
**Partner status, N (%)**						
**Divorced**	10750 (8.9)	7313 (8.8)	3437 (9.2)	2738 (9.4)	2714 (9.3)	<0.001
**Married**	58049 (48.2)	39676 (47.7)	18373 (49.1)	14300 (49.3)	14292 (49.2)	0.001
**Never married**	6994 (5.8)	4858 (5.8)	2136 (5.7)	1629 (5.6)	1616 (5.6)	<0.001
**Separated**	880 (0.7)	593 (0.7)	287 (0.8)	196 (0.7)	204 (0.7)	R[4]
**Unable to determine**	1466 (1.2)	1052 (1.3)	414 (1.1)	325 (1.1)	316 (1.1)	R[4]
**Widowed**	42383 (35.2)	29621 (35.6)	12762 (34.1)	9841 (33.9)	9888 (34.1)	R[4]
**Select health conditions, N (%)**						
**Dementia**	23686 (19.7)	18566 (22.3)	5120 (13.7)	3879 (13.4)	3912 (13.5)	0.001
**History of pressure injuries**	5921 (4.9)	4211 (5.1)	1710 (4.6)	1196 (4.1)	1172 (4)	<0.001
**Incontinence**	16119 (13.4)	11092 (13.3)	5027 (13.4)	3609 (12.4)	3603 (12.4)	<0.001
**History of cancer**	22187 (18.4)	15354 (18.5)	6833 (18.3)	5059 (17.4)	5021 (17.3)	-0.001
**History of pain**	54780 (45.5)	36362 (43.8)	18418 (49.2)	14053 (48.4)	14018 (48.3)	0.001
**Chronic respiratory disease**	35738 (29.7)	24035 (28.9)	11703 (31.3)	9011 (31)	8964 (30.9)	-0.002
**Heart failure**	25598 (21.2)	17760 (21.4)	7838 (21)	6077 (20.9)	6059 (20.9)	<0.001
**Depression**	49088 (40.7)	33429 (40.2)	15659 (41.9)	12012 (41.4)	11955 (41.2)	-0.002
**Diabetes**	32340 (26.8)	21717 (26.1)	10623 (28.4)	8146 (28.1)	8172 (28.2)	0.002
**Ischemic heart disease / hypertension, N (%)**	85856 (71.2)	59018 (71)	26838 (71.7)	20553 (70.8)	20536 (70.7)	-0.001
**Osteoporosis**	34079 (28.3)	23083 (27.8)	10996 (29.4)	8303 (28.6)	8312 (28.6)	0.001

**Mean number of additional health conditions (SD)[3]**	2.5 (1.6)	2.4 (1.6)	2.6 (1.6)	2.6 (1.6)	2.6 (1.6)	0.002
**Facility Characteristics**						
**State of service, N (%)**						
**Australian Capital Territory**	1691 (1.4)	1184 (1.4)	507 (1.4)	366 (1.3)	361 (1.2)	<0.001
**New South Wales**	41972 (34.8)	29272 (35.2)	12700 (33.9)	9752 (33.6)	9827 (33.9)	R
**Northern Territory**	278 (0.2)	196 (0.2)	82 (0.2)	58 (0.2)	55 (0.2)	<0.001
**Queensland**	25578 (21.2)	16767 (20.2)	8811 (23.6)	6614 (22.8)	6610 (22.8)	0.001
**South Australia**	9366 (7.8)	6395 (7.7)	2971 (7.9)	2294 (7.9)	2271 (7.8)	-0.001
**Tasmania**	2791 (2.3)	1871 (2.3)	920 (2.5)	682 (2.4)	666 (2.3)	-0.001
**Victoria**	27637 (22.9)	19118 (23)	8519 (22.8)	7097 (24.4)	7102 (24.5)	<0.001
**Western Australia**	11209 (9.3)	8310 (10)	2899 (7.7)	2165 (7.5)	2138 (7.4)	-0.002
**Remoteness, N (%)**						
**Major Cities**	82701 (68.6)	58651 (70.6)	24050 (64.3)	18961 (65.3)	18897 (65.1)	R
**Inner Regional**	29365 (24.4)	18850 (22.7)	10515 (28.1)	7939 (27.3)	8005 (27.6)	0.003
**Outer Regional**	7756 (6.4)	5135 (6.2)	2621 (7)	1985 (6.8)	1984 (6.8)	<0.001
**Remote**	531 (0.4)	363 (0.4)	168 (0.4)	114 (0.4)	120 (0.4)	<0.001
**Very Remote**	133 (0.1)	94 (0.1)	39 (0.1)	30 (0.1)	24 (0.1)	-[5]
**Missing**	36 (0)	20 (0)	16 (0)	0 (0)	0 (0)	
**Ownership, N (%)**						
**Government**	7881 (6.5)	5185 (6.2)	2696 (7.2)	2107 (7.3)	2120 (7.3)	0.001
**Not-for-profit**	85721 (71.1)	59111 (71.1)	26610 (71.1)	20565 (70.8)	20581 (70.9)	R
**Private**	26920 (22.3)	18817 (22.6)	8103 (21.7)	6359 (21.9)	6329 (21.8)	<0.001
**SEIFA index of relative socioeconomic disadvantage quintile, N (%)**						-0.004
**1 – disadvantaged**	24300 (20.2)	16363 (19.8)	7937 (21.3)	6301 (21.7)	6155 (21.2)	
**2**	25079 (20.9)	16746 (20.2)	8333 (22.3)	6347 (21.9)	6523 (22.5)	
**3**	24877 (20.7)	16934 (20.4)	7943 (21.3)	6081 (20.9)	6151 (21.2)	
**4**	21706 (18.1)	15189 (18.3)	6517 (17.5)	5023 (17.3)	5041 (17.4)	
**5 – advantaged**	24196 (20.1)	17583 (21.2)	6613 (17.7)	5278 (18.2)	5160 (17.8)	
**SEIFA index of education and occupation quintiles, N (%)**						-0.002
**1 – Low**	25578 (21.3)	17096 (20.6)	8482 (22.7)	6711 (23.1)	6523 (22.5)	
**2**	23026 (19.2)	15542 (18.8)	7484 (20)	5808 (20)	5865 (20.2)	
**3**	22916 (19.1)	15275 (18.4)	7641 (20.5)	5658 (19.5)	5971 (20.6)	
**4**	20308 (16.9)	14196 (17.1)	6112 (16.4)	4745 (16.3)	4755 (16.4)	
**5 – High**	28330 (23.6)	20706 (25)	7624 (20.4)	6109 (21)	5916 (20.4)	
**History of Optometrical Services one year prior to entry, N (%)**		31150 (37.5)	20638 (55.2)	16028 (55.2)	16050 (55.3)	-0.001

R=Reference. SD=Standard deviation. SEIFA=Socio-Economic Indexes for Areas.

1. Fine stratification weighted with 50 strata and weights targeting the average treatment effect.
2. Standardised mean differences <0.10 are considered good balance.
3. Excludes health conditions included in the table.
4. Combined reference category.
5. Very remote and remote categories were collapsed for the purpose of weighting due to small cell numbers in very remote categories.

Supplementary Table 10. Baseline Characteristics of Study Cohort by GP Mental Health Services Utilization Before and After Matching

		Overall Cohort			*Matched Cohort*[1]	
**Exposure Status**	**Overall Cohort**	**No GP Mental Health Services**	**GP Mental Health Services**	**No GP Mental Health Services**	**GP Mental Health Services**	**Standardised Mean Differences**[2]
**Total, N / Sum of Weights**	120522	113251	7271	15235	7206	
**Recipient characteristics**						
**Male sex, N (%)**	47007 (39)	32851 (39.5)	14156 (37.8)	11101 (38.2)	11064 (38.1)	-0.002
**Age at entry, Mean (SD), years**	81.8 (7.3)	82.1 (7.4)	81.2 (7)	81 (7.3)	81.1 (7)	0.005
**Care level at entry, N (%)**						-0.001
**LEVEL 1**	18455 (15.3)	12124 (14.6)	6331 (16.9)	6205 (21.4)	5786 (19.9)	
**LEVEL 2**	74253 (61.6)	49926 (60.1)	24327 (65)	17578 (60.5)	18323 (63.1)	
**LEVEL 3**	16644 (13.8)	12551 (15.1)	4093 (10.9)	3612 (12.4)	3359 (11.6)	
**LEVEL 4**	11170 (9.3)	8512 (10.2)	2658 (7.1)	1636 (5.6)	1562 (5.4)	
**Maximally approved care level, N (%)**						-0.001
**No approved level**	11992 (10)	11521 (9.9)	471 (10.8)	2069 (7.1)	1947 (6.7)	
**LEVEL 1**	2460 (2)	2407 (2.1)	53 (1.2)	984 (3.4)	808 (2.8)	
**LEVEL 2**	46302 (38.5)	44492 (38.3)	1810 (41.4)	12621 (43.5)	13129 (45.2)	
**LEVEL 3**	21391 (17.8)	20716 (17.9)	675 (15.5)	5741 (19.8)	5664 (19.5)	
**LEVEL 4**	38273 (31.8)	36915 (31.8)	1358 (31.1)	7615 (26.2)	7482 (25.8)	
**Approval for long-term care facility, N (%)**	86023 (71.4)	60773 (73.1)	25250 (67.5)	19880 (68.5)	19877 (68.5)	-0.001
**Approval for respite care, N (%)**	108156 (89.7)	75136 (90.4)	33020 (88.3)	25700 (88.5)	25713 (88.6)	0.000
**Wait time, logarithmized median [IQR]**	5.9 [5.2, 6.4]	5.9 [5.2, 6.4]	5.8 [5.1, 6.4]	5.7 [4.8, 6.2]	5.7 [4.7, 6.2]	-0.005
**Culturally and linguistical diverse, N (%)**	15953 (13.2)	12048 (14.5)	3905 (10.4)	3074 (10.6)	3093 (10.7)	0.001
**Partner status, N (%)**						
**Divorced**	10750 (8.9)	7313 (8.8)	3437 (9.2)	2738 (9.4)	2714 (9.3)	<0.001
**Married**	58049 (48.2)	39676 (47.7)	18373 (49.1)	14300 (49.3)	14292 (49.2)	0.001
**Never married**	6994 (5.8)	4858 (5.8)	2136 (5.7)	1629 (5.6)	1616 (5.6)	<0.001
**Separated**	880 (0.7)	593 (0.7)	287 (0.8)	196 (0.7)	204 (0.7)	R[4]
**Unable to determine**	1466 (1.2)	1052 (1.3)	414 (1.1)	325 (1.1)	316 (1.1)	R[4]
**Widowed**	42383 (35.2)	29621 (35.6)	12762 (34.1)	9841 (33.9)	9888 (34.1)	R[4]
**Mean number of additional health conditions (SD)**[3]	2.5 (1.6)	2.4 (1.6)	2.6 (1.6)	2.6 (1.6)	2.6 (1.6)	0.002
**Select health conditions, N (%)**						
**Dementia**	23686 (19.7)	18566 (22.3)	5120 (13.7)	3879 (13.4)	3912 (13.5)	0.001
**History of pressure injuries**	5921 (4.9)	4211 (5.1)	1710 (4.6)	1196 (4.1)	1172 (4)	<0.001
**Incontinence**	16119 (13.4)	11092 (13.3)	5027 (13.4)	3609 (12.4)	3603 (12.4)	<0.001
**History of cancer**	22187 (18.4)	15354 (18.5)	6833 (18.3)	5059 (17.4)	5021 (17.3)	-0.001
**History of pain**	54780 (45.5)	36362 (43.8)	18418 (49.2)	14053 (48.4)	14018 (48.3)	0.001
**History of chronic respiratory disease**	35738 (29.7)	24035 (28.9)	11703 (31.3)	9011 (31)	8964 (30.9)	-0.002
**Heart failure**	25598 (21.2)	17760 (21.4)	7838 (21)	6077 (20.9)	6059 (20.9)	<0.001
**Depression**	49088 (40.7)	33429 (40.2)	15659 (41.9)	12012 (41.4)	11955 (41.2)	-0.002
**Diabetes**	32340 (26.8)	21717 (26.1)	10623 (28.4)	8146 (28.1)	8172 (28.2)	0.002
**Ischemic heart disease / hypertension**	85856 (71.2)	59018 (71)	26838 (71.7)	20553 (70.8)	20536 (70.7)	-0.001

Osteoporosis	34079 (28.3)	23083 (27.8)	10996 (29.4)	8303 (28.6)	8312 (28.6)	0.001
**Facility Characteristics**						
**State of service, N (%)**						
**Australian Capital Territory**	1691 (1.4)	1184 (1.4)	507 (1.4)	366 (1.3)	361 (1.2)	<0.001
**New South Wales**	41972 (34.8)	29272 (35.2)	12700 (33.9)	9752 (33.6)	9827 (33.9)	R
**Northern Territory**	278 (0.2)	196 (0.2)	82 (0.2)	58 (0.2)	55 (0.2)	<0.001
**Queensland**	25578 (21.2)	16767 (20.2)	8811 (23.6)	6614 (22.8)	6610 (22.8)	0.001
**South Australia**	9366 (7.8)	6395 (7.7)	2971 (7.9)	2294 (7.9)	2271 (7.8)	-0.001
**Tasmania**	2791 (2.3)	1871 (2.3)	920 (2.5)	682 (2.4)	666 (2.3)	-0.001
**Victoria**	27637 (22.9)	19118 (23)	8519 (22.8)	7097 (24.4)	7102 (24.5)	<0.001
**Western Australia**	11209 (9.3)	8310 (10)	2899 (7.7)	2165 (7.5)	2138 (7.4)	-0.002
**Remoteness, N (%)**						
**Major Cities**	82701 (68.6)	58651 (70.6)	24050 (64.3)	18961 (65.3)	18897 (65.1)	R
**Inner Regional**	29365 (24.4)	18850 (22.7)	10515 (28.1)	7939 (27.3)	8005 (27.6)	0.003
**Outer Regional**	7756 (6.4)	5135 (6.2)	2621 (7)	1985 (6.8)	1984 (6.8)	<0.001
**Remote**	531 (0.4)	363 (0.4)	168 (0.4)	114 (0.4)	120 (0.4)	<0.001
**Very Remote**	133 (0.1)	94 (0.1)	39 (0.1)	30 (0.1)	24 (0.1)	-[5]
**Missing**	36 (0)	20 (0)	16 (0)	0 (0)	0 (0)	
**Ownership, N (%)**						
**Government**	7881 (6.5)	5185 (6.2)	2696 (7.2)	2107 (7.3)	2120 (7.3)	0.001
**Not-for-profit**	85721 (71.1)	59111 (71.1)	26610 (71.1)	20565 (70.8)	20581 (70.9)	R
**Private**	26920 (22.3)	18817 (22.6)	8103 (21.7)	6359 (21.9)	6329 (21.8)	<0.001
**SEIFA index of relative socioeconomic disadvantage quintile, N (%)**						-0.004
**1 – disadvantaged**	24300 (20.2)	16363 (19.8)	7937 (21.3)	6301 (21.7)	6155 (21.2)	
**2**	25079 (20.9)	16746 (20.2)	8333 (22.3)	6347 (21.9)	6523 (22.5)	
**3**	24877 (20.7)	16934 (20.4)	7943 (21.3)	6081 (20.9)	6151 (21.2)	
**4**	21706 (18.1)	15189 (18.3)	6517 (17.5)	5023 (17.3)	5041 (17.4)	
**5 – advantaged**	24196 (20.1)	17583 (21.2)	6613 (17.7)	5278 (18.2)	5160 (17.8)	
**SEIFA index of education and occupation quintile, N (%)**						-0.002
**1 – Low**	25578 (21.3)	17096 (20.6)	8482 (22.7)	6711 (23.1)	6523 (22.5)	
**2**	23026 (19.2)	15542 (18.8)	7484 (20)	5808 (20)	5865 (20.2)	
**3**	22916 (19.1)	15275 (18.4)	7641 (20.5)	5658 (19.5)	5971 (20.6)	
**4**	20308 (16.9)	14196 (17.1)	6112 (16.4)	4745 (16.3)	4755 (16.4)	
**5 – High**	28330 (23.6)	20706 (25)	7624 (20.4)	6109 (21)	5916 (20.4)	
**History of Optometrical Services one year prior to entry, N (%)**		31150 (37.5)	20638 (55.2)	16028 (55.2)	16050 (55.3)	-0.001

R=Reference. SD=Standard deviation. SEIFA=Socio-Economic Indexes for Areas. SMD=Standardised mean difference.

1. Propensity score matched on the logit of the propensity score with a 3:1 nearest-neighbour matching without replacement and calipers of 0.2 standard deviations of the propensity score logit.
2. Standardised mean differences <0.10 are considered good balance.
3. Excludes health conditions included in the table.
4. Combined reference category.
5. Very remote and remote categories were collapsed for the purpose of matching due to small cell numbers in very remote categories.

Supplementary Table 11. Baseline Characteristics of Study Cohort by Pattern of Service Utilization Exposures Before and After Matching

	Overall Cohort				Matched Cohort 1: Preventive vs Low Use[1]			Matched Cohort 2: Preventive vs High Use[1]		
**Exposure Status**	**Overall Cohort[2]**	**Preventive Use**	**Low Use**	**High Use**	**Preventive Use**	**Low Use**	**SMD[3]**	**Preventive Use**	**High Use**	**SMD[3]**
**Total, N (%)**	54489	34021	16452	4016	16070	16219		3987	3987	
**Individual characteristics**										
**Male sex, N (%)**	20043 (36.8)	12457 (36.6)	6165 (37.5)	1421 (35.4)	6025 (37.5)	6078 (37.5)	<0.001	1402 (35.2)	1408 (35.3)	-0.002
**Age at entry, Mean (SD), years**	81.2 (7.3)	81.12 (7.09)	81.25 (7.59)	80.94 (7.51)	81.2 (7.1)	81.3 (7.6)	-0.005	80.8 (7.1)	81 (7.5)	-0.018
**Care level at entry, N (%)**							-0.020			-0.062
**Level 1**	5610 (10.3)	3778 (11.1)	1514 (9.2)	318 (7.9)	1439 (9)	1499 (9.2)		344 (8.6)	316 (7.9)	
**Level 2**	37202 (68.3)	23769 (69.9)	10804 (65.7)	2629 (65.5)	10815 (67.3)	10652 (65.7)		2685 (67.3)	2613 (65.5)	
**Level 3**	6499 (11.9)	3686 (10.8)	2229 (13.5)	584 (14.5)	2085 (13)	2197 (13.5)		528 (13.2)	577 (14.5)	
**Level 4**	5178 (9.5)	2788 (8.2)	1905 (11.6)	485 (12.1)	1731 (10.8)	1871 (11.5)		430 (10.8)	481 (12.1)	
**Maximally approved care level, N (%)**							-0.023			-0.035
**No approved level**	5318 (9.8)	3312 (9.8)	1567 (9.5)	439 (11.0)	1465 (9.1)	1547 (9.5)		372 (9.3)	437 (11)	
**Level 1**	386 (0.7)	266 (0.8)	105 (0.6)	15 (0.4)	104 (0.6)	104 (0.6)		25 (0.6)	15 (0.4)	
**Level 2**	23009 (42.2)	15173 (44.7)	6423 (39.1)	1413 (35.2)	6630 (41.3)	6346 (39.1)		1600 (40.1)	1407 (35.3)	
**Level 3**	7046 (12.9)	4492 (13.2)	2032 (12.4)	522 (13.0)	2095 (13)	2007 (12.4)		532 (13.3)	519 (13)	
**Level 4**	18647 (34.2)	10724 (31.6)	6303 (38.4)	1620 (40.4)	5776 (35.9)	6215 (38.3)		1458 (36.6)	1609 (40.4)	
**Approval for long-term care facility, N (%)**	37943 (69.6)	23159 (68.1)	11896 (72.3)	2888 (71.9)	11463 (71.3)	11760 (72.5)	-0.011	2821 (70.8)	2872 (72)	-0.013
**Approval for respite care, N (%)**	48859 (89.7)	30404 (89.4)	14814 (90.0)	3641 (90.7)	14528 (90.4)	14629 (90.2)	0.002	3571 (89.6)	3621 (90.8)	-0.013
**Wait time, logarithmized median [IQR]**	5.8 [4.6, 6.5]	5.79 [4.67, 6.46]	5.76 [4.52, 6.42]	5.77 [4.54, 6.47]	5.8 [4.5, 6.4]	5.8 [4.5, 6.4]	0.004	5.8 [4.5, 6.4]	5.8 [4.5, 6.5]	-0.004
**Culturally and linguistical diverse, N (%)**	7425 (13.6)	4065 (11.9)	2440 (14.8)	920 (22.9)	2352 (14.6)	2423 (14.9)	-0.002	794 (19.9)	915 (22.9)	-0.030
**Partner status, N (%)**										
**Divorced**	4959 (9.1)	2987 (8.8)	1578 (9.6)	394 (9.8)	1496 (9.3)	1562 (9.6)	-0.002	383 (9.6)	392 (9.8)	-0.002
**Married**	25818 (47.4)	16399 (48.2)	7521 (45.7)	1898 (47.3)	7495 (46.6)	7428 (45.8)	0.007	1876 (47.1)	1882 (47.2)	<0.001
**Never married**	3314 (6.1)	1966 (5.8)	1131 (6.9)	217 (5.4)	1044 (6.5)	1092 (6.7)	-0.002	227 (5.7)	214 (5.4)	0.003
**Separated**	450 (0.8)	293 (0.9)	131 (0.8)	26 (0.6)	142 (0.9)	129 (0.8)	R[5]	35 (0.9)	26 (0.7)	R[5]
**Unable to determine**	638 (1.2)	372 (1.1)	225 (1.4)	41 (1.0)	159 (1)	209 (1.3)	R[5]	39 (1)	39 (1)	R[5]
**Widowed**	19310 (35.4)	12004 (35.3)	5866 (35.7)	1440 (35.9)	5734 (35.7)	5799 (35.8)	R[5]	1427 (35.8)	1434 (36)	R[5]
**Mean number of additional health conditions (SD)[4]**	2.52 (1.57)	2.62 (1.55)	2.25 (1.56)	2.82 (1.62)	2.4 (1.5)	2.3 (1.6)	0.052	2.8 (1.6)	2.8 (1.6)	-0.019

**Select health conditions, N (%)**										
**Dementia**	9144 (16.8)	5115 (15.0)	3283 (20.0)	746 (18.6)	3037 (18.9)	3240 (20)	-0.009	713 (17.9)	740 (18.6)	-0.007
**History of pressure injuries**	2572 (4.7)	1570 (4.6)	763 (4.6)	239 (6.0)	733 (4.6)	754 (4.6)	-0.001	204 (5.1)	239 (6)	-0.009
**Incontinence**	7557 (13.9)	4850 (14.3)	2036 (12.4)	671 (16.7)	2063 (12.8)	2007 (12.4)	0.004	655 (16.4)	666 (16.7)	-0.003
**History of cancer**	9214 (16.9)	5892 (17.3)	2640 (16.0)	682 (17.0)	2708 (16.9)	2605 (16.1)	0.007	730 (18.3)	680 (17.1)	0.013
**History of pain**	25631 (47.0)	16648 (48.9)	6836 (41.6)	2147 (53.5)	6971 (43.4)	6774 (41.8)	0.014	2076 (52.1)	2133 (53.5)	-0.014
**Chronic respiratory disease**	16114 (29.6)	10499 (30.9)	4229 (25.7)	1386 (34.5)	4461 (27.8)	4181 (25.8)	0.019	1316 (33)	1375 (34.5)	-0.015
**Heart failure**	11161 (20.5)	7323 (21.5)	2853 (17.3)	985 (24.5)	2966 (18.5)	2823 (17.4)	0.010	943 (23.7)	980 (24.6)	-0.009
**Depression**	22437 (41.2)	14231 (41.8)	6337 (38.5)	1869 (46.5)	6318 (39.3)	6259 (38.6)	0.006	1881 (47.2)	1858 (46.6)	0.006
**Diabetes**	14713 (27.0)	10222 (30.0)	3209 (19.5)	1282 (31.9)	3556 (22.1)	3184 (19.6)	0.023	1263 (31.7)	1275 (32)	-0.003
**Ischemic heart disease / hypertension**	38494 (70.6)	24562 (72.2)	11039 (67.1)	2893 (72.0)	11001 (68.5)	10917 (67.3)	0.010	2875 (72.1)	2873 (72.1)	0.001
**Osteoporosis**	15218 (27.9)	9916 (29.1)	4080 (24.8)	1222 (30.4)	4081 (25.4)	4036 (24.9)	0.004	1158 (29)	1216 (30.5)	-0.015
**Facility Characteristics**										
**State of service, N (%)**										
**Australian Capital Territory**	769 (1.4)	312 (0.9)	429 (2.6)	28 (0.7)	271 (1.7)	412 (2.5)	-0.003	35 (0.9)	28 (0.7)	0.002
**New South Wales**	18500 (34.0)	12028 (35.4)	5246 (31.9)	1226 (30.5)	5249 (32.7)	5167 (31.9)	R	1242 (31.2)	1220 (30.6)	R
**Northern Territory**	114 (0.2)	41 (0.1)	66 (0.4)	7 (0.2)	29 (0.2)	51 (0.3)	-0.001	6 (0.2)	7 (0.2)	<0.001
**Queensland**	11691 (21.5)	7284 (21.4)	3530 (21.5)	877 (21.8)	3492 (21.7)	3499 (21.6)	<0.001	895 (22.4)	873 (21.9)	0.006
**South Australia**	4173 (7.7)	2603 (7.7)	1220 (7.4)	350 (8.7)	1163 (7.2)	1208 (7.4)	-0.002	329 (8.3)	343 (8.6)	-0.004
**Tasmania**	1280 (2.3)	841 (2.5)	383 (2.3)	56 (1.4)	398 (2.5)	379 (2.3)	0.001	59 (1.5)	55 (1.4)	0.001
**Victoria**	12886 (23.6)	7751 (22.8)	3949 (24.0)	1186 (29.5)	3892 (24.2)	3898 (24)	<0.001	1147 (28.8)	1178 (29.5)	-0.008
**Western Australia**	5076 (9.3)	3161 (9.3)	1629 (9.9)	286 (7.1)	1576 (9.8)	1605 (9.9)	-0.001	274 (6.9)	283 (7.1)	-0.002
**Remoteness, N (%)**										
**Major Cities**	36308 (66.6)	21988 (64.6)	11065 (67.3)	3255 (81.1)	10740 (66.8)	10934 (67.4)	R	3110 (78)	3229 (81)	R
**Inner Regional**	14354 (26.3)	9720 (28.6)	4047 (24.6)	587 (14.6)	4142 (25.8)	3991 (24.6)	0.009	681 (17.1)	586 (14.7)	0.024
**Outer Regional**	3519 (6.5)	2199 (6.5)	1166 (7.1)	154 (3.8)	1096 (6.8)	1148 (7.1)	-0.003	179 (4.5)	152 (3.8)	0.007
**Remote**	221 (0.4)	80 (0.2)	128 (0.8)	13 (0.3)	69 (0.4)	113 (0.7)	-0.001	10 (0.3)	13 (0.3)	-0.001
**Very Remote**	67 (0.1)	26 (0.1)	34 (0.2)	7 (0.2)	23 (0.1)	33 (0.2)	R[6]	*	*	R[6]
**Missing**	20 (0.1)	8 (0.0)	12 (0.1)	0 (0.0)	0 (0)	0 (0)		*	*	
**Ownership, N (%)**										
**Government**	3693 (6.8)	2215 (6.5)	1244 (7.6)	234 (5.8)	1172 (7.3)	1228 (7.6)	-0.002	249 (6.2)	230 (5.8)	0.005
**Not-for-profit**	40030 (73.5)	24878 (73.1)	12205 (74.2)	2947 (73.4)	11877 (73.9)	12040 (74.2)	R	2928 (73.4)	2926 (73.4)	R

Private	10766 (19.8)	6928 (20.4)	3003 (18.3)	835 (20.8)	3021 (18.8)	2951 (18.2)	0.005	810 (20.3)	831 (20.8)	-0.005
**SEIFA index of relative socioeconomic disadvantage quintile, N (%)**							-0.032			-0.004
1 – disadvantaged	11093 (20.4)	7051 (20.7)	3258 (19.9)	784 (19.5)	2986 (18.6)	3231 (19.9)		662 (16.6)	780 (19.6)	
2	11488 (21.1)	7650 (22.5)	3168 (19.4)	670 (16.7)	3329 (20.7)	3143 (19.4)		754 (18.9)	665 (16.7)	
3	11170 (20.5)	7391 (21.7)	2999 (18.3)	780 (19.4)	3421 (21.3)	2966 (18.3)		883 (22.1)	777 (19.5)	
4	9874 (18.2)	6039 (17.8)	3011 (18.4)	824 (20.5)	3053 (19)	2981 (18.4)		829 (20.8)	813 (20.4)	
5 – advantaged	10747 (19.8)	5859 (17.2)	3934 (24.0)	954 (23.8)	3281 (20.4)	3898 (24)		859 (21.5)	952 (23.9)	
**SEIFA index of education and occupation quintiles, N (%)**							-0.035			-0.024
1 – Low	11676 (21.5)	7546 (22.2)	3358 (20.5)	772 (19.2)	3094 (19.3)	3329 (20.5)		696 (17.5)	770 (19.3)	
2	10392 (19.1)	6922 (20.4)	2857 (17.5)	613 (15.3)	3000 (18.7)	2832 (17.5)		674 (16.9)	610 (15.3)	
3	10369 (19.1)	6957 (20.5)	2726 (16.7)	686 (17.1)	3221 (20)	2702 (16.7)		819 (20.5)	681 (17.1)	
4	9238 (17.0)	5662 (16.7)	2787 (17.0)	789 (19.7)	2830 (17.6)	2759 (17)		746 (18.7)	779 (19.5)	
5 – High	12697 (23.4)	6903 (20.3)	4642 (28.4)	1152 (28.7)	3925 (24.4)	4597 (28.3)		1052 (26.4)	1147 (28.8)	

R=Reference. SD=Standard deviation. SEIFA=Socio-Economic Indexes for Areas. SMD=Standardised mean difference.

1. Propensity score matched on the logit of the propensity score with a 3:1 nearest-neighbour matching without replacement and calipers of 0.2 standard deviations of the propensity score logit.
2. Individuals alive at 12 months after care entry.
3. Standardised mean differences <0.10 are considered good balance.
4. Excludes health conditions included in the table.
5. Combined reference category.
6. Very remote and remote categories were collapsed for the purpose of matching due to small cell numbers in very remote categories.

Supplementary Table 12. Crude Health Outcome Event Rate and Cumulative Incidence within One Year by Patterns of Health Care Service Utilization and Specific Primary Health Care Services Exposures

Exposure	Outcome	Exposure Status	Events (N)	Individuals (N)	Person-days	Event rate 100 Recipient-years	Cumulative Incidence within 1 year, % (95CI)
GP attendances after-hours	Premature mortality	Unexposed	195	79973	23685307	0.3	0.4 (0.3, 0.4)
	Premature mortality	Exposed	83	23526	6611927	0.5	0.6 (0.4, 0.7)
	Mortality	Unexposed	4469	79973	23685307	6.9	7.9 (7.7, 8.2)
	Mortality	Exposed	2193	23526	6611927	12.1	13.5 (13.0, 14.1)
	Emergency department presentations	Unexposed	26479	69033	20427263	47.3	41.1 (40.7, 41.4)
	Emergency department presentations	Exposed	9455	21223	5958656	57.9	53.7 (52.9, 54.4)
	Potentially preventable hospitalizations	Unexposed	10589	69033	20427263	18.9	17.0 (16.7, 17.3)
	Potentially preventable hospitalizations	Exposed	4254	21223	5958656	26.1	25.3 (24.6, 26.0)
	Unplanned hospitalizations	Unexposed	23726	69033	20427263	42.4	36.8 (36.4, 37.1)
	Unplanned hospitalizations	Exposed	9203	21223	5958656	56.4	51.7 (50.9, 52.4)
	Fall-related hospitalizations	Unexposed	7505	69033	20427263	13.4	12.1 (11.9, 12.4)
	Fall-related hospitalizations	Exposed	2758	21223	5958656	16.9	16.9 (16.3, 17.4)
	Fracture-related hospitalizations	Unexposed	3340	69033	20427263	6.0	5.4 (5.3, 5.6)
	Fracture-related hospitalizations	Exposed	1181	21223	5958656	7.2	7.3 (6.9, 7.7)
	Medication-related adverse events	Unexposed	2184	69033	20427263	3.9	3.6 (3.4, 3.7)
	Medication-related adverse events	Exposed	945	21223	5958656	5.8	5.9 (5.5, 6.2)
	Pressure injury hospitalizations	Unexposed	2406	69033	20427263	4.3	3.9 (3.8, 4.1)
	Pressure injury hospitalizations	Exposed	1166	21223	5958656	7.1	7.1 (6.7, 7.5)
	Malnutrition hospitalizations	Unexposed	3350	69033	20427263	6.0	5.4 (5.2, 5.6)
	Malnutrition hospitalizations	Exposed	1384	21223	5958656	8.5	8.5 (8.0, 8.9)
	Dementia hospitalizations	Unexposed	1069	11918	3429648	11.4	10.8 (10.1, 11.4)
	Dementia hospitalizations	Exposed	515	4580	1209021	15.5	16.5 (15.2, 17.9)
Urgent GP attendances after-hours	Premature mortality	Unexposed	212	89302	26959035	0.3	0.4 (0.3, 0.4)
	Premature mortality	Exposed	51	10534	2977874	0.6	0.7 (0.5, 0.9)

	Mortality	Unexposed	4739	89302	26959035	6.4	7.7 (7.5, 7.9)
	Mortality	Exposed	1530	10534	2977874	18.8	19.7 (18.7, 20.6)
	Emergency department presentations	Unexposed	29939	77744	23452387	46.6	41.2 (40.9, 41.6)
	Emergency department presentations	Exposed	5137	9304	2620931	71.5	66.1 (65.0, 67.2)
	Potentially preventable hospitalizations	Unexposed	12017	77744	23452387	18.7	17.1 (16.8, 17.4)
	Potentially preventable hospitalizations	Exposed	2489	9304	2620931	34.7	34.4 (33.3, 35.6)
	Unplanned hospitalizations	Unexposed	26995	77744	23452387	42.0	37.1 (36.8, 37.5)
	Unplanned hospitalizations	Exposed	5115	9304	2620931	71.2	65.3 (64.1, 66.4)
	Fall-related hospitalizations	Unexposed	8560	77744	23452387	13.3	12.3 (12.0, 12.5)
	Fall-related hospitalizations	Exposed	1481	9304	2620931	20.6	21.4 (20.4, 22.4)
	Fracture-related hospitalizations	Unexposed	3715	77744	23452387	5.8	5.4 (5.2, 5.6)
	Fracture-related hospitalizations	Exposed	704	9304	2620931	9.8	10.2 (9.5, 11.0)
	Medication-related adverse events	Unexposed	2482	77744	23452387	3.9	3.6 (3.5, 3.7)
	Medication-related adverse events	Exposed	592	9304	2620931	8.2	8.8 (8.1, 9.5)
	Pressure injury hospitalizations	Unexposed	2662	77744	23452387	4.1	3.8 (3.7, 4.0)
	Pressure injury hospitalizations	Exposed	854	9304	2620931	11.9	12.1 (11.4, 12.9)
	Malnutrition hospitalizations	Unexposed	3725	77744	23452387	5.8	5.4 (5.2, 5.5)
	Malnutrition hospitalizations	Exposed	924	9304	2620931	12.9	13.0 (12.2, 13.8)
	Dementia hospitalizations	Unexposed	1265	13549	3998150	11.5	11.3 (10.7, 11.9)
	Dementia hospitalizations	Exposed	308	2124	562951	20.0	21.8 (19.5, 24.1)
Health assessments	Premature mortality	Unexposed	153	51681	15669795	0.4	0.5 (0.4, 0.5)
	Premature mortality	Exposed	67	26184	8133449	0.3	0.4 (0.3, 0.5)
	Mortality	Unexposed	3505	51681	15669795	8.2	10.1 (9.8, 10.4)
	Mortality	Exposed	1402	26184	8133449	6.3	7.7 (7.3, 8.1)
	Emergency department presentations	Unexposed	17505	44659	13530751	47.2	42.7 (42.2, 43.2)
	Emergency department presentations	Exposed	9479	23084	7160595	48.3	44.8 (44.2, 45.5)
	Potentially preventable hospitalizations	Unexposed	7124	44659	13530751	19.2	18.1 (17.7, 18.4)
	Potentially preventable hospitalizations	Exposed	3797	23084	7160595	19.4	18.7 (18.2, 19.3)
	Unplanned hospitalizations	Unexposed	16415	44659	13530751	44.3	39.9 (39.4, 40.4)

	Unplanned hospitalizations	Exposed	8578	23084	7160595	43.7	40.6 (39.9, 41.2)
	Fall-related hospitalizations	Unexposed	5363	44659	13530751	14.5	13.7 (13.4, 14.1)
	Fall-related hospitalizations	Exposed	2888	23084	7160595	14.7	14.3 (13.8, 14.8)
	Fracture-related hospitalizations	Unexposed	2340	44659	13530751	6.3	6.1 (5.8, 6.3)
	Fracture-related hospitalizations	Exposed	1207	23084	7160595	6.2	6.0 (5.7, 6.4)
	Medication-related adverse events	Unexposed	1442	44659	13530751	3.9	3.8 (3.6, 3.9)
	Medication-related adverse events	Exposed	815	23084	7160595	4.2	4.1 (3.8, 4.4)
	Pressure injury hospitalizations	Unexposed	1875	44659	13530751	5.1	4.8 (4.6, 5.0)
	Pressure injury hospitalizations	Exposed	778	23084	7160595	4.0	3.9 (3.6, 4.2)
	Malnutrition hospitalizations	Unexposed	2456	44659	13530751	6.6	6.3 (6.1, 6.5)
	Malnutrition hospitalizations	Exposed	1152	23084	7160595	5.9	5.8 (5.4, 6.1)
	Dementia hospitalizations	Unexposed	902	8618	2574317	12.8	12.9 (12.1, 13.7)
	Dementia hospitalizations	Exposed	384	4095	1212670	11.6	12.0 (10.9, 13.2)
Management plans	Premature mortality	Unexposed	148	46541	12526121	0.4	0.5 (0.4, 0.6)
	Premature mortality	Exposed	145	58931	17977756	0.3	0.3 (0.3, 0.4)
	Mortality	Unexposed	3461	46541	12526121	10.1	11.3 (10.9, 11.7)
	Mortality	Exposed	3396	58931	17977756	6.9	7.9 (7.6, 8.2)
	Emergency department presentations	Unexposed	15043	40103	10772553	51.0	41.6 (41.1, 42.2)
	Emergency department presentations	Exposed	22504	51721	15781613	52.0	46.5 (46.1, 47.0)
	Potentially preventable hospitalizations	Unexposed	5974	40103	10772553	20.2	17.3 (16.9, 17.7)
	Potentially preventable hospitalizations	Exposed	9499	51721	15781613	22.0	20.4 (20.0, 20.8)
	Unplanned hospitalizations	Unexposed	14053	40103	10772553	47.6	38.8 (38.3, 39.3)
	Unplanned hospitalizations	Exposed	20405	51721	15781613	47.2	42.1 (41.7, 42.6)
	Fall-related hospitalizations	Unexposed	4426	40103	10772553	15.0	13.0 (12.6, 13.3)
	Fall-related hospitalizations	Exposed	6462	51721	15781613	14.9	14.0 (13.6, 14.3)
	Fracture-related hospitalizations	Unexposed	2013	40103	10772553	6.8	6.0 (5.7, 6.2)
	Fracture-related hospitalizations	Exposed	2794	51721	15781613	6.5	6.1 (5.9, 6.3)
	Medication-related adverse events	Unexposed	1242	40103	10772553	4.2	3.7 (3.5, 3.9)
	Medication-related adverse events	Exposed	2070	51721	15781613	4.8	4.5 (4.3, 4.7)

	Pressure injury hospitalizations	Unexposed	1759	40103	10772553	6.0	5.2 (4.9, 5.4)
	Pressure injury hospitalizations	Exposed	2014	51721	15781613	4.7	4.4 (4.2, 4.6)
	Malnutrition hospitalizations	Unexposed	2296	40103	10772553	7.8	6.7 (6.5, 7.0)
	Malnutrition hospitalizations	Exposed	2765	51721	15781613	6.4	6.0 (5.8, 6.2)
	Dementia hospitalizations	Unexposed	861	8171	2146372	14.6	13.3 (12.5, 14.2)
	Dementia hospitalizations	Exposed	897	8665	2531196	12.9	12.6 (11.8, 13.4)
Allied Health - Podiatry	Premature mortality	Unexposed	199	67186	17800340	0.4	0.4 (0.4, 0.5)
	Premature mortality	Exposed	110	45377	13178665	0.3	0.3 (0.3, 0.4)
	Mortality	Unexposed	5113	67186	17800340	10.5	10.5 (10.2, 10.8)
	Mortality	Exposed	2491	45377	13178665	6.9	7.5 (7.2, 7.8)
	Emergency department presentations	Unexposed	23994	57849	15329660	57.1	46.0 (45.5, 46.4)
	Emergency department presentations	Exposed	17767	40032	11631005	55.8	47.1 (46.6, 47.6)
	Potentially preventable hospitalizations	Unexposed	9696	57849	15329660	23.1	19.4 (19.1, 19.8)
	Potentially preventable hospitalizations	Exposed	7417	40032	11631005	23.3	20.4 (20.0, 20.9)
	Unplanned hospitalizations	Unexposed	22205	57849	15329660	52.9	42.4 (42.0, 42.9)
	Unplanned hospitalizations	Exposed	16105	40032	11631005	50.5	42.7 (42.2, 43.2)
	Fall-related hospitalizations	Unexposed	6968	57849	15329660	16.6	14.1 (13.8, 14.4)
	Fall-related hospitalizations	Exposed	5238	40032	11631005	16.4	14.6 (14.2, 14.9)
	Fracture-related hospitalizations	Unexposed	3232	57849	15329660	7.7	6.6 (6.4, 6.8)
	Fracture-related hospitalizations	Exposed	2194	40032	11631005	6.9	6.2 (5.9, 6.4)
	Medication-related adverse events	Unexposed	2109	57849	15329660	5.0	4.3 (4.1, 4.5)
	Medication-related adverse events	Exposed	1626	40032	11631005	5.1	4.6 (4.4, 4.8)
	Pressure injury hospitalizations	Unexposed	2701	57849	15329660	6.4	5.5 (5.3, 5.7)
	Pressure injury hospitalizations	Exposed	1501	40032	11631005	4.7	4.2 (4.0, 4.4)
	Malnutrition hospitalizations	Unexposed	3623	57849	15329660	8.6	7.3 (7.1, 7.5)
	Malnutrition hospitalizations	Exposed	2095	40032	11631005	6.6	5.9 (5.6, 6.1)
	Dementia hospitalizations	Unexposed	1231	11533	2888177	15.6	13.4 (12.7, 14.2)
	Dementia hospitalizations	Exposed	750	6886	1896813	14.4	13.1 (12.2, 14.0)
Allied Health - Optometrical Services	Premature mortality	Unexposed	190	62450	18221432	0.4	0.5 (0.4, 0.6)

	Premature mortality	Exposed	75	37080	11687257	0.2	0.3 (0.2, 0.3)
	Mortality	Unexposed	4485	62450	18221432	9.0	10.9 (10.5, 11.2)
	Mortality	Exposed	1581	37080	11687257	4.9	6.0 (5.7, 6.3)
	Emergency department presentations	Unexposed	21254	54001	15734220	49.3	43.0 (42.5, 43.4)
	Emergency department presentations	Exposed	13577	32735	10309860	48.1	43.8 (43.3, 44.4)
	Potentially preventable hospitalizations	Unexposed	8677	54001	15734220	20.1	18.3 (17.9, 18.6)
	Potentially preventable hospitalizations	Exposed	5631	32735	10309860	19.9	18.7 (18.3, 19.2)
	Unplanned hospitalizations	Unexposed	19768	54001	15734220	45.9	39.9 (39.5, 40.3)
	Unplanned hospitalizations	Exposed	12144	32735	10309860	43.0	39.2 (38.7, 39.8)
	Fall-related hospitalizations	Unexposed	6377	54001	15734220	14.8	13.6 (13.3, 13.9)
	Fall-related hospitalizations	Exposed	3730	32735	10309860	13.2	12.5 (12.1, 12.9)
	Fracture-related hospitalizations	Unexposed	2836	54001	15734220	6.6	6.1 (5.9, 6.3)
	Fracture-related hospitalizations	Exposed	1635	32735	10309860	5.8	5.5 (5.3, 5.8)
	Medication-related adverse events	Unexposed	1819	54001	15734220	4.2	3.9 (3.8, 4.1)
	Medication-related adverse events	Exposed	1262	32735	10309860	4.5	4.3 (4.0, 4.5)
	Pressure injury hospitalizations	Unexposed	2463	54001	15734220	5.7	5.3 (5.1, 5.5)
	Pressure injury hospitalizations	Exposed	965	32735	10309860	3.4	3.3 (3.1, 3.5)
	Malnutrition hospitalizations	Unexposed	3099	54001	15734220	7.2	6.6 (6.4, 6.8)
	Malnutrition hospitalizations	Exposed	1528	32735	10309860	5.4	5.2 (4.9, 5.4)
	Dementia hospitalizations	Unexposed	1191	11159	3222885	13.5	13.2 (12.5, 14.0)
	Dementia hospitalizations	Exposed	393	4349	1322709	10.8	10.8 (9.8, 11.9)
Primary care mental health services - collapsed	Premature mortality	Unexposed	28	15235	4395278	0.2	0.3 (0.2, 0.4)
	Premature mortality	Exposed	21	7206	2208457	0.3	0.4 (0.2, 0.6)
	Mortality	Unexposed	846	15235	4395278	7.0	7.6 (7.1, 8.1)
	Mortality	Exposed	306	7206	2208457	5.1	5.7 (5.1, 6.3)
	Emergency department presentations	Unexposed	5727	13642	3935436	53.1	45.0 (44.2, 45.9)
	Emergency department presentations	Exposed	3024	6495	1986586	55.6	49.1 (47.9, 50.4)
	Potentially preventable hospitalizations	Unexposed	2342	13642	3935436	21.7	19.0 (18.3, 19.7)
	Potentially preventable hospitalizations	Exposed	1230	6495	1986586	22.6	20.6 (19.6, 21.6)

	Unplanned hospitalizations	Unexposed	5073	13642	3935436	47.1	39.9 (39.0, 40.7)
	Unplanned hospitalizations	Exposed	2629	6495	1986586	48.3	42.8 (41.5, 44.0)
	Fall-related hospitalizations	Unexposed	1534	13642	3935436	14.2	12.6 (12.0, 13.2)
	Fall-related hospitalizations	Exposed	757	6495	1986586	13.9	12.8 (12.0, 13.7)
	Fracture-related hospitalizations	Unexposed	708	13642	3935436	6.6	5.9 (5.5, 6.3)
	Fracture-related hospitalizations	Exposed	342	6495	1986586	6.3	5.9 (5.3, 6.5)
	Medication-related adverse events	Unexposed	567	13642	3935436	5.3	4.7 (4.3, 5.1)
	Medication-related adverse events	Exposed	305	6495	1986586	5.6	5.2 (4.6, 5.8)
	Pressure injury hospitalizations	Unexposed	490	13642	3935436	4.5	4.0 (3.7, 4.4)
	Pressure injury hospitalizations	Exposed	180	6495	1986586	3.3	3.1 (2.6, 3.5)
	Malnutrition hospitalizations	Unexposed	737	13642	3935436	6.8	6.0 (5.6, 6.5)
	Malnutrition hospitalizations	Exposed	361	6495	1986586	6.6	6.1 (5.5, 6.7)
	Dementia hospitalizations	Unexposed	221	1968	545508	14.8	13.6 (12.0, 15.3)
	Dementia hospitalizations	Exposed	88	819	238707	13.5	12.5 (10.0, 15.0)
Continuity of Care - High (vs. Low)	Premature mortality	Unexposed	112	39742	13213567	0.3	0.4 (0.3, 0.5)
	Premature mortality	Exposed	41	16794	5409447	0.3	0.4 (0.3, 0.5)
	Mortality	Unexposed	2438	39742	13213567	6.7	8.4 (8.1, 8.7)
	Mortality	Exposed	1032	16794	5409447	7.0	8.6 (8.1, 9.1)
	Emergency department presentations	Unexposed	6020	13301	4536254	48.4	49.3 (48.4, 50.2)
	Emergency department presentations	Exposed	5909	14544	4672883	46.2	43.8 (43.0, 44.7)
	Potentially preventable hospitalizations	Unexposed	2489	13301	4536254	20.0	21.2 (20.5, 22.0)
	Potentially preventable hospitalizations	Exposed	2456	14544	4672883	19.2	18.9 (18.2, 19.5)
	Unplanned hospitalizations	Unexposed	5533	13301	4536254	44.5	45.1 (44.2, 46.0)
	Unplanned hospitalizations	Exposed	5423	14544	4672883	42.4	40.2 (39.3, 41.0)
	Fall-related hospitalizations	Unexposed	1850	13301	4536254	14.9	15.9 (15.2, 16.5)
	Fall-related hospitalizations	Exposed	1767	14544	4672883	13.8	13.8 (13.2, 14.4)
	Fracture-related hospitalizations	Unexposed	817	13301	4536254	6.6	7.1 (6.6, 7.5)
	Fracture-related hospitalizations	Exposed	767	14544	4672883	6.0	6.0 (5.6, 6.5)
	Medication-related adverse events	Unexposed	525	13301	4536254	4.2	4.6 (4.2, 5.0)

	Medication-related adverse events	Exposed	551	14544	4672883	4.3	4.3 (4.0, 4.7)
	Pressure injury hospitalizations	Unexposed	597	13301	4536254	4.8	5.2 (4.8, 5.6)
	Pressure injury hospitalizations	Exposed	639	14544	4672883	5.0	5.0 (4.6, 5.4)
	Malnutrition hospitalizations	Unexposed	789	13301	4536254	6.3	6.8 (6.3, 7.2)
	Malnutrition hospitalizations	Exposed	794	14544	4672883	6.2	6.2 (5.8, 6.6)
	Dementia hospitalizations	Unexposed	316	2496	826982	13.9	15.7 (14.1, 17.3)
	Dementia hospitalizations	Exposed	264	2557	818528	11.8	12.8 (11.3, 14.3)
Continuity of Care - Medium (vs. Low)	Premature mortality	Unexposed	76	25206	8581734	0.3	0.4 (0.3, 0.5)
	Premature mortality	Exposed	62	25006	8120201	0.3	0.4 (0.3, 0.5)
	Mortality	Unexposed	1673	25206	8581734	7.1	8.3 (7.9, 8.6)
	Mortality	Exposed	1317	25006	8120201	5.9	8.1 (7.6, 8.5)
	Emergency department presentations	Unexposed	9748	21860	7448727	47.8	48.3 (47.6, 49.0)
	Emergency department presentations	Exposed	8147	21704	7045388	42.2	39.9 (39.2, 40.6)
	Potentially preventable hospitalizations	Unexposed	3996	21860	7448727	19.6	20.7 (20.1, 21.3)
	Potentially preventable hospitalizations	Exposed	3277	21704	7045388	17.0	16.5 (16.0, 17.0)
	Unplanned hospitalizations	Unexposed	8919	21860	7448727	43.7	44.1 (43.4, 44.8)
	Unplanned hospitalizations	Exposed	7383	21704	7045388	38.2	36.1 (35.4, 36.8)
	Fall-related hospitalizations	Unexposed	2989	21860	7448727	14.6	15.5 (15.0, 16.0)
	Fall-related hospitalizations	Exposed	2274	21704	7045388	11.8	11.6 (11.1, 12.0)
	Fracture-related hospitalizations	Unexposed	1333	21860	7448727	6.5	7.0 (6.6, 7.3)
	Fracture-related hospitalizations	Exposed	969	21704	7045388	5.0	5.0 (4.7, 5.3)
	Medication-related adverse events	Unexposed	821	21860	7448727	4.0	4.3 (4.0, 4.6)
	Medication-related adverse events	Exposed	676	21704	7045388	3.5	3.5 (3.2, 3.7)
	Pressure injury hospitalizations	Unexposed	977	21860	7448727	4.8	5.1 (4.8, 5.4)
	Pressure injury hospitalizations	Exposed	759	21704	7045388	3.9	3.9 (3.6, 4.1)
	Malnutrition hospitalizations	Unexposed	1282	21860	7448727	6.3	6.7 (6.3, 7.0)
	Malnutrition hospitalizations	Exposed	1024	21704	7045388	5.3	5.2 (4.9, 5.5)
	Dementia hospitalizations	Unexposed	496	4068	1349153	13.4	15.0 (13.8, 16.2)
	Dementia hospitalizations	Exposed	379	4022	1294703	10.7	11.1 (10.0, 12.1)

Pattern of Service Use – Class 3 vs. Class 1[1]	Premature mortality	Unexposed	10	3987	1455255	0.3	0.4 (0.1, 0.7)
	Premature mortality	Exposed	14	3987	1455255	0.4	0.6 (0.3, 0.9)
	Mortality	Unexposed	358	3987	1455255	9.0	12.2 (11.0, 13.5)
	Mortality	Exposed	228	3987	1455255	5.7	7.9 (6.9, 8.9)
	Emergency department presentations	Unexposed	1734	3610	1317650	48.0	53.2 (51.4, 54.9)
	Emergency department presentations	Exposed	1540	3610	1317650	42.7	44.8 (43.1, 46.4)
	Potentially preventable hospitalizations	Unexposed	809	3610	1317650	22.4	25.8 (24.3, 27.4)
	Potentially preventable hospitalizations	Exposed	663	3610	1317650	18.4	19.9 (18.5, 21.2)
	Unplanned hospitalizations	Unexposed	1656	3610	1317650	45.9	50.5 (48.7, 52.2)
	Unplanned hospitalizations	Exposed	1415	3610	1317650	39.2	41.1 (39.5, 42.8)
	Fall-related hospitalizations	Unexposed	525	3610	1317650	14.5	17.0 (15.7, 18.3)
	Fall-related hospitalizations	Exposed	493	3610	1317650	13.7	14.7 (13.5, 15.9)
	Fracture-related hospitalizations	Unexposed	216	3610	1317650	6.0	7.2 (6.2, 8.1)
	Fracture-related hospitalizations	Exposed	215	3610	1317650	6.0	6.5 (5.6, 7.3)
	Medication-related adverse events	Unexposed	171	3610	1317650	4.7	5.6 (4.8, 6.5)
	Medication-related adverse events	Exposed	134	3610	1317650	3.7	4.1 (3.4, 4.8)
	Pressure injury hospitalizations	Unexposed	214	3610	1317650	5.9	6.9 (6.0, 7.7)
	Pressure injury hospitalizations	Exposed	128	3610	1317650	3.5	3.9 (3.2, 4.6)
	Malnutrition hospitalizations	Unexposed	250	3610	1317650	6.9	8.1 (7.2, 9.1)
	Malnutrition hospitalizations	Exposed	187	3610	1317650	5.2	5.7 (4.9, 6.5)
	Dementia hospitalizations	Unexposed	89	657	239805	13.5	17.1 (13.8, 20.5)
	Dementia hospitalizations	Exposed	67	643	234695	10.4	12.0 (9.3, 14.7)
Pattern of Service Use – Class 3 vs. Class 2[1]	Premature mortality	Unexposed	50	16219	5919935	0.3	0.5 (0.3, 0.6)
	Premature mortality	Exposed	48	16070	5865550	0.3	0.4 (0.3, 0.5)
	Mortality	Unexposed	1028	16219	5919935	6.3	8.8 (8.3, 9.4)
	Mortality	Exposed	871	16070	5865550	5.4	7.4 (6.9, 7.9)
	Emergency department presentations	Unexposed	5406	13753	5019845	39.3	42.2 (41.3, 43.0)
	Emergency department presentations	Exposed	5736	13785	5031525	41.6	44.2 (43.3, 45.0)

	Potentially preventable hospitalizations	Unexposed	2122	13753	5019845	15.4	17.1 (16.4, 17.8)
	Potentially preventable hospitalizations	Exposed	2328	13785	5031525	16.9	18.5 (17.9, 19.2)
	Unplanned hospitalizations	Unexposed	5079	13753	5019845	36.9	39.6 (38.7, 40.4)
	Unplanned hospitalizations	Exposed	5166	13785	5031525	37.5	39.8 (38.9, 40.6)
	Fall-related hospitalizations	Unexposed	1644	13753	5019845	12.0	13.4 (12.8, 14.0)
	Fall-related hospitalizations	Exposed	1722	13785	5031525	12.5	13.8 (13.2, 14.4)
	Fracture-related hospitalizations	Unexposed	711	13753	5019845	5.2	5.9 (5.5, 6.3)
	Fracture-related hospitalizations	Exposed	712	13785	5031525	5.2	5.7 (5.3, 6.2)
	Medication-related adverse events	Unexposed	412	13753	5019845	3.0	3.4 (3.1, 3.7)
	Medication-related adverse events	Exposed	470	13785	5031525	3.4	3.8 (3.5, 4.2)
	Pressure injury hospitalizations	Unexposed	588	13753	5019845	4.3	4.8 (4.4, 5.2)
	Pressure injury hospitalizations	Exposed	490	13785	5031525	3.6	4.0 (3.6, 4.3)
	Malnutrition hospitalizations	Unexposed	765	13753	5019845	5.6	6.2 (5.8, 6.7)
	Malnutrition hospitalizations	Exposed	729	13785	5031525	5.3	5.9 (5.5, 6.3)
	Dementia hospitalizations	Unexposed	257	2660	970900	9.7	11.5 (10.1, 12.8)
	Dementia hospitalizations	Exposed	266	2562	935130	10.4	12.3 (10.9, 13.7)

1. Class 1: High overall primary care use. Class 2: Low overall primary care use. Class 3: Preventive primary care service use.

Supplementary Table 13. Associations Between Primary Health Care Services and Health Outcomes, Hazard Ratios and Sub-distribution Hazard Ratios and 95% Confidence Intervals

	Premature mortality[1]	Mortality[1]	ED presentation[2]	Unplanned hospitalization[2]	PP hospitalization[2]	Falls[2]	Fractures[2]	Medication related adverse events[2]	Delirium and dementia hospitalization[2]	Pressure injury[2]	Weight loss and malnutrition[2]
After-hours attendances	1.58 (1.18, 2.11)	1.84 (1.74, 1.95)	1.42 (1.40, 1.44)	1.53 (1.51, 1.55)	1.44 (1.40, 1.47)	1.34 (1.31, 1.38)	1.29 (1.24, 1.34)	1.49 (1.42, 1.56)	1.53 (1.43, 1.64)	1.56 (1.49, 1.63)	1.45 (1.39, 1.51)
Urgent after-hours attendances	1.94 (1.39, 2.72)	2.65 (2.49, 2.83)	1.81 (1.73, 1.88)	1.91 (1.83, 1.99)	1.73 (1.63, 1.84)	1.45 (1.34, 1.57)	1.62 (1.44, 1.81)	1.74 (1.52, 1.98)	1.82 (1.52, 2.17)	2.27 (2.02, 2.56)	1.88 (1.69, 2.10)
Health assessments	0.88 (0.64, 1.20)	0.76 (0.71, 0.82)	1.06 (1.05, 1.08)	1.02 (1.00, 1.03)	1.02 (1.00, 1.05)	1.03 (1.00, 1.06)	0.99 (0.95, 1.04)	1.00 (0.95, 1.06)	0.95 (0.88, 1.03)	0.85 (0.81, 0.90)	0.91 (0.87, 0.96)
Management plans	0.64 (0.50, 0.83)	0.63 (0.60, 0.67)	1.13 (1.12, 1.15)	1.11 (1.09, 1.13)	1.15 (1.13, 1.18)	1.12 (1.09, 1.15)	1.06 (1.02, 1.11)	1.15 (1.10, 1.21)	0.94 (0.88, 1.00)	0.88 (0.84, 0.92)	0.93 (0.89, 0.97)
Allied health – Podiatry	0.71 (0.52, 0.97)	0.60 (0.56, 0.63)	1.02 (1.00, 1.04)	1.00 (0.97, 1.02)	1.05 (1.01, 1.08)	1.05 (1.01, 1.09)	0.95 (0.89, 1.01)	1.05 (0.98, 1.13)	0.98 (0.88, 1.08)	0.78 (0.73, 0.84)	0.84 (0.79, 0.89)
Allied health - Optometric	0.61 (0.46, 0.80)	0.53 (0.50, 0.56)	1.05 (1.03, 1.08)	1.01 (0.98, 1.03)	1.02 (0.99, 1.06)	0.98 (0.93, 1.02)	0.95 (0.89, 1.02)	1.02 (0.95, 1.11)	0.82 (0.71, 0.93)	0.66 (0.61, 0.72)	0.81 (0.76, 0.87)
Mental health attendances	1.35 (0.81, 2.26)	0.87 (0.77, 0.98)	1.13 (1.08, 1.18)	1.11 (1.06, 1.16)	1.11 (1.03, 1.19)	1.04 (0.95, 1.13)	1.02 (0.90, 1.16)	1.10 (0.95, 1.26)	0.93 (0.73, 1.20)	0.76 (0.64, 0.90)	1.02 (0.90, 1.16)
Continuity of care - Usual GP vs. New	0.90 (0.62, 1.29)	1.02 (0.95, 1.10)	0.93 (0.90, 0.96)	0.93 (0.90, 0.96)	0.93 (0.89, 0.98)	0.94 (0.89, 1.00)	0.94 (0.87, 1.02)	1.01 (0.91, 1.11)	0.94 (0.82, 1.09)	1.04 (0.94, 1.14)	0.96 (0.88, 1.04)
Continuity of care - Known GP vs. New	0.97 (0.69, 1.35)	0.94 (0.87, 1.01)	0.80 (0.77, 0.82)	0.80 (0.77, 0.82)	0.81 (0.78, 0.85)	0.74 (0.70, 0.79)	0.72 (0.66, 0.78)	0.82 (0.74, 0.91)	0.74 (0.65, 0.85)	0.78 (0.71, 0.85)	0.79 (0.73, 0.86)
Patterns of health care utilization: Class 3 vs. Class 1[3]	1.20 (0.53, 2.74)	0.59 (0.50, 0.70)	0.85 (0.80, 0.92)	0.81 (0.75, 0.87)	0.84 (0.76, 0.93)	0.97 (0.86, 1.10)	1.01 (0.83, 1.22)	0.84 (0.67, 1.05)	0.75 (0.54, 1.03)	0.64 (0.52, 0.80)	0.78 (0.64, 0.94)
Patterns of health care utilization: Class 3 vs. Class 2[3]	0.96 (0.64, 1.42)	0.83 (0.76, 0.91)	1.07 (1.03, 1.11)	1.02 (0.98, 1.06)	1.10 (1.04, 1.17)	1.05 (0.98, 1.12)	1.01 (0.91, 1.12)	1.15 (1.01, 1.31)	1.07 (0.90, 1.27)	0.84 (0.74, 0.94)	0.96 (0.86, 1.06)

ED = Emergency department. PP= Potentially preventable. GP = General Practitioner.

1. Hazard ratio and 95% confidence intervals.
2. Sub-distribution hazard ratios and 95% confidence intervals.
3. Class 1: High overall primary care use. Class 2: Low overall primary care use. Class 3: High preventive primary care service use.

Supplementary Figure 1. Patterns of Health Care Service Utilization by Latent Class Analysis - Identified Classes

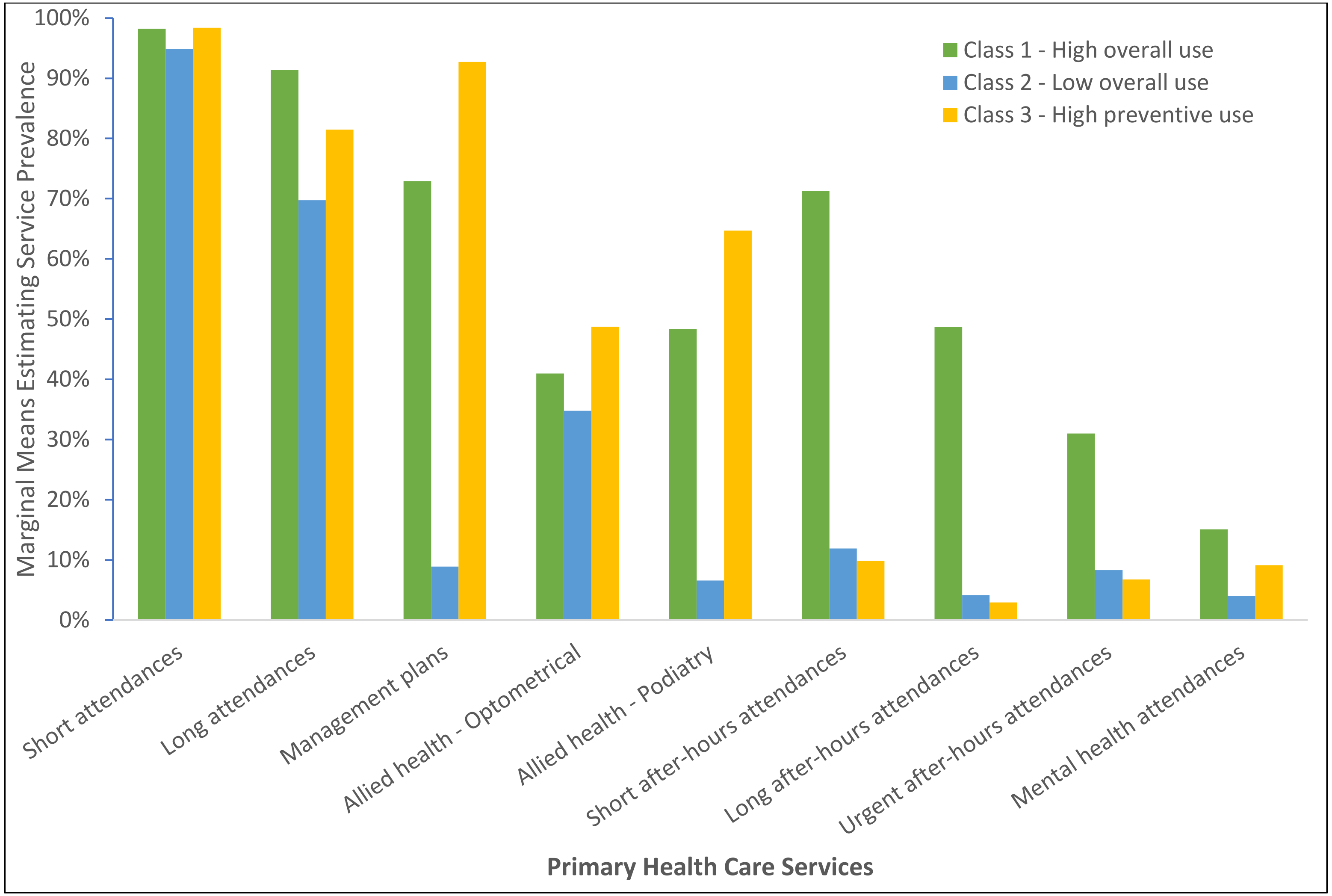

Supplementary Figure 2. Flow chart of the sensitivity analysis.

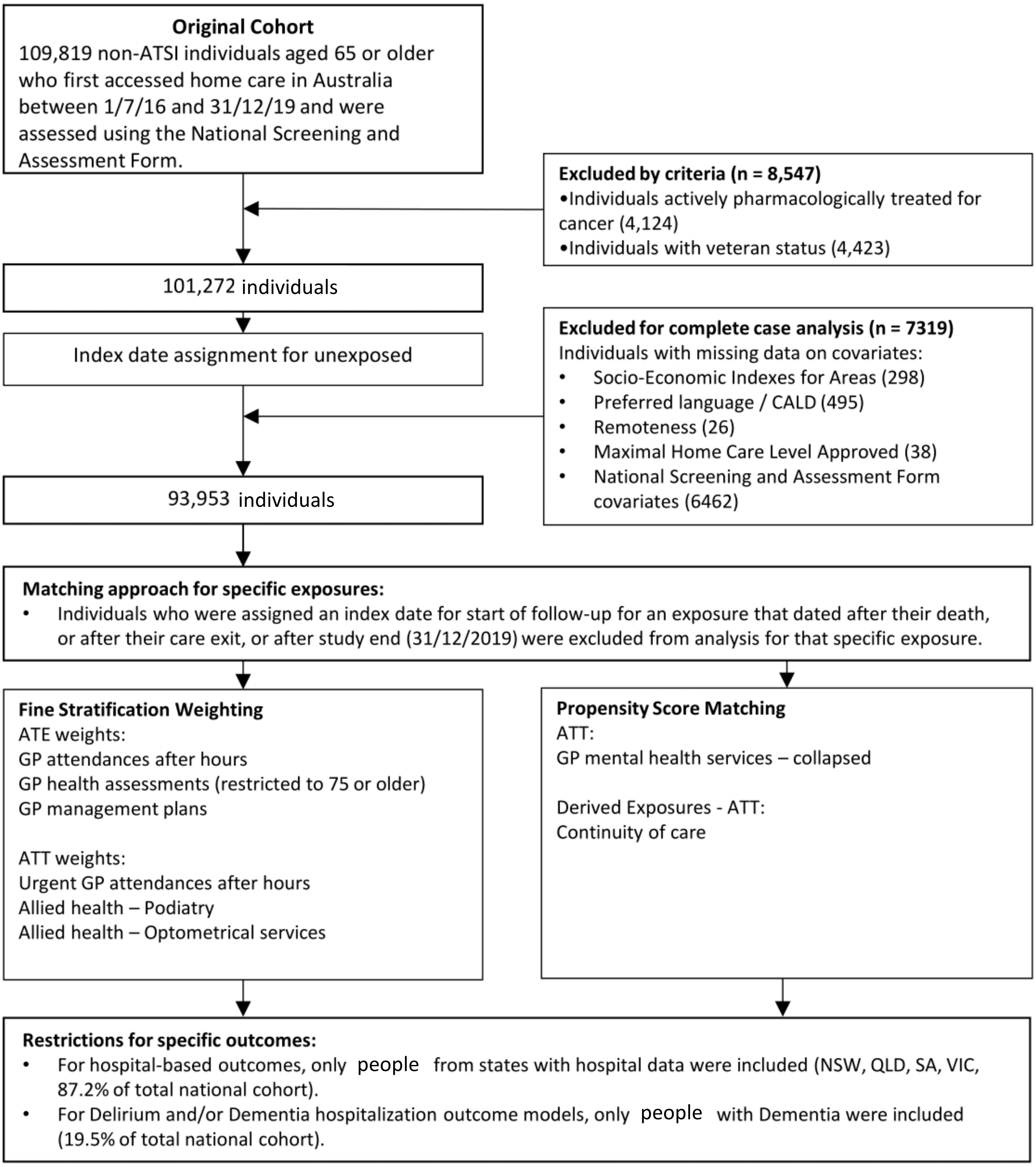

Supplementary Figure 3. Associations Between Specific Primary Care Services, Continuity of Care and Health Outcomes in Sensitivity Analysis by Additional Covariates and All Care Levels. Hazard ratios (mortality outcomes) and sub-distribution hazard ratios (other outcomes).

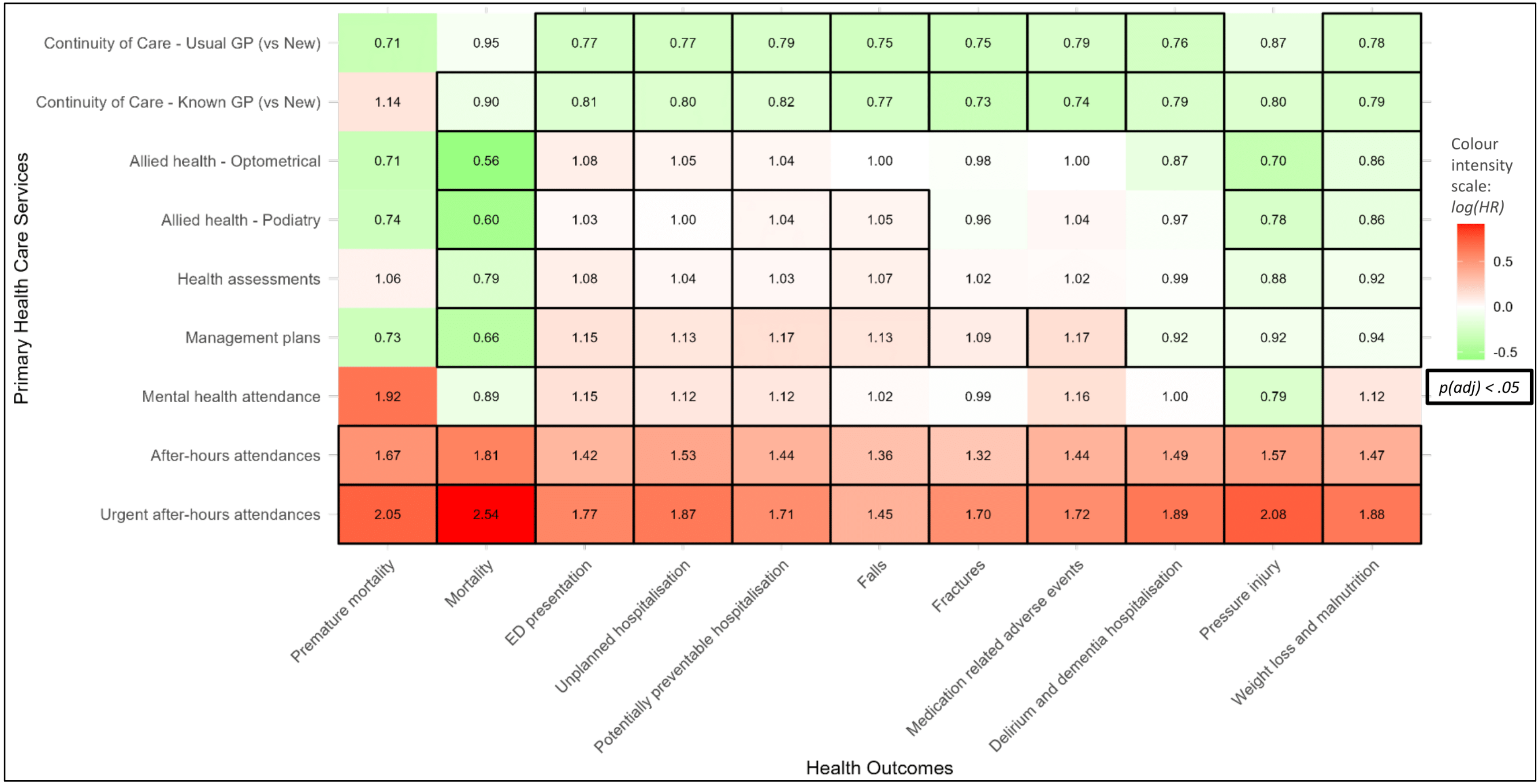

Legend: Estimate <1 (green): lower risk of experiencing the outcome within 1 year.
Estimate >=1 (red): higher risk of experiencing the outcome within 1 year.
Cells with black outlines: Statistically significant estimates (after correction for multiple hypothesis testing).
ED = Emergency Department. GP = General Practitioner.

Supplementary Figure 4. Associations Between Specific Primary Care Services, Continuity of Care and Health Outcomes in the Sensitivity Analysis by Home Care Levels 1 and 2. Hazard ratios (mortality outcomes) and sub-distribution hazard ratios (other outcomes).

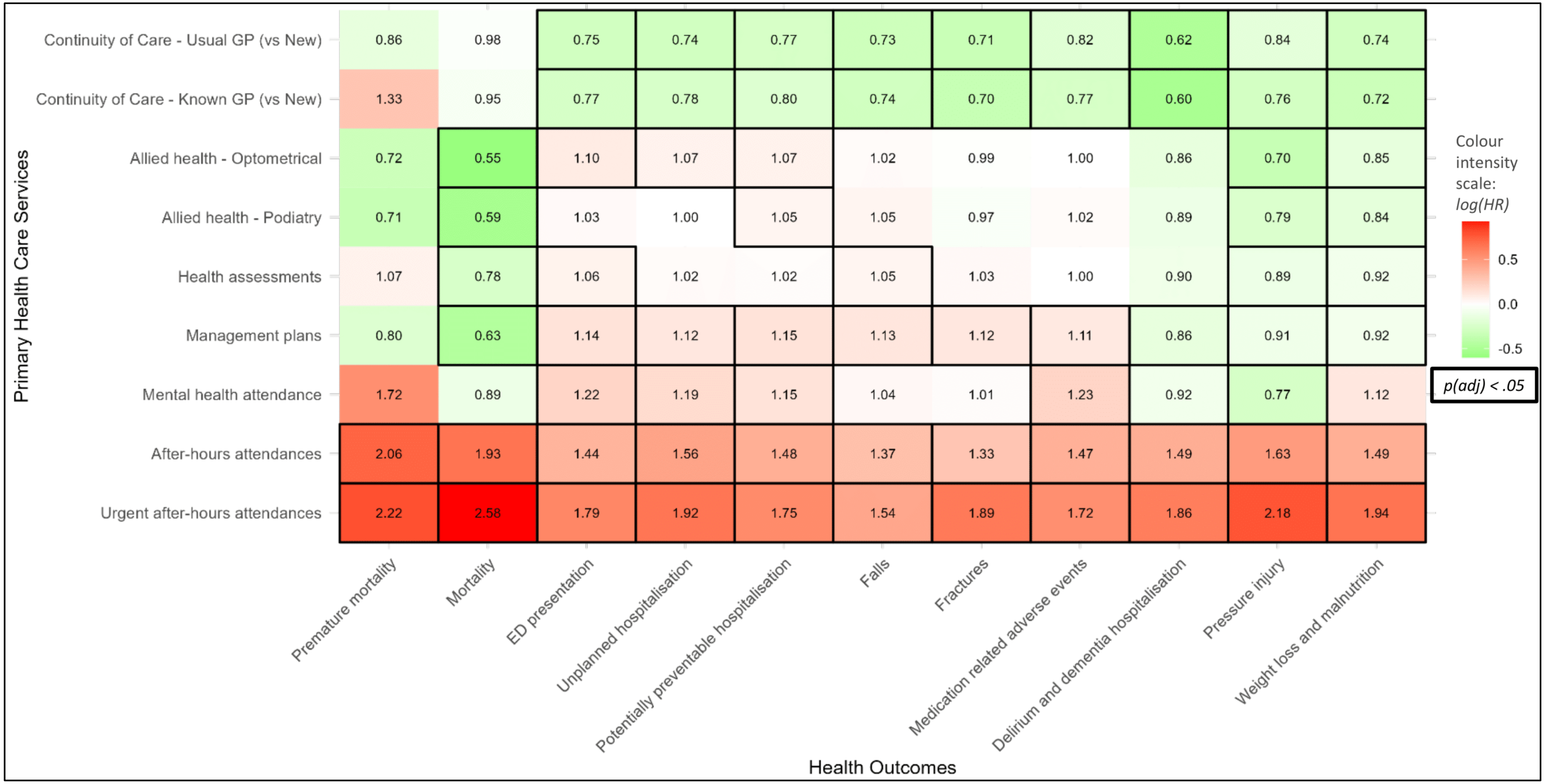

	Premature mortality	Mortality	ED presentation	Unplanned hospitalisation	Potentially preventable hospitalisation	Falls	Fractures	Medication related adverse events	Delirium and dementia hospitalisation	Pressure injury	Weight loss and malnutrition
Continuity of Care - Usual GP (vs New)	0.86	0.98	0.75	0.74	0.77	0.73	0.71	0.82	0.62	0.84	0.74
Continuity of Care - Known GP (vs New)	1.33	0.95	0.77	0.78	0.80	0.74	0.70	0.77	0.60	0.76	0.72
Allied health - Optometrical	0.72	0.55	1.10	1.07	1.07	1.02	0.99	1.00	0.86	0.70	0.85
Allied health - Podiatry	0.71	0.59	1.03	1.00	1.05	1.05	0.97	1.02	0.89	0.79	0.84
Health assessments	1.07	0.78	1.06	1.02	1.02	1.05	1.03	1.00	0.90	0.89	0.92
Management plans	0.80	0.63	1.14	1.12	1.15	1.13	1.12	1.11	0.86	0.91	0.92
Mental health attendance	1.72	0.89	1.22	1.19	1.15	1.04	1.01	1.23	0.92	0.77	1.12
After-hours attendances	2.06	1.93	1.44	1.56	1.48	1.37	1.33	1.47	1.49	1.63	1.49
Urgent after-hours attendances	2.22	2.58	1.79	1.92	1.75	1.54	1.89	1.72	1.86	2.18	1.94

Legend: Estimate <1 (green): lower risk of experiencing the outcome within 1 year.
Estimate >=1 (red): higher risk of experiencing the outcome within 1 year.
Cells with black outlines: Statistically significant estimates (after correction for multiple hypothesis testing).
ED = Emergency Department. GP = General Practitioner.

Supplementary Figure 5. Associations Between Specific Primary Care Services, Continuity of Care and Health Outcomes in the Sensitivity Analysis by Home Care Levels 3 and 4. Hazard ratios (mortality outcomes) and sub-distribution hazard ratios (other outcomes).

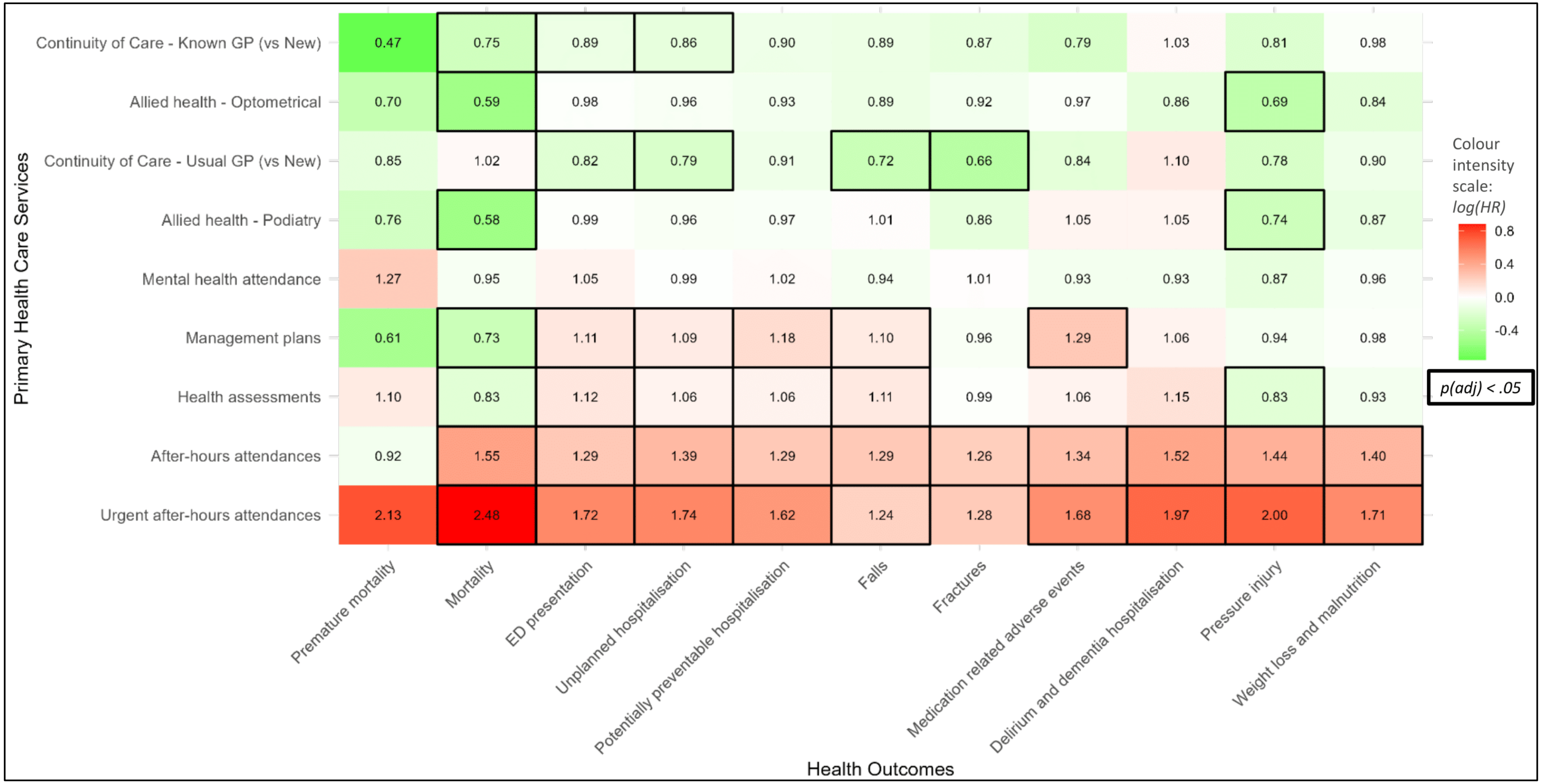

Legend: Estimate <1 (green): lower risk of experiencing the outcome within 1 year.
Estimate >=1 (red): higher risk of experiencing the outcome within 1 year.
Cells with black outlines: Statistically significant estimates (after correction for multiple hypothesis testing).
ED = Emergency Department. GP = General Practitioner.

Supplementary Figure 6. Associations Between GP Management Plans and Health Outcomes in the Sensitivity Analysis Stratifying by Dementia and Diabetes. Hazard ratios (mortality outcomes) and sub-distribution hazard ratios (other outcomes).

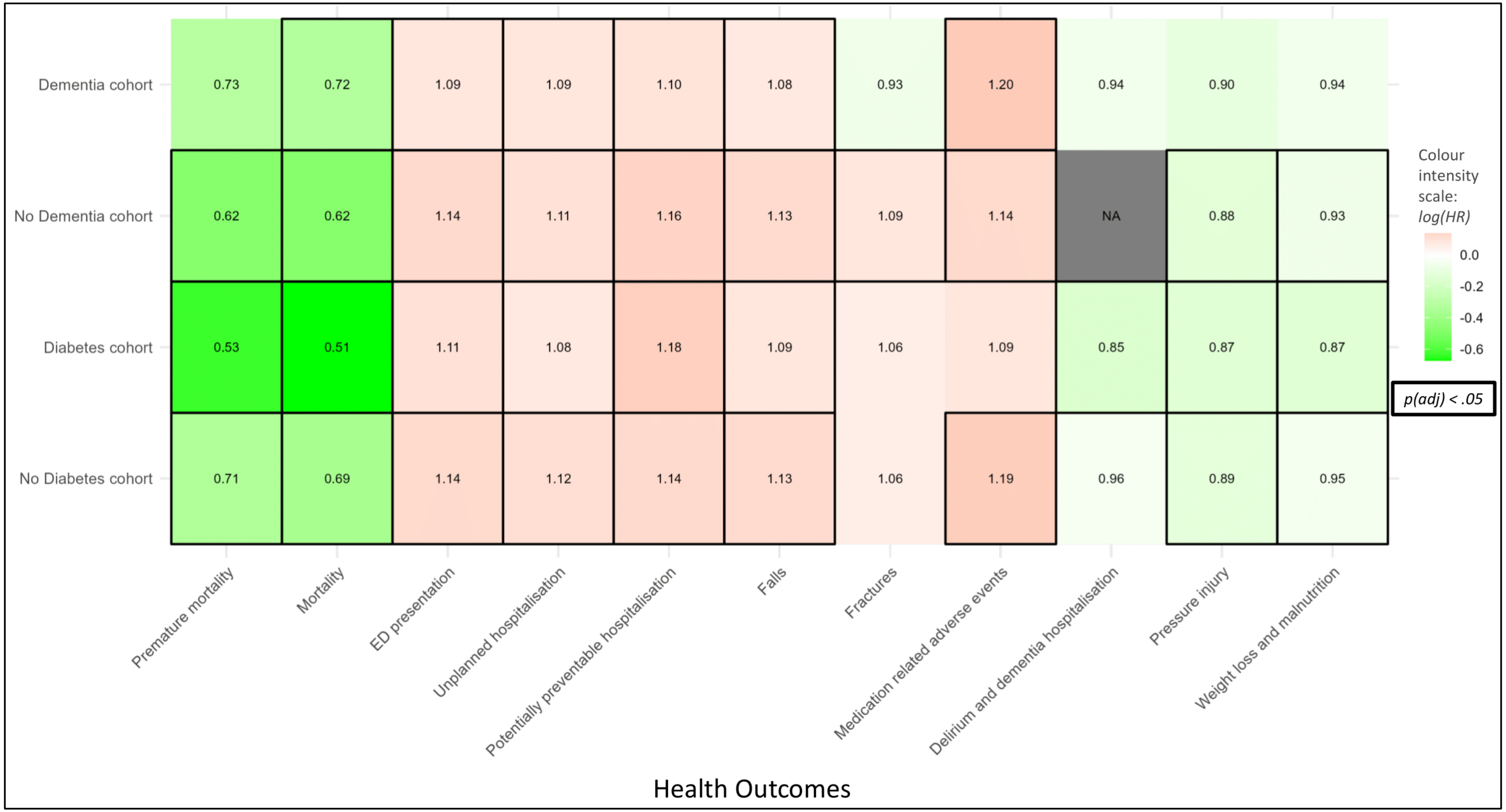

Legend: Estimate <1 (green): lower risk of experiencing the outcome within 1 year.
Estimate >=1 (red): higher risk of experiencing the outcome within 1 year.
Cells with black outlines: Statistically significant estimates (after correction for multiple hypothesis testing).
ED = Emergency Department. GP = General Practitioner.

Supplementary Figure 7. Associations Between Specific Primary Care Services and Health Outcomes in the Sensitivity Analysis Delaying Outcome Ascertainment to 30 days Post-exposure Period. Hazard ratios (mortality outcomes) and sub-distribution hazard ratios (other outcomes).

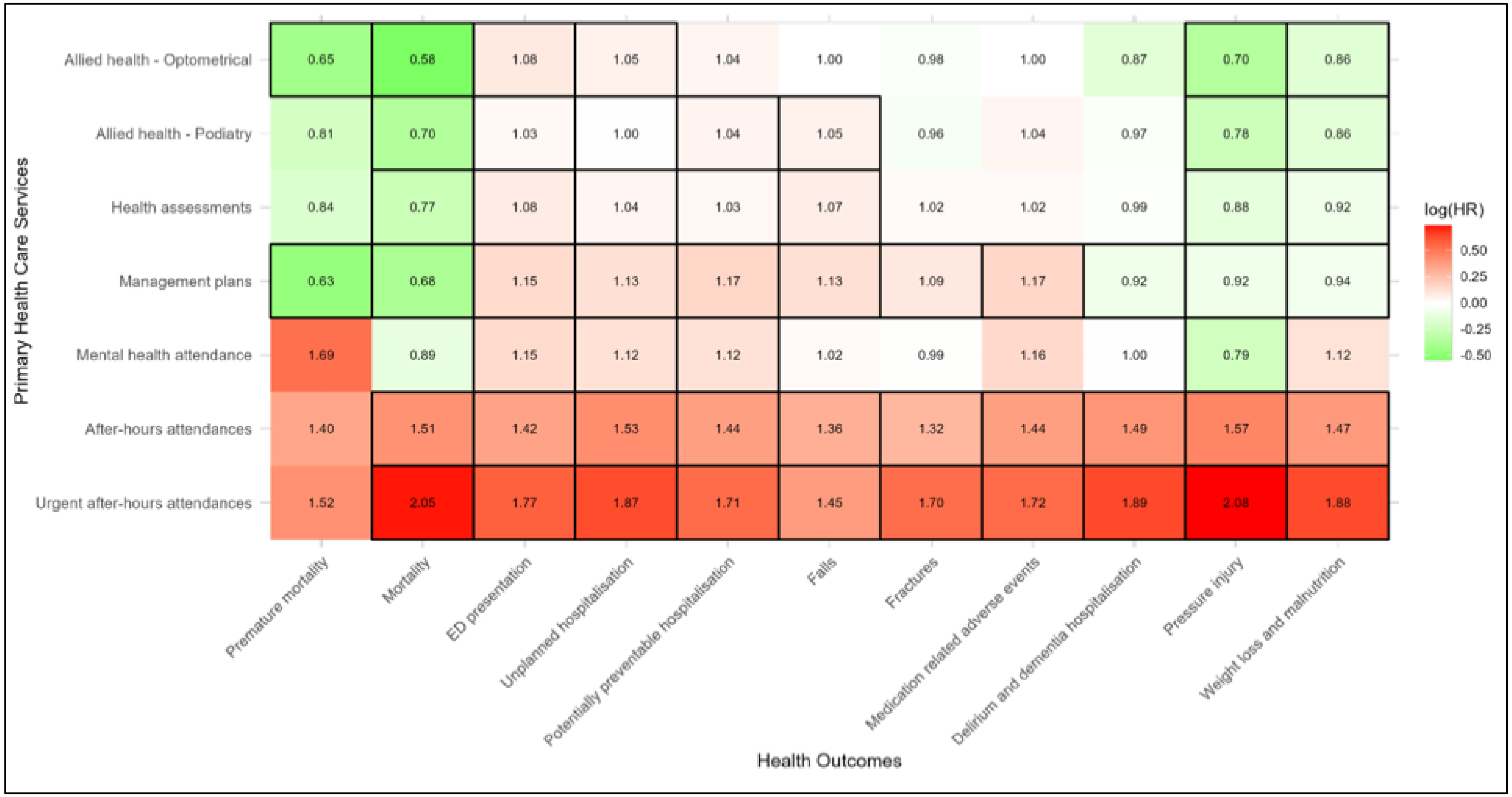

Legend: Estimate <1 (green): lower risk of experiencing the outcome within 1 year.
Estimate >=1 (red): higher risk of experiencing the outcome within 1 year.
Cells with black outlines: Statistically significant estimates (after correction for multiple hypothesis testing).
ED = Emergency Department. GP = General Practitioner.
